# Variational relevance evaluation of individual fMRI data enables deconstruction of task-dependent neural dynamics

**DOI:** 10.1038/s42003-023-04804-3

**Published:** 2023-05-05

**Authors:** Xiaoyu Lv, Shintaro Funahashi, Chunlin Li, Jinglong Wu

**Affiliations:** 1 School of Mechatronical Engineering, Beijing Institute of Technology, Beijing, China; 2 Advanced Research Institute of Multidisciplinary Science, Beijing Institute of Technology, Beijing, China; 3 School of Biomedical Engineering, Capital Medical University, Beijing, China; 4 Beijing Key Laboratory of Fundamental Research on Biomechanics in Clinical Application, Capital Medical University, Beijing, China; 5 School of Medical Technology, Beijing Institute of Technology, Beijing, China; 6 Researh Center for Medical Artificial Intelligence, Shenzhen Institute of Advanced Technology, Chinese Academy of Science, Shenzhen, Guangdong China

**Keywords:** Computational neuroscience, Functional magnetic resonance imaging

## Abstract

In neuroimaging research, univariate analysis has always been used to localize “representations” at the microscale, whereas network approaches have been applied to characterize transregional “operations”. How are representations and operations linked through dynamic interactions? We developed the variational relevance evaluation (VRE) method to analyze individual task fMRI data, which selects informative voxels during model training to localize the “representation”, and quantifies the dynamic contributions of single voxels across the whole-brain to different cognitive functions to characterize the “operation”. Using 15 individual fMRI data files for higher visual area localizers, we evaluated the characterization of selected voxel positions of VRE and revealed different object-selective regions functioning in similar dynamics. Using another 15 individual fMRI data files for memory retrieval after offline learning, we found similar task-related regions working in different neural dynamics for tasks with diverse familiarities. VRE demonstrates a promising horizon in individual fMRI research.

## Abstract (web summary)

The variational relevance evaluation (VRE) method is developed to analyze individual task fMRI data and characterise task-specific neural interactions.

## Introduction

It is widely accepted that a full theory of any cognition requires both representations and operations, especially high-level cognitive functions. In cognitive neuroscience research, “representations” analysis approaches ascribe distinct cognitive processes to individual brain regions^1^. In functional MRI (fMRI) studies, a common “representation” approach is univariate analysis, which regards voxels independently. This approach can be carried out with patterns of large spatial scale, including those derived from whole-brain fMRI activity. However, there are limits on what can be learned about cognitive states by examining voxels in isolation^2^, as this could result in discarding the spatial relationships among cortical locations^3^. In particular, the spatial averaging method prunes voxels with weaker responses to a particular condition that might carry some information about the presence/absence of that condition and blurs out fine-grained spatial patterns that might discriminate between experimental conditions^4^.

In effect, cooperation and effective communication between spatially separate neural regions play a crucial role in the execution of many cognitive behaviors^5^. A series of approaches have evolved to functionally label brain networks rather than focal brain regions such that “operation” characterization is possible. A “network” describes spatially distinct brain regions, or nodes, that are functionally or structurally connected through edges^6^. Methods for identifying brain networks vary by spatial scale, temporal scale, and relation to cognition, but most utilize covariation in brain activity across time or experimental trials^7,8^. Considerable interest has arisen in resting-state networks (RSNs), such as the default mode network (DMN) and the salience network;^9–11^ and in large-scale connectivity-based, task-related networks (cTRNs). While RSNs are defined as regions that tend to be functionally related during rest and cTRNs link the network topology to behavior directly with a similar logic as RSNs, neither can specify the corresponding contributions of each region or the flow of information and control across regions during a task^12^. Compared to univariate fMRI analysis, functional connectivity realizes interaction analysis based on brainwide regions at the cost of voxelwise analysis, which makes it impossible to separate the function-responsive representation across single regions at the microscale, such as single voxels.

Neither conventional “representation” nor “operation” approaches can answer the central question: how the operations and representations are linked through dynamic interactions at the microscale? Moreover, identifying the unique functional architecture of an individual’s brain is crucial to understand the neural basis of variation in human cognition and behavior^13^. However, the overfitting problem due to high-dimensionality and minor-dataset characteristics of fMRI data has become a major obstacle for methodology innovation in neuroscience studies, especially for research based on individual datasets that are collected via conventional paradigms. To address this, we need a representation-operation hybrid approach based on individual data to characterize how the brain is carved up in terms of its dynamic contributions to cognition functioning at the microscale is needed.

**Fig. 1:**
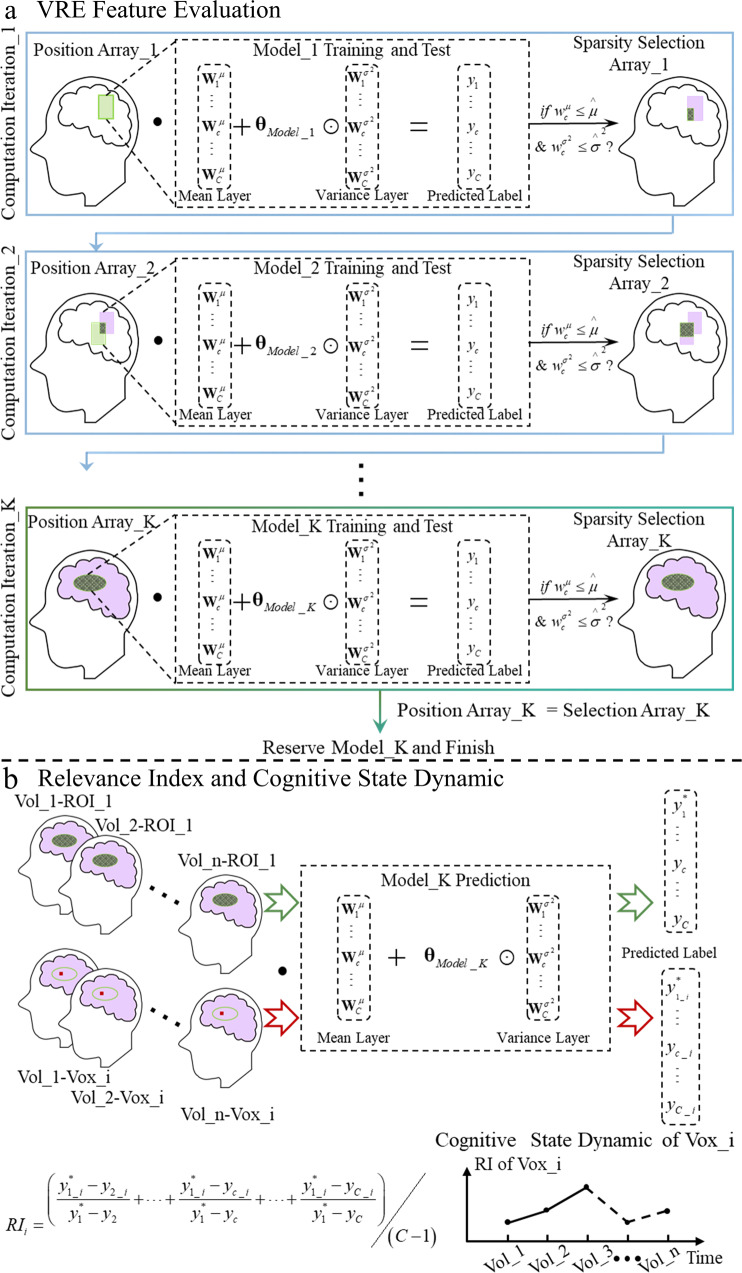
Overview of VRE feature evaluation and Relevance Index. **a** VRE feature evaluation. Multi-linear classification models matching the feature batch dimensionality need to be trained to predict the correct stimulus labels. According to the weights of the model, the Sparsity Position Array distinguishing the informative feature positions can be obtained. To guarantee the feature evaluation efficiency, the Position Array is composed of the positions of both productive features evaluated by the last computation iteration (dotted part) and supplementary features not analyzed before (green part) to meet the features maximum analyzed in one iteration. Reserving the model when the Position Array and Sparsity Selection Array of one computation iteration are the same as each other, denoting the linear relation between the least number of features and labels is established. The Sparsity Selection Array evaluated from the parameters of the reserved model signifies the ROI for each stimulus condition. The green part signifies the positions of features not analyzed before, the purple part signifies the positions of unproductive features evaluated before, and the dotted part signifies the positions of informative features evaluated before. $\odot$ signifies the Hadamard product. $\bullet$ denotes the dot product. **b** Relevance Index (RI) and Cognitive Stage Dynamic. Vol_1–Vol_n are *n* volumes collected sequentially during one stimulus condition (corresponding to the correct category label element ${y}_{1}^{\ast }$). The dotted part signifies the positions of informative voxels for the stimulus condition evaluated from Model_K parameters, namely Sparsity Selection Array_K in Fig. 1a. The red dot Vox_i denotes each voxel of the dotted part. The label consists of C elements, with each corresponding to one category. The mean of (C-1) proportions, with each denoting the ratio of difference predicted from one informative voxel between correct category label element (${y}_{1\_i}^{\ast }$) and another element, to the similar difference predicted from all informative voxels, is the RI of Vol_n-Vox_i. RI is defined to quantify the contribution of every single voxel to the correct classification. The Cognitive State Dynamic of Vox_i is arranged temporally by the RIs analyzed from *n* volumes collected sequentially (Vol_1–Vol_n).

We developed variational relevance evaluation (VRE, shown in Fig. 1a) and the relevance index (RI, shown in Fig. 1b) to identify brain representations for stimulus condition and cognitive state dynamics (dynamic, in short) characterizing brainwide contribution variety in the temporal domain at the level of single voxels for individuals, based on the assumption that the brain works as an entirety with sophisticated parts that cooperate to perform disparate cognitive functions^14^. Within the variational Bayesian (VB) framework, we develop the batch-feature-input trick to avoid overfitting due to the simultaneous analysis of a large number of voxels, and two adversarial strategies are performed by two parallel linear layers to concurrently conduct feature evaluation and model training. Intriguingly, only two fully connected layers are recruited to perform linear classification in this paper. For this, we agree with the perspective proposed by ref. ^15^, who stated that all brain processes essentially reflect a series of nonlinear computations; it is pivotal to avoid adding additional nonlinear steps^16^ to characterize the information processed by a brain region.

In this research, we first evaluated the classification performance and the characterization of selected voxel positions of VRE using 15 individual fMRI data files for higher-visual area localizers^17^. The results show that VRE selects voxels with location distributions that are consistent with known functional anatomy for disparate categories with reasonable classification accuracy. Furthermore, dynamic research demonstrates that different object-selective regions function in similar patterns for distinct visual stimulus conditions. Then, using another 15 individual fMRI data files for memory retrieval after offline learning^18^, we found three dynamics in the dorsal lateral prefrontal cortex (DLPFC), bilateral sensorimotor area, medial temporal lobe (MTL), and vision association area across four different tasks with diverse degrees of familiarity for subjects (offline improvements occurred in one task with others evolving from the task). Subjects with distinct offline learning abilities showed different neural dynamics across the performance of the trained task. When analyzing the dynamics of single regions, we conclude that progressive trends are correlated with task contents.

## Results

### Overview of VRE feature evaluation and relevance index

To select the most informative features linearly related to stimulus conditions from the whole-brain, we propose to evaluate the validity of features batch by batch. As shown in Fig. 1, VRE feature evaluation involves multiple computation iterations, each with a classification model composed of two parallel linear layers. First, the features batch awaiting evaluation needs to be confirmed by the mask with green background. Second, a linear classification model matching the dimensionality of the batch is trained to predict the label of the corresponding stimulus condition. Third, the Sparsity Selection Array is transformed from the Position Array, and the position of a feature is set to zero if all weights related to the feature meet the predefined criteria (${w}_{c}^{\mu }\le \mathop{\mu }\limits^{\wedge }$ for weights from the mean layer, and ${w}_{c}^{{\sigma }^{2}}\le {\mathop{\sigma }\limits^{\wedge }}^{2}$ for weights from the variance layer), as shown in the purple part of Fig. 1. The dotted part denotes the informative feature positions evaluated in the iteration. To guarantee efficiency, we set max_fea (equal to the size denoted by Position Array_1) as the maximum number of features computed spontaneously. The feature quantity evaluated in each iteration should meet max_fea if the features not analyzed are sufficient for complement. According to the Sparsity Selection Array evaluated in the last iteration, the Position Array of the feature batch awaiting evaluation in the next iteration is confirmed by the positions of informative features evaluated before (dotted part) and that of new features (green part). Finally, the VRE feature validity evaluation is not complete until the Position Array and Sparsity Selection Array in one computation iteration are the same, which denotes that the least number of informative features is confirmed to guarantee the model convergence. Then, the model of the last computation iteration is reserved. With the Sparsity Selection Array of the reserved model, the brain representation for each condition is obtained. Throughout the paper, vectors and scalars are signified in bold-faced letters and normal face letters, respectively. “Features” denote BOLD responses of voxels.

The relevance index (RI) is defined to quantify the contribution of every single feature to the correct classification. Using the reserved model, we can predict the label of the volume while only one informative voxel is considered (the red dot shown in Fig. 1b). The RI for the voxel is the mean of the (C-1) proportions, with each signifying the ratio of the difference between the correct category label element (${y}_{1\_i}^{*}$ in Fig. 1b) and one of the other alternatives predicted from the voxel to the similar difference predicted from all informative voxels. The cognitive state dynamic for one voxel is arranged temporally by the RIs estimated from volumes of one condition collected sequentially. The pattern analysis mentioned here was based on a single brain scan, which is accessible only if the cognitive states expressed by the selected features are sufficiently disparate from one another for model building^19–28^.

Different brain regions may work in distinct patterns. In regard to a single voxel cluster selected by VRE, voxels within the cluster may have distinct dynamics. To identify the dominating pattern of each voxel cluster compatible with most voxels in the cluster, we applied two-category hierarchical clustering to the dynamics of the voxels from a single cluster selected by VRE. It should be noted that the dynamics of the voxel cluster researched in this paper represent the dominant cognitive state dynamics.

### Localizer for higher-visual areas

We use the data from the StudyForrest dataset^17^ to test whether the locations of VRE-selected voxels are consistent with known functional anatomy, and explore the representation and operations, involved in higher-visual object-selective cognition. Subjects were required to view the pictures of 6 categories sequentially in a block experimental design.

**Fig. 2:**
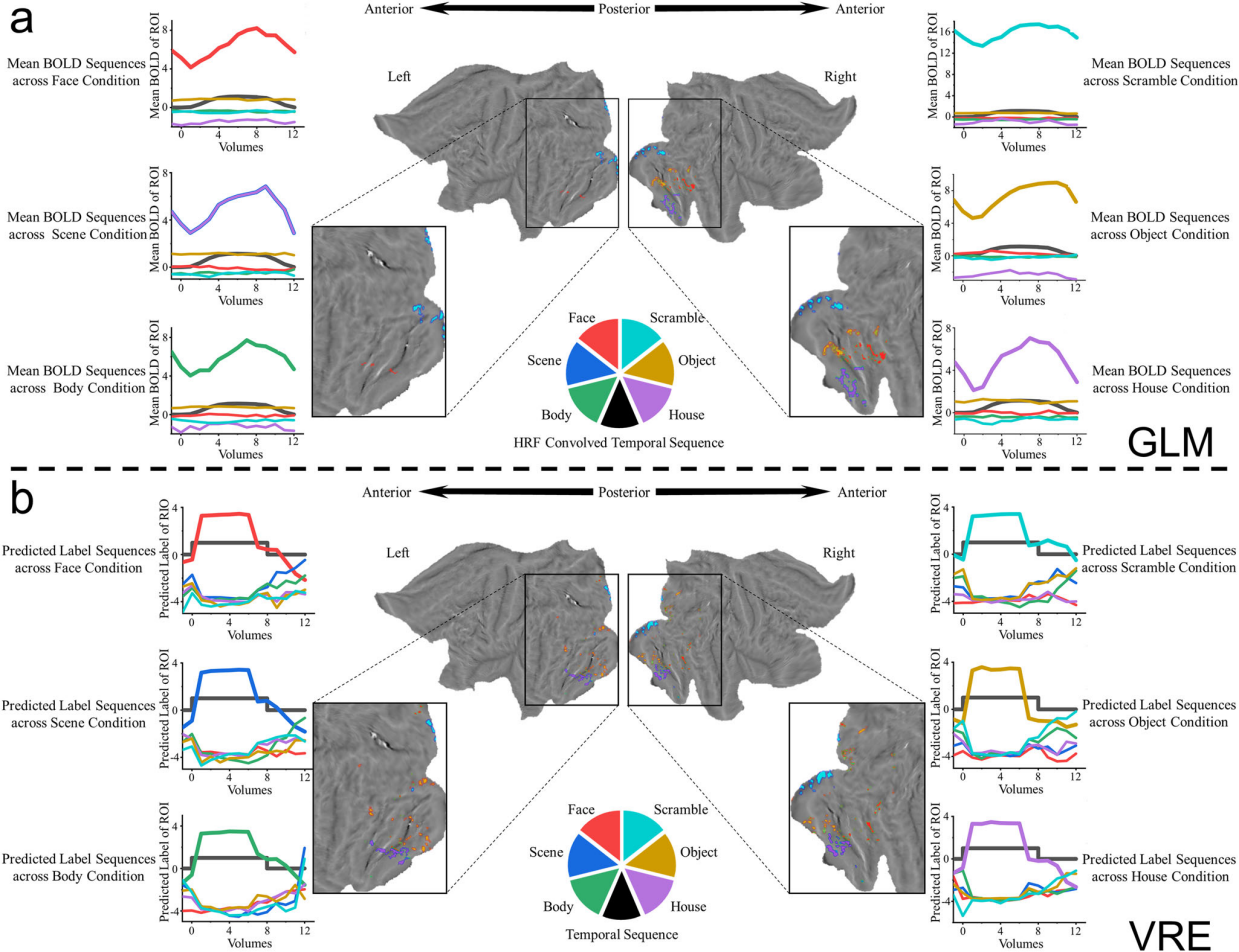
Individual object-selective results selected by the GLM and VRE for Subject_01. The six different colors in the pie color bar indicate the corresponding task conditions, and the bottom black color signifies the “label” (HRF-convolved stimulus temporal sequence for the GLM; stimulus temporal sequence for the VRE) used to select the features. The colored topology mapping on the flat surface denotes the features selected by the GLM and VRE under the six conditions. The 0 point on the x-axis of the line charts indicates the volume gathered at stimulus onset, and 14 continuous volumes obtained during the stimulus presentation block were included in the analysis (the first two volumes collected before, the eight volumes gathered during, and the last four volumes collected after stimulus presentation). **a** GLM-selected feature topology distribution of the six stimulus conditions; six line charts of block-averaged, voxel-mean BOLD responses of every object-selective region (colored bold lines) versus the corresponding HRF-convolved stimulus temporal sequences occurring during these blocks (black bold lines). **b** VRE-selected feature topology distribution of the six stimulus conditions (every column of Sparsity Selection Array evaluated from a reserved model in Fig. 1a signifies the ROI for each condition); six line charts of block-averaged predicted label element (${y}_{c}$ in Fig. 1b) sequence for every object-selective region (colored bold lines) versus the corresponding stimulus temporal sequences occurring during these blocks (black bold lines).

To test the position accuracy and specific condition exclusiveness of the selected voxels, we applied the VRE method to these fMRI data. A series of 6-class individual classification models based on the VRE framework were constructed with brainwide voxels. Classification accuracy reflects the extent of valid information mined in the brainwide voxels for discriminating among the experimental conditions tested^16^. The related models finally achieved ideal accuracy (98.6 $\pm$ 1.1%, average accuracy and standard deviation across all models), guaranteeing meaningful feature evaluation. Note that there are six different linear classification functions, implying six disparate stimulus conditions with one voxel selection for every condition. Then, we compared the activated voxels selected by the VRE model with those selected with the general linear model (GLM) from the Functional Magnetic Resonance Imaging of the Brain (FMRIB) Software Library (FSL, provided by the original paper) relevant to every experimental condition. For both approaches used to select meaningful features, “labels” are needed. Label functions were fulfilled here by stimulus temporal sequences and hemodynamic response function (HRF)-convolved stimulus temporal sequences for the VRE and GLM, respectively. The middle parts of Fig. 2a, b illustrate the topology mapping of Subject_01 on a flat surface under different conditions selected by the GLM and VRE model. Substantial distribution consistency was achieved between the voxels selected by the VRE model and those of the previous study^29^ regarding object-selective regions. As the six line charts in Fig. 2a depict, the block-mean, voxel-mean BOLD responses across five regions of interest (ROIs) constitute the BOLD temporal sequences for five task conditions (House and Scene are both regarded as the Place category in the original paper), and 14 continuous volumes (2 volumes before, 8 volumes during, and 4 volumes after stimulus presentation; volume 0 corresponds to stimulus onset) alongside one specific stimulus implementation are shown on the six line charts in Fig. 2a. For VRE, one predicted label element (${y}_{c}$ in Fig. 1b) of the corresponding ROI can be predicted by the linear function established for every condition after model convergence. As Fig. 2b denotes, the block-mean predicted label elements from the same 14 continuous volumes constitute the temporal sequence, and the sequences for different conditions are shown on the six line charts in Fig. 2b. Every line chart contains two bold lines: the black line is the HRF-convolved stimulus temporal sequence or stimulus temporal sequence of the corresponding task occurring during these blocks, and the colored line is the correct object-selective ROI result (e.g., the black line signifies the HRF-convolved temporal sequence of face condition, and the red line on the same chart is the averaged BOLD sequences of the face-selective ROI selected by the GLM in the upper-left chart of Fig. 2a). From Fig. 2a, b, we can conclude that the correct label element sequences of the VRE are in substantial accordance with the corresponding stimulus condition relative to other sequences. In addition, the individual results of the remaining 14 subjects are presented in Supplementary Figs. 1–14. In other words, the VRE can achieve a discriminative classification result for a specific condition.

**Fig. 3:**
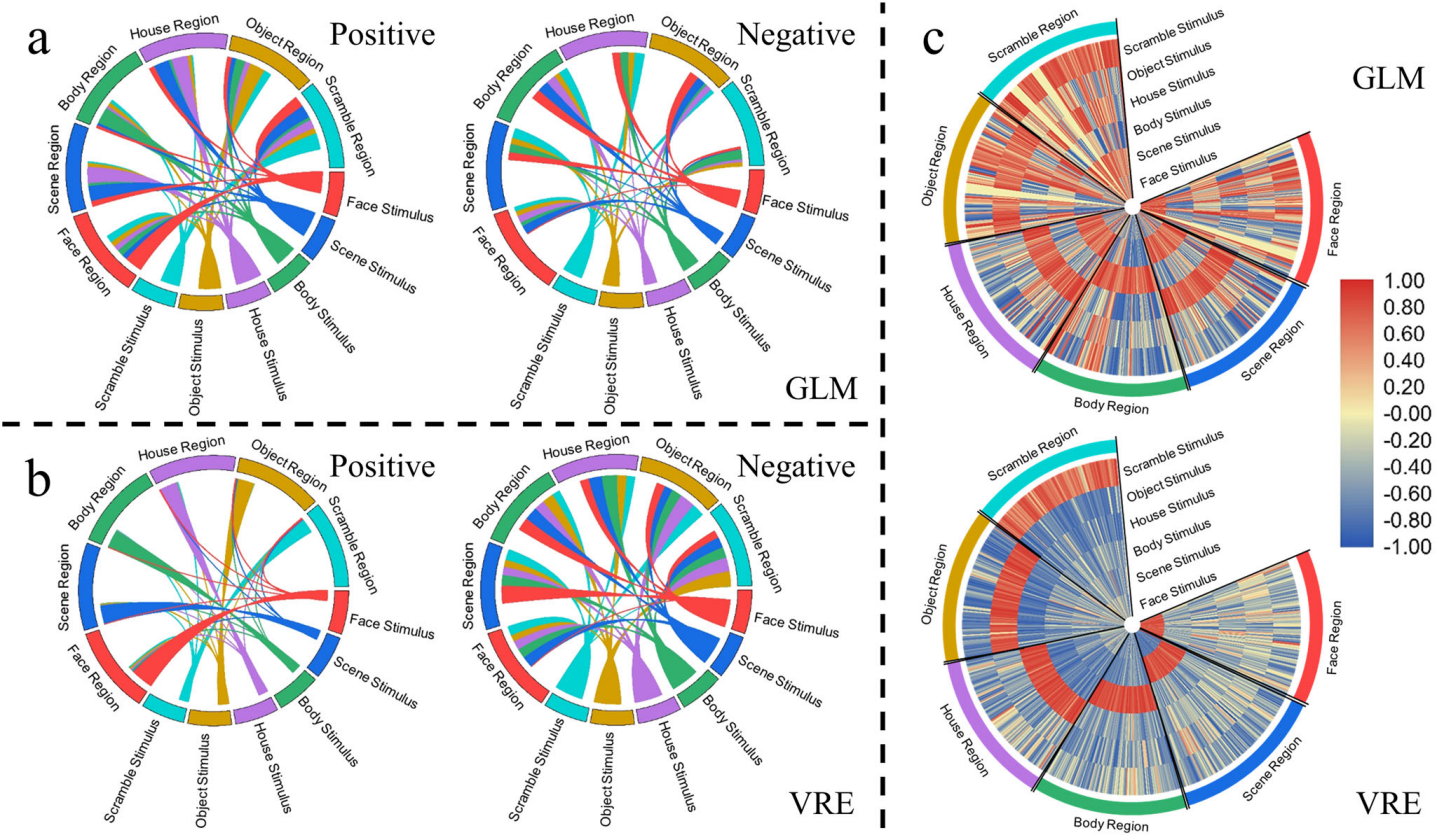
Pearson correlations between the classification indices (voxel-mean BOLD temporal sequences for the GLM and predicted label element sequences for the VRE) and classification labels (HRF-convolved stimulus temporal sequences for the GLM and stimulus temporal sequences for the VRE) for every stimulus block of higher-visual-areas localizer task. These results are analyzed from continuous volumes (the same 14 volumes shown in Fig. 2) obtained during one stimulus block for all 15 subjects. **a** Pearson correlations between the voxel-mean BOLD temporal sequences among the object-selective ROIs selected by the GLM and the HRF-convolved stimulus temporal sequences shown separately in positive and negative Circos plots. **b** Pearson correlations between the predicted label element sequences among the object-selective ROIs selected by the VRE and the stimulus temporal sequences are shown separately in positive and negative Circos plots. **c** Positive and negative Pearson correlations between the classification indices and classification labels are shown together in the heatmap for the GLM and VRE. The radial direction denotes the six stimulus conditions, and the circumferential direction signifies the ROIs selected by the two models.

To quantify the classification specificity of the GLM and VRE, we calculated Pearson correlations not only between the predicted label element sequences of the voxels selected by the VRE and the stimulus temporal sequences, but also between the averaged BOLD temporal sequences of the voxels selected by the GLM and the HRF-convolved stimulus temporal sequences. All blocks of all stimulus conditions (eight blocks of one condition for one subject) for all 15 subjects were used in this correlation analysis instead of the block-mean results of every condition shown in Fig. 2. Positive and negative correlation results are shown separately for GLM and VRE in Fig. 3a, b, respectively, and the line width in the Circos plot signifies the absolute value of the correlation between stimulus condition and ROI result. For a specific stimulus condition, a model with ideal classification accuracy needs to select the most informative features from the irrelevant alternatives. Put differently, the classification index (BOLD predicted label element sequence for VRE or voxel-mean BOLD temporal sequence for GLM) should be as positively correlated with this condition exclusively and as negatively correlated with other conditions as possible. Regarding the classification specificity of the models, the correlation results of the VRE model shown in Fig. 3b are in higher congruence with this norm than the results of the GLM shown in Fig. 3a. Moreover, the condition specificity differences between these two models are more prominent when both the positive and negative results are described in a single heatmap, as depicted in Fig. 3c. The radial direction of the heatmap denotes six stimulus conditions, and the circumference direction signifies the six ROIs selected by the two models, consisting of 720 columns (6 × 15 × 8, six ROIs, 15 subjects, and eight blocks of one condition for every subject; House and Scene are regarded as Place by original paper, so that the House sector and Scene sector in the GLM heatmap signify the same correlations; due to data leakage from the ROIs of some subjects from the original paper, four zero gaps exist in the Scramble, Object and Face sectors in the GLM heatmap).

**Fig. 4:**
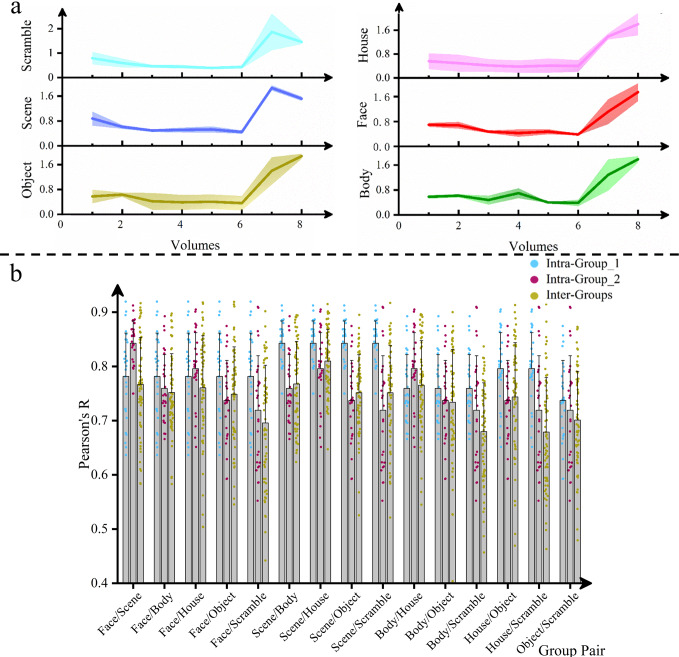
Dynamics of six stimulus-responsive regions and their Pearson’s correlation analysis results for Subject _01. **a** Dominating cognitive state dynamics temporally arranged from voxels’ RI summation of six stimulus-responsive regions are presented separately. The x-axis signifies the volume collection temporal sequence, with point 0 denoting the start of the stimulus block. The shaded area denotes the standard errors. **b** To estimate the difference between the cognitive state dynamics of distinct stimulus-responsive regions, inter-groups, and intragroup Pearson’s correlation analysis are performed between all stimulus group pairs. The correlation difference significances between inter and intra-groups are assessed by sign permutation test (${n}_{1}=28$, ${n}_{2}=64$ for intragroup and intergroup correlations correspondingly, *p* < 0.001, FDR-corrected significance level *p* < 0.05). No significant differences exist between the cognitive state dynamics of different stimulus-responsive regions.

Then we researched the difference between the dominating cognitive state dynamics of six object-selective regions during a stimulus block. As shown in Fig. 4a, the dynamics of the six object-selective regions for Subject_01 are similar. To quantify the difference between the dynamics of distinct object-selective regions, we performed intergroup and intragroup Pearson’s correlation analysis between all stimulus group pairs. Additionally, the sign permutation test (${n}_{1}=28$ and ${n}_{2}=64$ for intragroup and intergroup correlations, respectively, *p* < 0.001, FDR-corrected significance level *p* < 0.05) between the inter- and intragroup correlations demonstrated no significant differences between the dynamics of disparate object-selective regions. The individual cognitive state dynamics analysis results for the remaining 14 subjects are presented in Supplementary Figs. 15–28.

Compared with the conventional univariate GLM, we conclude that the VRE can achieve absolute classification specificity on the basis of rational ROI positioning. Cognitive state dynamic research indicates that different higher-visual stimuli are processed by different brain regions in similar working patterns.

### Memory retrieval after offline learning

Procedural memory is consolidated by “online” learning, in which skills initially improve relatively fast and more slowly thereafter^30,31^, and “offline learning”, which includes skill stabilization and improvement^31–33^. Offline learning expressed as delayed gains (DGs) is often dependent on sleep^34^. To reveal the brain response patterns to motor retrieval after offline learning, we applied VRE to fMRI data collected when 15 right-handed subjects performed four finger motion tasks (with one task trained beforehand) and obtained from ref. ^18^. The prior training and fMRI experiments were implemented over two consecutive days separated by an 18-h period (including 6 h of sleep). The subjects were required to perform four tasks during the fMRI experiment: trained hand performing the trained sequence (T_T), trained hand performing the untrained sequence (T_U), untrained hand performing the trained sequence (U_T), and untrained hand performing the untrained sequence (U_U). Among the four motion conditions, only the T_T condition was trained beforehand, whereas the other three conditions evolved from the T_T condition. Whether offline learning only occurs for the T_T condition or the four conditions during the delay (18-h period)? Memory consolidation in this experiment is expressed as DGs. Namely, “off-line” gains reflecting the procedural memory consolidation process are dependent on evolution occurring over a long period after practice^35,36^. Regarding motor sequences, DGs expressed as faster and more accurate performance are related to sleep^34,37^.

Posttraining and overnight behavior tests were conducted to grade the DG scores in motion performance speed and accuracy for each task. Based on the DG score, offline learning was demonstrated during the delay for the T_T condition. The speed gains and reduction in the absolute number of errors were significant (overnight test vs. posttraining test for T_T, in speed and accuracy correspondingly: *F*(1,14) = 14.42, *p* < 0.01; *F*(1,14) = 9.18, *p* < 0.01). Compared with the post training test score, no significant DGs were identified for other conditions. While estimating the DGs on the individual performance, however, the 15 subjects were divided into two groups, including the No_DG group, consisting of 5 subjects who had low improvements or reduced performance in speed and accuracy (<2%), and the DG group, consisting of 10 subjects who achieved valid gains in performance speed and accuracy.

**Fig. 5:**
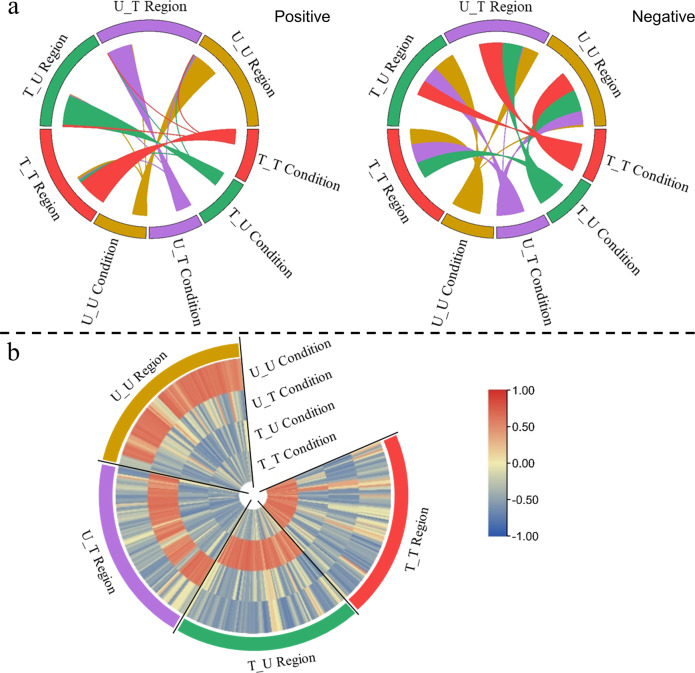
Pearson correlations between the predicted label element sequences and stimulus temporal sequences for every stimulus block of four motion tasks. These results are analyzed from continuous 14 volumes obtained during one block (2, 8, and 4 volumes were collected before, during and after the block) for all 15 subjects. **a** Pearson correlations between the predicted label element sequences among the stimulus-responsive ROIs selected by the VRE and the stimulus temporal sequences shown separately in positive and negative Circos plots. **b** Positive and negative Pearson correlations results shown together in the heatmap for all subjects. The radial direction denotes the four conditions, and the circumferential direction signifies the ROIs selected by VRE.

Given the particular experimental design, the data were preprocessed for every run. Then, we designed individual four-class classification models for the 15 subjects based on the VRE framework using 80% of fMRI data as the training dataset and the remaining 20% as the test dataset. Reasonable accuracy was achieved by the related models (99.1 $\pm$ 1.5%, average accuracy and standard deviation across all conditions for the 15 subjects), guaranteeing meaningful feature evaluation. We performed a similar Pearson correlation analysis as Fig. 3 for all blocks of all subjects, as shown in Fig. 5. Overall, 360 blocks (15 × 4 × 3 × 2, 15 subjects, four conditions, three runs per condition, two blocks per run) were adopted in the correlation analysis. Positive and negative results are separately shown in Fig. 5a, with the stimulus sequence positively correlating with the predicted label element sequence from the corresponding stimulus-responsive region and negatively correlating with the predicted label element sequences from other stimulus-responsive regions; positive and negative results are shown together in Fig. 5b.

After VRE feature evaluation, we found that the selected voxels clustered in five regions: the dorsal lateral prefrontal cortex (DLPFC), bilateral sensorimotor area, medial temporal lobe (MTL), and vision association area. Then, we analyzed the dominating cognitive state dynamics of the five clusters across four tasks.

**Fig. 6:**
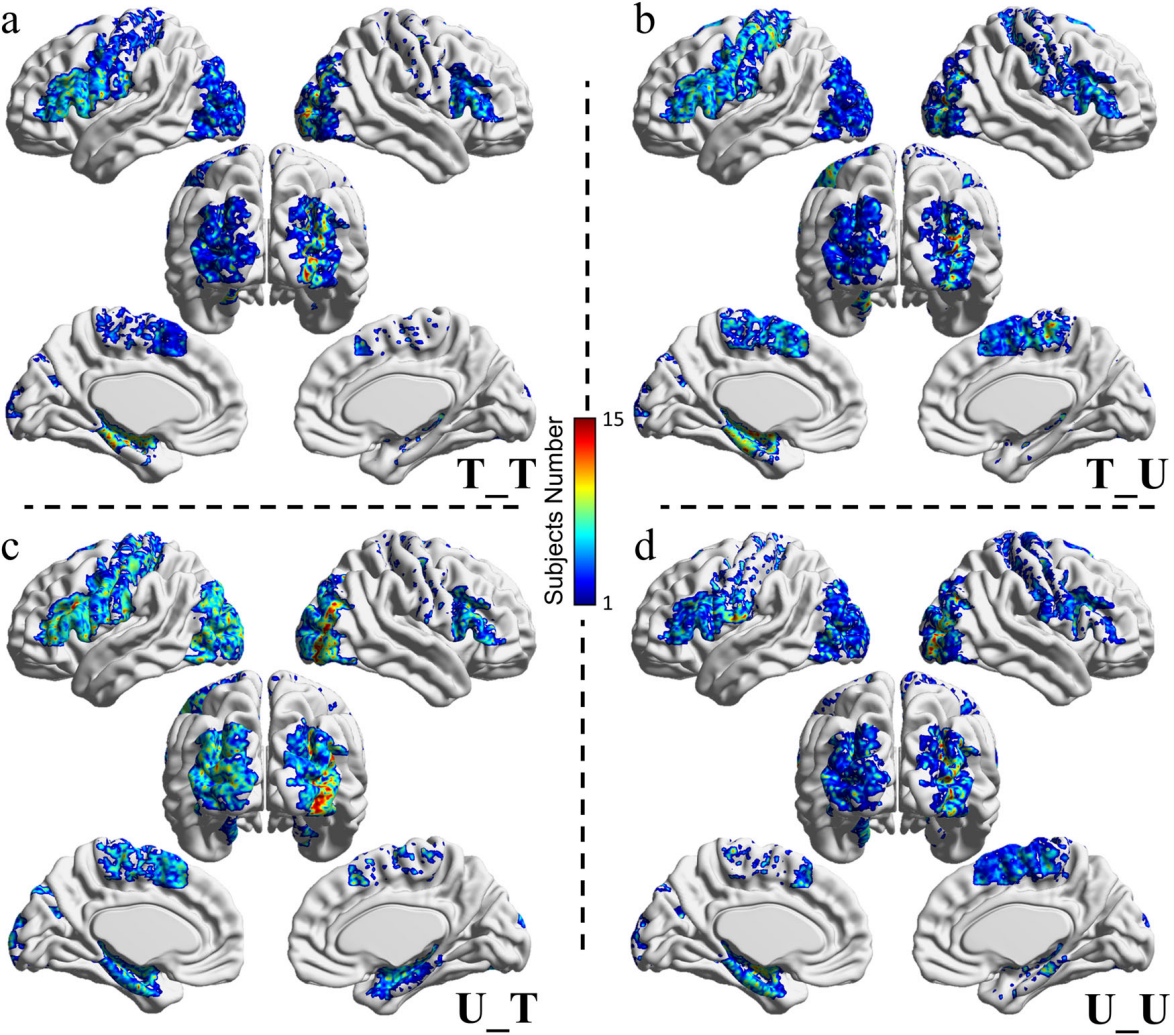
MNI projected topologies in lateral and posterior views of the five clusters with dominating tendencies across the 15 subjects for four memory retrieval tasks. The red-colored voxels (maximum 15 on the color bar) denote that these voxels were selected for all subjects. **a** MNI projected topology for T_T condition. **b** MNI projected topology for T_U condition. **c** MNI projected topology for U_T condition. **d** MNI projected topology for U_U condition.

The five clusters with dominating tendencies were projected into MNI space and overlapped across the 15 subjects, as shown in Fig. 6a–d for the T_T, T_U, U_T, and U_U conditions, respectively. The magnitude was affected by both the spatial variability across brains and the subjective cognition differences to a certain condition. Regarding the T_T condition, the voxels with dominating dynamics were significantly clustered across the left DLPFC, left sensorimotor area, right precentral gyrus, left MTL, and right vision association area for all subjects. For the T_U condition, the voxels with dominating dynamics were clustered similarly to but not exactly as those of the T_T condition, which indicates that the cognitive states related to these two conditions are similar to a certain extent. In the U_T condition, the voxels with dominating dynamics were significantly clustered across the left DLPFC, left the sensorimotor area, bilateral MTL, and bilateral vision association area for all subjects. In the U_U condition, the voxels with dominating dynamics were significantly clustered across the right DLPFC, left postcentral gyrus, right sensorimotor area, bilateral MTL, and right vision association area for all subjects.

**Fig. 7:**
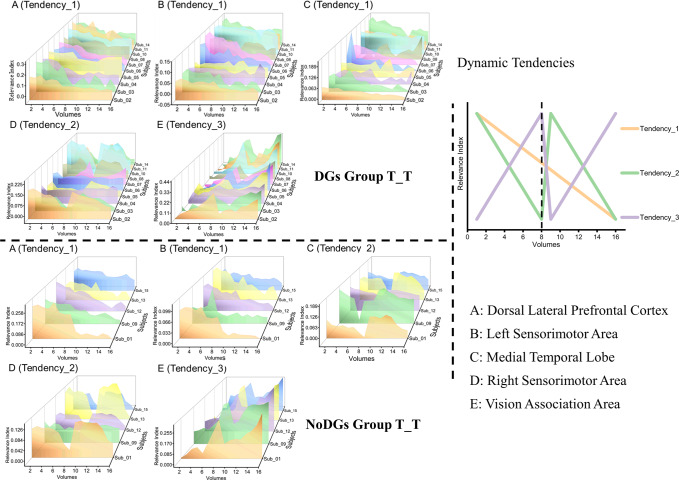
Three dynamic tendencies (upper-right plots; the dashed line denotes the boundary for two continuous blocks of one run) and dynamics plots of the five clusters across subjects of the DGs group (upper-left plots) and the NoDGs group (bottom-left plots) for the T_T condition. The three axes in the waterfall plots are defined as follows: Volume 0 on the $x$ axis denotes the motion performance run onset, the first eight volumes were collected during the first block, and the second eight volumes were collected during the second block; the $y$ axis signifies the relevance index (RI); and the points on the $z$ axis denote the subject IDs, indicating that the result was analyzed by an individual subject’s VRE model.

Based on the dominating dynamic of every cluster, cognitive state dynamics within three continuous runs for one experimental condition were averaged as the final dynamic for this condition. As noted in the upper-right portion of Fig. 7, the dominating dynamics of the five clusters are consistent with three tendencies in displaying certain patterns of variation in classification contributions.

The results for the DG group and No_DG group are shown separately in Fig. 7. For the DG subjects in the T_T condition (depicted in the upper-left part in Fig. 7), the dynamics of the DLPFC, the sensorimotor area ipsilateral to the performance hand and the MTL denote decreasing classification specificities for these three clusters during the whole run. In addition, the dynamic of the sensorimotor area contralateral to the performance hand indicates a decreasing classification contribution of this cluster during every block, and the dynamic of the vision association area signifies an increasing classification contribution of this cluster during every block. For the No_DG subjects in the T_T condition (shown in the bottom-left part of Fig. 7), the dynamics of the four clusters are similar to those of the DGs group, except for the dynamic of MTL, which indicates a decreasing classification contribution of this cluster during every block.

**Fig. 8:**
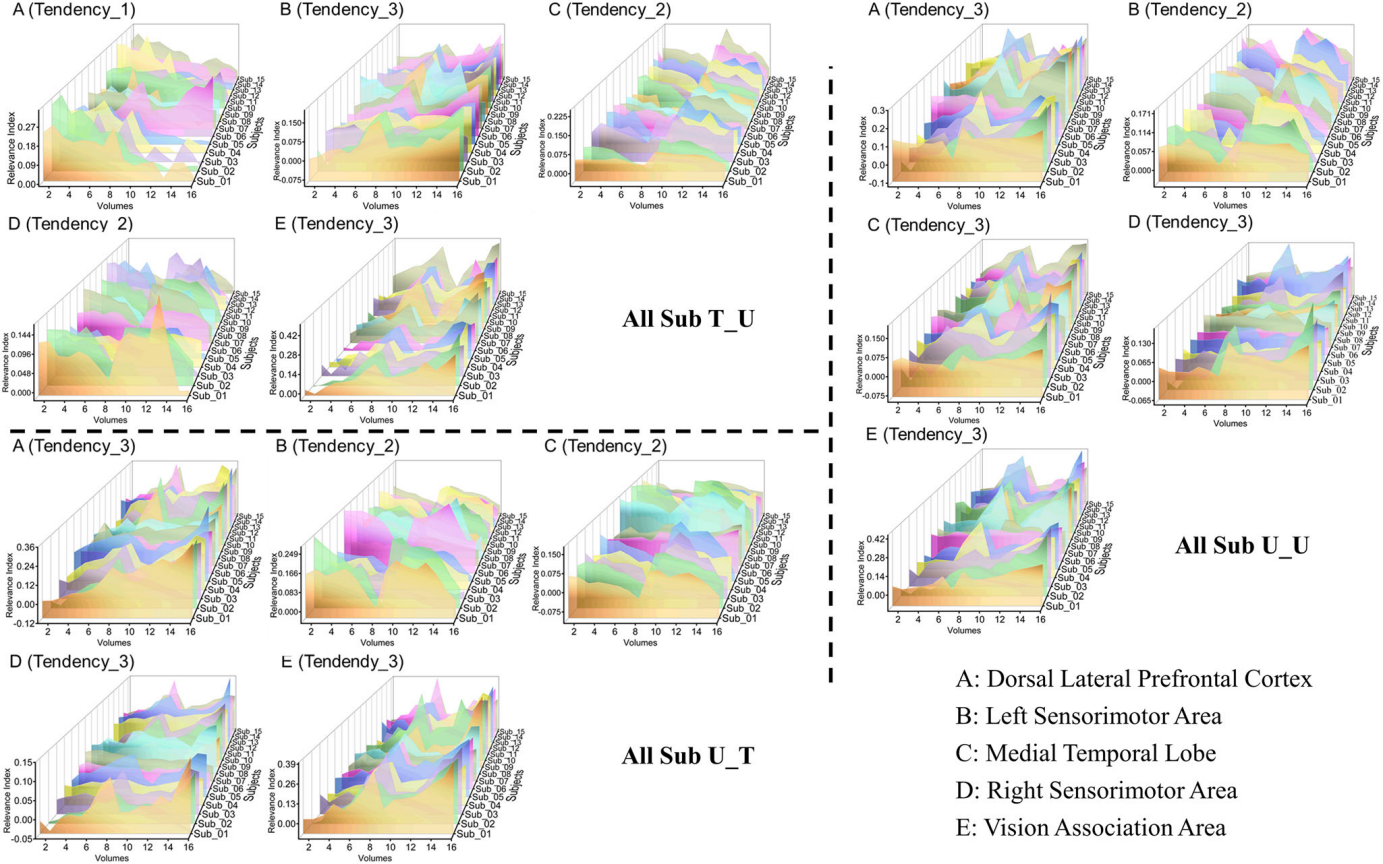
Dynamics plots of five clusters across all subjects for the T_U (upper-left plots), U_T (bottom-left plots), and U_U conditions (upper-right plots). The three axes in the waterfall plots are defined as follows: Volume 0 on the $x$ axis denotes the motion performance run onset, the first eight volumes were collected during the first block, and the second eight volumes were collected during the second block; the $y$ axis signifies the relevance index (RI); and the points on the $z$ axis signify the subject IDs, indicating that the result was analyzed from an individual subject’s VRE model.

Given that the difference in the dynamics of the five clusters between the DG group and the No_DG group was not significant as that under the T_T condition, the dynamics were not analyzed separately for the other three experimental conditions. For the T_U condition, as noted in the upper-left part of Fig. 8, the dynamics of the DLPFC denote a decreasing classification contribution from this cluster during the whole run, which is similar to that noted in the T_T condition. In addition, the dynamics of the sensorimotor area ipsilateral to the performance hand and the vision association area indicate increasing classification specificities from these two clusters during every block, and the dynamics of the MTL and sensorimotor area contralateral to the performance hand signify decreasing classification specificities from these two clusters during every block.

For the U_T condition, as depicted in the bottom-left part of Fig. 8, the dynamics of the DLPFC, the sensorimotor area ipsilateral to the performance hand and the vision association area indicate increasing classification specificities of these three clusters during every block. In addition, the dynamics of the sensorimotor area contralateral to the performance hand and the MTL denote decreasing classification specificities from these two clusters during every block.

The upper-right part of Fig. 8 shows the dynamics of the five clusters under the U_U condition. Similar to the U_T condition, the dynamics of the DLPFC, the sensorimotor area ipsilateral to the performance hand and the vision association area indicate increasing classification contributions of these three clusters during every block. Moreover, the dynamics of the sensorimotor area contralateral to the performance hand denote a decreasing classification contribution of this cluster during every block. Unlike the U_T experimental condition, however, the dynamics of the MTL indicate an increasing classification contribution from this cluster during every block.

Collectively, the region positions for the four disparate tasks were similar but functioned under different patterns. Explicitly, we found three dominating dynamics across five regions for the four different conditions with high reproducibility across the subjects. The individual cognitive state dynamics within the five clusters of the 15 subjects are presented in Supplementary Figs. 29–43. Pearson correlation analysis between the cognitive state dynamics in the runs and the three dominating tendencies across the four tasks for all 15 subjects is provided in Supplementary Figs. 44, 45.

## Discussion

To link brain “representation” and “operation” through dynamic interactions at the microscale, we designed a variational Bayesian-based feature evaluation strategy, named variational relevance evaluation (VRE), to assess the validity of brainwide features based on individual fMRI datasets. By comparing the predicted label element computed from one feature for the correct stimulus condition and other alternatives, we proposed the RI to evaluate the contribution of every feature to the correct classification. VRE allows analysis of the whole-brain as an entirety without preliminary feature preselection and achieves reasonable linear classification accuracy. Applying two fMRI datasets, we demonstrated the following: (1) by eliminating unrelated features across model training, the VRE prevents overfitting and achieves better classification accuracy than a GLM; (2) the selected voxel distribution is consistent with known functional anatomy; (3) six higher-visual object-selective brain regions function in similar working patterns; (4) the brain representations for four memory retrieval tasks after offline learning spread in similar areas; and (5) three cognitive state dynamics in five disparate regions across four memory retrieval tasks after offline learning are identified by VRE.

In regard to the problem of how the brainwide cortical circuits are linked at the microscale to advance cognition functioning for individuals, the voxel dependency of multivariate pattern analysis (MVPA) makes it an ideal choice. Besides, advances in machine learning have provided enough choice for MVPA application in the exploration of neural mechanisms behind detectable brain activity. However, the application of machine learning always requires big data to help brighten the model, which is not accessible or not easy for neuroimaging condition. Given the concerns of overfitting or model impairment due to the high-dimensionality and minor-dataset characteristics of the fMRI data or the possible noise inherent in a vast number of voxels, especially the research based on the individual dataset, a feature selection strategy is needed to perform dimension reduction beforehand^2^. Traditional feature selection methods include the recruitment of anatomical landmarks, functional localizers, or a univariate statistical analysis performed with training data or data from an additional experiment^22,23,38,39^. Another option is a transformation of the original feature space into a subspace of fewer dimensions using principal component analysis (PCA) or independent component analysis (ICA)^19^. The Searchlight method^4^ takes advantage of the correlation structure within a 5-mm radius sphere of voxels. Sparse logistic regression (SLR) achieves feature selection during the linear regression process with the help of automatic relevance determination (ARD), but the high computational costs (time and memory) restrict its use on a large spatial scale. These methods, however, neglect the dependency among voxels or do not take into account potential correlations between spatially remote voxels. Given the situation of high-level cognition with poor availability to predefine regions of interest (ROIs) and the potential involvement of multiple distributed brain areas, an approach based on the minor dataset for characterizing both region correlation and contribution diversity across the entire brain is needed. VRE is developed to characterize both region correlation and contribution diversity across the whole-brain voxels based on an individual dataset that are collected via conventional paradigms.

Using high-level visual stimulus localizer data, we compared the classification accuracy and selected voxel position rationality of VRE with those of the GLM. The individual results shown in Fig. 2 indicate that the predicted label element sequences of VRE are significantly consistent with the corresponding stimulus temporal sequences compared with the relevance between the voxel-mean BOLD responses of GLM and the HRF-convolved temporal sequences. Moreover, pairing all quantified correlations between these two classification indices and their “labels” shown in Fig. 3, we conclude that the VRE outperforms the GLM in selecting stimulus-specific voxels. For individual topology mapping, the distribution of voxels selected by the VRE is consistent with the known functional anatomy;^29^ however, some position differences are noted between the results of the two models, which can possibly be explained by the benefit of voxels with noise correlation^38^. We found no significant difference between cognitive state dynamics of different object-selective ROIs, demonstrating higher-visual stimulus unfolding in distinct brain regions with similar working patterns.

Then we implemented the VRE on an individual fMRI dataset across four memory retrieval tasks to reveal the brain responses after offline learning. The classification accuracy evaluation (Fig. 5) demonstrated the feature validity selected by VRE. Therefore, every volume was valid for separation^21,23^. The RI dynamics for each voxel can be obtained with the same temporal resolution as the BOLD signal. By summing the RIs of the voxels compatible with the dominating tendency, the dominating dynamic of every cluster was obtained. The voxels compatible with the dynamics clustered in five similar regions for the four conditions, as shown in Fig. 6. These results reveal that a representation was established for a specific task as a function of learning^40,41^ and attentional demands^42,43^. Three dominating dynamics were identified for the five clusters across the four conditions, as depicted in Figs. 7,8. Subjects were required to perform the tasks in a block paradigm, with two blocks comprising a run. The three dominating tendencies that emerged included sustaining suppression during the entire run, suppression during every block, and enhancement during every block. The DLPFC is associated with the control of spatial attention, action intention, and object-order memory^44–46^, and is activated during the assimilation of new memories with the existing memory structure^47^. According to our results, the spatial attention needed to perform the task decreased as the task was performed iteratively due to the familiarity with the T_T and T_U conditions. The DLPFC RI increased during every block to aid in new memory assimilation if the motion sequence was not so familiar, such as that noted in the U_T and U_U sequences. Regarding the MTL, which is associated with object-order memory^44,48,49^, the activity can be modulated by the number of details of memory content^50–52^. As our results show, MTL dynamics varied among the three tendencies, which might reflect the response patterns under different tasks with distinct familiarity. For sensorimotor areas, repetition-driven reduction in neural activity in M1 is related to motion sequence learning^53–55^ and enhanced physiological signal patterns in response to repeated experience may be related to sequence-specific procedural memory consolidation processes^56^. The dominating dynamics of the ipsilateral sensorimotor area to the performance hand was opposite that of the contralateral sensorimotor area. These results indicate that the ipsilateral sensorimotor area might be related comparatively to procedural memory retrieval, whereas the contralateral sensorimotor area might be comparatively associated with motion execution. Intriguingly, the sensorimotor ROI ipsilateral to the performance hand selected by VRE is contradictory to known functional anatomy. A study found that anisomycin injection disrupted the performance of internally generated sequential movements by interfering with the information storage in M1^57,58^. As previous studies have shown, posterior neocortical components in conjunction with the posterior hippocampus (pHPC) determine the local, spatio-perceptual aspects of the experience^59^. Our results identify possible working patterns of related visual regions related to motion performance after offline learning. The MTL responded differently during the T_T condition in subjects from the DG and No_DG groups. This result may reveal brain response characteristics during the retrieval tasks with different familiarities with existing memory content for individuals with distinct offline learning abilities.

In summary, some cognitive functions express themselves in different brain representations with similar operations, such as the higher-visual areas localizer task. In contrast, other cognitive functions manifest themselves in similar brain regions with distinct operations, such as memory retrieval tasks after offline learning. Identifying the stimulus-responsive regions and the working patterns by assessing the classification-contribution dynamics of brainwide features is the main focus of this research. Using the batch-feature-input algorithm based on the variational Bayesian (VB) framework, VRE prevents overfitting based on individual datasets. Nevertheless, the application of hierarchical clustering to identify the dominating dynamics in a selected voxel cluster is a coarse-scale process, and these dominating dynamics may be segmented into more elaborating tendencies, which we plan to investigate in the future. We believe that the voxels that are incompatible with the dominating trend of their cluster cannot be simply regarded as noise. Consequently, we plan to design another postprocessing strategy to explain the neuroanatomical organization of cognitive function on more fine-grained biological scales, such as between subregions of the hippocampus.

For high temporal resolution in pattern analysis, we can apply the VRE to fMRI and electroencephalography (EEG), magnetoencephalography (MEG), or near-infrared spectroscopy (NIRS) data concurrently to mine more high spatiotemporal-resolution details related to various cognitive functions, which might provide new indicators for medical diagnoses, particularly for high-level cognition-related diseases. Moreover, the VRE can be applied to construct a customized medical therapeutic model based on individual data due to the controllable voxel quantity analyzed simultaneously, by substituting the linear algorithm in the classification model proposed in this paper with other sophisticated machine learning algorithms.

## Methods

### Localizer for higher-visual areas

To localize the higher-visual object-selective regions, the experiment gathering 3 Tesla fMRI data was implemented when the 15 subjects (mean age 29.4 years, 6 females) viewed 144 pictures from six different categories: human faces, human bodies without heads, small objects, houses, outdoor scenes, and phase scrambled images. The subjects were required to perform four block-design runs, each with two 16 s blocks per category (eight volumes were collected during one block). The two blocks of one category were presented separately in a run, with 16 unique images presented (16 images were presented for 900 ms each, separated by an interval of 100 ms) in each block. For details, please refer to ref. ^17^. A total of 384 fMRI volumes from 48 blocks for every subject were divided into a training data matrix (80%) and a test data matrix (20%).

### Memory retrieval after offline learning

The objective of this experiment was to investigate brain responses related to memory retrieval after offline learning. Fifteen healthy subjects (mean age ± std = 25.7 ± 4.4 years, five females) participated in this experiment. All experiments were implemented on 2 consecutive days with a block paradigm. The subjects were trained with a particular five-element finger sequence the day prior to the fMRI experiment with their nondominant (left) hand, and performed the same sequence and the mirror-reversed sequence with their left and right hands afterward while undergoing an fMRI scan.

The subjects needed to perform the task with three continuous runs consisting of two blocks (eight volumes were collected, and the motion sequence was repeated eight times during one block), each on one condition in the fMRI experiment. Specifically, the subjects performed four tasks, including the trained hand performing the trained sequence (T_T), the trained hand performing the untrained sequence (T_U), the untrained hand performing the trained sequence (U_T) and the untrained hand performing the untrained sequence (U_U), with an identical auditory-paced performance rate after an 18-h posttraining break (including at least 6 h of proper sleep). Only the T_T condition was trained on the first day.

Three tests to estimate the delayed gains (DGs) in speed and accuracy were performed during the two consecutive days: pretraining test, posttraining test, and overnight test after scanning. The subjects were required to perform the specified motion sequence during the test block as fast and as accurately as possible. Compared with the pretraining test score, the posttraining gains and overnight DGs of every subject were quantified as a percentage (pretraining = 100%). The DGs are defined as the performance improvement in speed and accuracy expressed by the overnight test compared with the posttraining test score. By comparing the overnight test score with the posttraining test score, significant offline learning occurred during the 18-hour posttraining break for the T_T condition. See ref. ^18^ for details.

To prevent the reconsolidation from possibly occurring during the scanning period, an additional 15 healthy subjects in the control group were required to learn the same finger sequence on the first day and conduct the same behavior tests as the fMRI group. Compared with the overnight test score of the control group, no significant improvement was observed for the fMRI group. In sum, no reconsolidation occurred during the scanning period.

### VRE algorithm framework

**Fig. 9:**
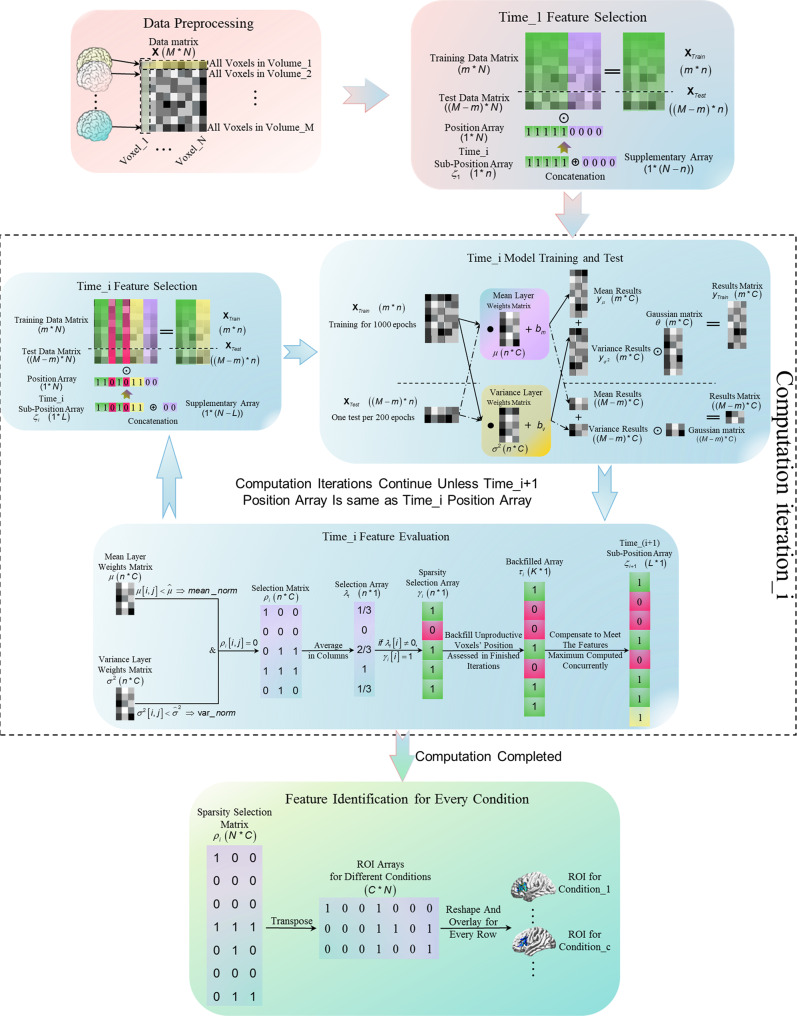
VRE analysis procedures. Data Preprocessing (orange background), Feature Selection of the first computation iteration (orange to blue gradient background), and computation iterations consisting of three parts (blue background, Feature Selection, Model Training and Test, and Feature Evaluation) to evaluate the whole-brain features’ validity one by one, and Feature Identification for Every Condition (the green part, performed when the Position Array of two consecutive computation iterations are same as each other). $\odot$ signifies the Hadamard product. $\oplus$ signifies the concatenation operation. $\bullet$ denotes the matrix product.

In this section, we first introduce the structure of the VRE, as shown in Fig. 9. Here, the orange part is performed only once at the beginning, Feature Selection (in orange to blue gradient background) of the first computation iteration is implemented once at the beginning, three parts in blue constituting one computation iteration (iteration, in short) denoted in Fig. 1a are conducted iteratively, and the green part signifying the stimulus-responsive regions extraction after the last computation iteration is finally performed. To prevent probable overfitting due to high-dimensionality and minor-dataset characteristics of the fMRI data, we develop the batch-feature-input trick to reduce the dimensionality in a single computation iteration.

**Fig. 10:**
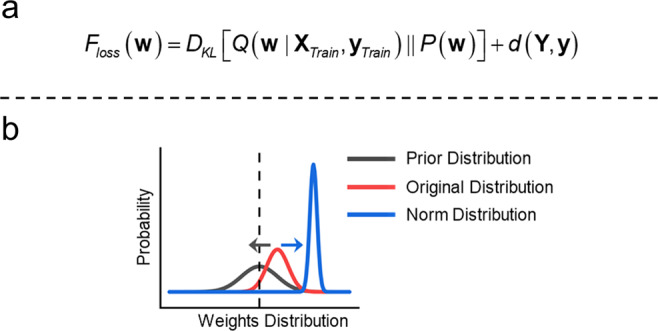
Two key adversarial strategies. **a** The first adversarial strategy is manifested in the loss function, in which the first part on the right-hand side is the Kullback–Leibler divergence between the constructed posterior distribution $Q({{{{{\bf{w}}}}}}|{{{{{{\bf{X}}}}}}}_{{{{{{\mathrm{Train}}}}}}},{{{{{{\bf{y}}}}}}}_{{{{{{\mathrm{Train}}}}}}})$ and the noninformative prior distribution$P({{{{{\bf{w}}}}}})$, and the other part on the right-hand side is a distance (the Euclidean distance in our research) between the predicted labels $y$ and true labels $Y$. **b** The second adversarial strategy with the original (default) distribution (red line) for every weight to signify the constructed posterior distribution which is encouraged to be regularized to a prior distribution (black line) across model training, adversarial to the direction denoted by norm distribution (blue line) applied to evaluate the feature validity after model convergence.

Our objective is to linearly relate the BOLD responses to the stimulus conditions with the least number of informative features. Among the features of the whole-brain, we need to distinguish informative features from other alternatives. When constructing the linear classification model in every computation iteration, we assume an intrinsic distribution function for every weight related to a specific feature-stimulus condition. Thus, reasonable classification accuracy can be achieved as long as the weights are fitted in their respective distribution functions. Obtaining the exact weight distribution functions for every weight is an extremely intractable problem; however, we can determine a series of methods to approximate the distribution functions for every weight. Stochastic approximations, such as the Markov chain Monte Carlo (MCMC) method, are theoretically suitable for this problem but impractical due to the high computational resource consumption for iteratively inferring each weight. Thus, we propose a VRE strategy based on the variational Bayesian (VB) framework, which is a deterministic approximation algorithm. Regarding algorithm practice, two parallel linear layers are arranged in the classification model to approximate the parameters of the intrinsic distribution function for the weights mentioned above (Gaussian distribution in this research, mean and variance parameters need to be approximated from the two layers). By the application of the two parallel layers, we designed two adversarial strategies indicated in Fig. 10. Specifically, one strategy involved the loss function, and another strategy was implemented in the Feature Evaluation stage. As a result of the two adversarial strategies, the features that are poorly relevant to the specific condition can be distinguished and are not calculated in the following iterations by setting the corresponding Selection Array elements of these features to zero.

It should be noted that iteration and epoch are two different concepts in this paper. An intact computation iteration is composed of three stages: Feature Selection, Model Training and Test, and Feature Evaluation. In addition, the model needs to be trained for at least 1000 epochs. We need to adjust the three sets of distribution parameters shown in Fig. 10b to guarantee the model convergence with the least number of informative features in every computation iteration. In the following section, all parts of the VRE algorithm framework are introduced sequentially, as depicted in Fig. 9.

### Data preprocessing

First, we should clarify that data from different runs are not preprocessed simultaneously in the case of probable cross-contamination. The Statistical Parametric Mapping 12 (SPM12, Wellcome Department of Imaging Neuroscience, University College London, London, UK) software package with MATLAB (MathWorks, Natick, Ma) was employed for data preprocessing. Among a series of data from one run, the functional data are first realigned to the first functional volume to assess the effect of head motion and then coregistered to T1-weighted high-resolution anatomical images. During the normalization stage, the coregistered volumes are interpolated into a new volume consisting of 3 × 3 × 3 mm^3^ voxels to match the standard T1 template image defined by the Montreal National Institute (MNI).

After SPM processing, the data need to be further processed to eliminate the baseline signal. First, according to the temporal sequence obtained across the experiment, the volumes collected during the resting stage are averaged and built to serve the baseline signal of the corresponding run. Second, the baseline signal is subtracted from every volume collected during the stimulus period to minimize the probable effects of noise. Finally, the overall voxels from a single volume are normalized to the standard Gaussian distribution. Furthermore, all normalized matrices are reshaped into data arrays and then stacked into a matrix with M rows (for M volumes) and N columns (N voxels per volume), as shown in the data preprocessing stage of Fig. 9.

The goal of the preprocessing step is to verify the feasibility and enhance the resolution of the data, whereas that of the postprocessing stage is to eliminate the interference from noise and enable preliminary normalization of the features. Finally, we reshaped the neuroimaging data from matrices to arrays because we only wanted to study the correlation among voxels or cortical regions (voxel clusters) instead of the spatial relationship among cortical regions and the temporal relationship among time points. In sum, we transform all fMRI data for an individual to a matrix with the Num_i row representing the N features of all dimensions collected from the corresponding volume and the Num_j column representing the different M Num_j features from M different volumes.

### Feature selection

The three stages of an intact computation iteration are introduced in the following three parts: Feature Selection, Model Training and Test, and Feature Evaluation. In the Feature Selection stage, the features awaiting the validity evaluation need to be selected from the overall dimension. Before a more explicit introduction, we need to clarify two dimensions, the overall dimension comprised of all features in the whole-brain and the local dimension comprised of the features analyzed in the next computation iteration. The Position Array is proposed to locate the positions of features across the overall dimension.

To prevent overfitting, we restricted the feature quantity in one iteration by the application of a batch-feature-input trick, as shown in Fig. 1a. Given the computational efficiency, we set a maximum number of features to be computed concurrently in one computation iteration (max_fea), which is the default feature quantity if the number of features not computed previously is sufficient for compensation.

As an important part of the position array, the Sub-Position Array estimated in previous iterations with sparsity is composed of “one” elements and “zero” elements. Here, “one” elements denote both the informative feature positions assessed through the completed iterations (the green elements depicted in Fig. 9) and those needed to compensate for max_fea (the yellow elements depicted in Fig. 9) to signify the positions of features computed for the next iteration. Correspondingly, “zero” elements consist of the positions of unproductive features assessed through the completed iterations (the magenta elements depicted in Fig. 9).

Accordingly, the Position Array of Time_i Feature Selection is composed of the Sub-Position Array ${\zeta }_{i}$ evaluated in Time_(i-1) Feature Evaluation designed to locate the positions of features across local dimension; and the concatenated zeros part, the Supplementary Array (where $\oplus$ signifies the concatenation operation in Fig. 9), the elements of which denote the features awaiting computation in future iterations (the purple elements in Fig. 9). Here, the local dimension of the Sub-Position Array can be transformed into the overall dimension of Position Array.

Regarding the Time_1 Feature Selection depicted in the orange to blue gradient background of Fig. 9, the Position Array is composed of the Sub-Position Array ${\zeta }_{1}$ with “one” elements in shape [1*n] denoting the default features quantity equaling to max_fea and the Supplementary Array with “zero” elements in shape [1*(N-n)].

After construction of the Position Array, the next step is to divide the data matrix obtained from the Data Preprocessing stage into two data matrices according to a specified proportion (80/20% in this paper) with no overlap. The major matrix (Training Data Matrix) is used for model training, and the minor matrix (Test Data Matrix) is used for model generality testing. Then, the feature batches used in the next iteration are the Hadamard products of these two data matrices and the Position Array mentioned above ($\odot$ signifies the Hadamard product). As Fig. 9 depicts, the features needed in the next iteration are composed of the productive features assessed through the completed iterations and the complementary features compensated to meet max_fea.

### Model training and test

In this part, the second stage of an intact computation iteration, Model Training and Test, is introduced. Unlike conventional feature selection models, VRE is required to construct multiple models to match the batch-feature-input trick. As a result of the variable dimension for the feature batch selected by the Feature Selection stage (the number of features $n$ computed simultaneously is either equal to max_fea when the number of features not computed previously is sufficient for compensation or the number of informative features assessed across the finished iterations), a customized new model with two parallel linear classification layers is established to match the dimension. Feature Evaluation, which is performed in the last stage of an iteration, is regarded as valid only if the model can achieve convergence (above-chance classification accuracy is obtained). To guarantee convergence, every model needs to be trained for at least 1000 epochs and tested every 200 epochs; the training epoch increases by 1000 epochs until the maximum (maximum epoch = 3000) if convergence is not achieved after the convergence evaluation implemented every 1000 epochs. Otherwise, the iteration is suspended, and the feature quantity is increased for the next iteration if the model is not convergent after 3000 epochs, given that the informative features are not sufficient for convergence. The first adversarial strategy expressed by the two parallel linear layers is introduced from the theoretical and algorithmic directions as described below.

After Feature Selection, features with *n* dimensions are selected from the Data Matrix for the Time_i Model Training and Test stage, as shown in Fig. 9. The Training Data Matrix, ${{{{{{\bf{X}}}}}}}_{{{{{{\mathrm{Train}}}}}}}$ with dimensions $m*n$, the rows of which signify the $n$-dimensional feature vectors selected from one volume, and ${{{{{{\bf{Y}}}}}}}_{{{{{{\mathrm{Train}}}}}}}$ with dimensions $m*C$, is the corresponding $C$-dimensional ($C$ categories in sum) one-hot true labels for the $m$ training vectors. Correspondingly, ${{{{{{\bf{X}}}}}}}_{{{{{{\mathrm{Test}}}}}}}$ is the Test Data Matrix with dimensions $(M-m)*n$, and ${{{{{{\bf{Y}}}}}}}_{{{{{{\mathrm{Test}}}}}}}$ is the corresponding label matrix with dimensions $(M-m)*C$. As shown under the dashed line in the Model Training and Test stage of Fig. 9, test data are manipulated to assess the convergence of the model after 200 training epochs.

1 $${{{{{\bf{y}}}}}}={{{{{\bf{X}}}}}}\cdot {{{{{\bf{w}}}}}}+b\,$$

To establish a linear classifier, a fully connected layer ($\cdot$ denotes the matrix product, ${{{{{\bf{w}}}}}}$ and $b$ are the weight matrix and bias respectively, in Eq. (1)) between input data ${{{{{\bf{X}}}}}}$ (${{{{{{\bf{X}}}}}}}_{{{{{{\mathrm{Train}}}}}}}$ or ${{{{{{\bf{X}}}}}}}_{{{{{{\mathrm{Test}}}}}}}$) and predicted label ${{{{{\bf{y}}}}}}$ (${{{{{{\bf{y}}}}}}}_{{{{{{{{\mathrm{Train}}}}}}}}}$ or ${{{{{{\bf{y}}}}}}}_{{{{{{\mathrm{Test}}}}}}}$) are constructed. The native Bayesian approach assumes variable continuity and independence of the approximated model. Given $m$ training samples and labels$\{({{{{{{\bf{X}}}}}}}_{1},{{{{{{\bf{Y}}}}}}}_{1}),\cdots ,({{{{{{\bf{X}}}}}}}_{m},{{{{{{\bf{Y}}}}}}}_{m})\}$ in our model, the true joint posterior distribution for the weight vector,${{{{{{\bf{w}}}}}}}_{{{{{{\mathrm{True}}}}}}}$, is expressed in Eq. (2).

2 $$P({{{{{{\bf{w}}}}}}}_{{{{{{\mathrm{True}}}}}}}|{{{{{{\bf{X}}}}}}}_{{{{{{\mathrm{Train}}}}}}},{{{{{{\bf{Y}}}}}}}_{{{{{{\mathrm{Train}}}}}}})=\mathop{\prod }\limits_{i=1}^{c}\mathop{\prod }\limits_{j=1}^{n}p\left({\omega }_{True\,j}^{(i)}|{{{{{{\bf{X}}}}}}}_{{{{{{\mathrm{Train}}}}}}},{{{{{{\bf{Y}}}}}}}_{{{{{{\mathrm{Train}}}}}}}\right)$$

3 $$Q({{{{{\bf{w}}}}}}|{{{{{{\bf{X}}}}}}}_{{{{{{\mathrm{Train}}}}}}},{{{{{{\bf{y}}}}}}}_{{{{{{\mathrm{Train}}}}}}})=\mathop{\prod }\limits_{i=1}^{c}\mathop{\prod }\limits_{j=1}^{n}q\left({\omega }_{j}^{(i)}|{{{{{{\bf{X}}}}}}}_{{{{{{\mathrm{Train}}}}}}},{{{{{{\bf{y}}}}}}}_{{{{{{\mathrm{Train}}}}}}}\right)$$

Nonetheless, the solution of the true joint posterior probability$P({{{{{{\bf{w}}}}}}}_{{{{{{\mathrm{True}}}}}}}|{{{{{{\bf{X}}}}}}}_{{{{{{\mathrm{Train}}}}}}},{{{{{{\bf{Y}}}}}}}_{{{{{{\mathrm{Train}}}}}}})$ is extremely intractable. To approximate the true joint posterior probability expressed in Eq.(2), the VB approach assumes a constructed posterior distribution $Q({{{{{\bf{w}}}}}}|{{{{{{\bf{X}}}}}}}_{{{{{{\mathrm{Train}}}}}}},{{{{{{\bf{y}}}}}}}_{{{{{{\mathrm{Train}}}}}}})$, as depicted in Eq. (3), where ${{{{{{\bf{y}}}}}}}_{Train}$ is the predicted labels for training data${{{{{{\bf{X}}}}}}}_{{{{{{\mathrm{Train}}}}}}}$. Equation (4) is the Bayesian formula in our model. Equation (5) is the centered isotropic multivariate normal joint Gaussian prior distribution of ${{{{{\bf{w}}}}}}$, the noninformative prior distribution of which is a Gaussian distribution with two hyperparameters, the mean-prior $\bar{\mu }$ and the variance-prior ${\overline{\sigma }}^{2}$ ($\bar{\mu }=0$, ${\overline{\sigma }}^{2}=1$ for simplicity in this paper), which is adjustable if there is prior knowledge about the two hyperparameters.

4 $$P({{{{{{\bf{w}}}}}}}_{{{{{{\mathrm{True}}}}}}}|{{{{{{\bf{X}}}}}}}_{{{{{{\mathrm{Train}}}}}}},{{{{{{\bf{Y}}}}}}}_{{{{{{\mathrm{Train}}}}}}})=\frac{P({{{{{{\bf{y}}}}}}}_{{{{{{\mathrm{Train}}}}}}}|{{{{{{\bf{X}}}}}}}_{{{{{{\mathrm{Train}}}}}}},{{{{{\bf{w}}}}}})}{P({{{{{{\bf{Y}}}}}}}_{{{{{{\mathrm{Train}}}}}}}|{{{{{{\bf{X}}}}}}}_{{{{{{\mathrm{Train}}}}}}})}\cdot P({{{{{\bf{w}}}}}})$$

5 $$P({{{{{\bf{w}}}}}})=\mathop{\prod }\limits_{i=1}^{c}\mathop{\prod }\limits_{j=1}^{n}p\left({\omega }_{j}^{(i)}\right)\sim N(0,I),\,p({\omega }_{j}^{i})\sim N\left(\overline{\mu },{\overline{\sigma }}^{2}\right)$$

6 $$	{D}_{KL}[Q({{{{{\bf{w}}}}}}|{{{{{{\bf{X}}}}}}}_{{{{{{\mathrm{Train}}}}}}},{{{{{{\bf{y}}}}}}}_{{{{{{\mathrm{Train}}}}}}})||P({{{{{{\bf{w}}}}}}}_{{{{{{\mathrm{True}}}}}}}|{{{{{{\bf{X}}}}}}}_{{{{{{\mathrm{Train}}}}}}},{{{{{{\bf{Y}}}}}}}_{{{{{{\mathrm{Train}}}}}}})]\\ 	 =\,\log [P({{{{{{\bf{Y}}}}}}}_{{{{{{\mathrm{Train}}}}}}}|{{{{{{\bf{X}}}}}}}_{{{{{{\mathrm{Train}}}}}}})]+{D}_{KL}[Q({{{{{\bf{w}}}}}}|{{{{{{\bf{X}}}}}}}_{{{{{{\mathrm{Train}}}}}}},{{{{{{\bf{y}}}}}}}_{{{{{{\mathrm{Train}}}}}}})||P({{{{{\bf{w}}}}}})]\\ 	 \,\,\,\,\,\,\,+{E}_{{{{{{\bf{w}}}}}}\sim Q\left({{{{{\bf{w}}}}}}|{{{{{{\bf{X}}}}}}}_{{{{{{\mathrm{Train}}}}}}},{{{{{{\bf{y}}}}}}}_{{{{{{\mathrm{Train}}}}}}}\right)}(\log [{(P({{{{{{\bf{y}}}}}}}_{{{{{{\mathrm{Train}}}}}}}|{{{{{{\bf{X}}}}}}}_{{{{{{\mathrm{Train}}}}}}},{{{{{\bf{w}}}}}}))}^{-1}])$$

Kullback–Leibler (KL) divergence is introduced to assess the similarity between the two posterior distributions mentioned above, as shown in Eq. (6). Moreover, the KL divergence result is the prototype of the loss function after substitution of the Bayesian formula (Eq. (4)) for the true posterior distribution in Eq. (6), as shown on the right-hand side of Eq. (6). The KL divergence on the left-hand side is determined by ${D}_{KL}[Q({{{{{\bf{w}}}}}}|{{{{{{\bf{X}}}}}}}_{{{{{{\mathrm{Train}}}}}}},{{{{{{\bf{y}}}}}}}_{{{{{{\mathrm{Train}}}}}}})||P({{{{{\bf{w}}}}}})]$, the KL divergence of the constructed posterior distribution $Q({{{{{\bf{w}}}}}}|{{{{{{\bf{X}}}}}}}_{{{{{{\mathrm{Train}}}}}}},{{{{{{\bf{y}}}}}}}_{{{{{{\mathrm{Train}}}}}}})$ from the joint prior distribution $P({{{{{\bf{w}}}}}})$ in the second part of the right-hand side; and the expectation of $\log {[P({{{{{{\bf{y}}}}}}}_{{{{{{\mathrm{Train}}}}}}}|{{{{{{\bf{X}}}}}}}_{{{{{{\mathrm{Train}}}}}}},{{{{{\bf{w}}}}}})]}^{-1}$ with respect to ${{{{{\bf{w}}}}}}\sim Q({{{{{\bf{w}}}}}}|{{{{{{\bf{X}}}}}}}_{{{{{{\mathrm{Train}}}}}}},{{{{{{\bf{y}}}}}}}_{{{{{{\mathrm{Train}}}}}}})$ in the last part of the right-hand side. When the data and true labels are provided, the first part $\log [P({{{{{{\bf{Y}}}}}}}_{Train}|{{{{{{\bf{X}}}}}}}_{Train})]$ on the right-hand side of Eq. (6) is a constant.

To minimize the KL divergence signified in Eq. (6), we must identify an appropriate constructed posterior distribution. Briefly, the loss function (Eq. (7)) consists of two parts: the second part of Eq. (6), shown in the summation of KL divergence of the constructed posterior from the prior for individual weight $\omega$ due to the independence among variables, inspiring the constructed posterior distribution close to the prior; and the last part of Eq. (6) encouraging predicted labels ${{{{{{\bf{y}}}}}}}_{{{{{{\mathrm{Train}}}}}}}$ close to true labels ${{{{{{\bf{Y}}}}}}}_{{{{{{\mathrm{Train}}}}}}}$ as far as possible w.r.t ${{{{{{\bf{y}}}}}}}_{{{{{{\mathrm{Train}}}}}}}\sim P({{{{{{\bf{y}}}}}}}_{{{{{{\mathrm{Train}}}}}}}|{{{{{{\bf{X}}}}}}}_{{{{{{\mathrm{Train}}}}}}},{{{{{\bf{w}}}}}})$ (the first part of Eq. (6) is omitted in the loss function), which can be transformed into a distance (the Euclidean distance in this paper) to estimate the similarity between the predicted labels and true labels. To minimize the loss, however, the two parts of Eq. (7) must be minimized concurrently while presenting with adversarial behaviors in their variations, which is the first adversarial strategy shown in the loss function of Fig. 10a.

7 $${F}_{loss}({{{{{\bf{w}}}}}})	 \simeq {D}_{KL}[Q({{{{{\bf{w}}}}}}|{{{{{{\bf{X}}}}}}}_{{{{{{\mathrm{Train}}}}}}},{{{{{{\bf{y}}}}}}}_{{{{{{\mathrm{Train}}}}}}})||P({{{{{\bf{w}}}}}})]+d({{{{{\bf{Y}}}}}},{{{{{\bf{y}}}}}})\\ 	 = \frac{1}{2}\mathop{\sum }\limits_{i=1}^{c}\mathop{\sum }\limits_{j=1}^{n}\left[\log \frac{{\overline{\sigma }}_{j}^{(i)2}}{{\sigma }_{j}^{(i)2}}+\frac{\left[{\sigma }_{j}^{(i)2}+{\left({\mu }_{j}^{(i)}-{\overline{\mu }}_{j}^{(i)}\right)}^{2}\right]}{{\overline{\sigma }}_{j}^{(i)2}}-1\right]+d({{{{{\bf{Y}}}}}},{{{{{\bf{y}}}}}})$$

8 $${{{{{{\bf{y}}}}}}}_{{{{{{\mathrm{Train}}}}}}}={{{{{{\bf{y}}}}}}}_{\mu }+\theta \odot \sqrt{{{{{{{\bf{y}}}}}}}_{{\sigma }^{2}}}=({{{{{{\bf{X}}}}}}}_{{{{{{\mathrm{Train}}}}}}}\cdot {{{{{\boldsymbol{\mu }}}}}}+{b}_{\mu })+\theta \odot \sqrt{({{{{{{\bf{X}}}}}}}_{{{{{{\mathrm{Train}}}}}}}\cdot {{{{{{\boldsymbol{\sigma }}}}}}}^{2}+{b}_{{\sigma }^{2}})}$$

In algorithm practice, the mean layer and variance layer shown in Fig. 9, are both constructed with a fully connected layer each to confirm the values of $\mu$ and ${\sigma }^{2}$ for every $\omega$, so that the predicted label ${y}_{Train}$ in Eq. (8) is the sum of the mean results ${{{{{{\bf{y}}}}}}}_{\mu }$ and square root of the variance results ${{{{{{\bf{y}}}}}}}_{{\sigma }^{2}}$. Explicitly, the mean layer assesses the relevance between weights and specific experimental conditions given the multivariate effect when the robustness of the mean layer is assessed by the corresponding noise interferences implemented by the variance layer. Given the differentiability of two layers when stochastic gradient descent (SGD) is utilized in computation, a reparameterization trick is applied with a Gaussian matrix of the same shape as ${{{{{{\bf{y}}}}}}}_{\mu }$ and ${{{{{{\bf{y}}}}}}}_{{\sigma }^{2}}$. Here, elements $\theta \sim N(0,1)$ and $\odot$ signifies the Hadamard product in Eq. (8). By introducing a reparameterization matrix, we can fulfil the sampling for weight ${{{{{\bf{w}}}}}}$ from the constructed Gaussian distribution $N(\mu ,{\sigma }^{2})$, and then sample the actual ${{{{{{\bf{y}}}}}}}_{Train}$ from its distribution to estimate the distance from true labels ${{{{{{\bf{Y}}}}}}}_{Train}$ denoted by the second part on the right-hand side of Eq. (7).

### Feature evaluation

Feature Evaluation, the last stage of an intact computation iteration, is designed to distinguish the productive features from the feature batch according to the model parameters confirmed by the Model Training and Test stage.

The mean layer weights matrix and variance layer weights matrix of the linear model describe the constructed posterior distributions for the independent weights, ${{{{{\boldsymbol{\omega }}}}}}[i,j] \sim {{{{{\bf{N}}}}}}({{{{{\boldsymbol{\mu }}}}}}[i,j],{{{{{{\boldsymbol{\sigma }}}}}}}^{2}[i,j])$, where ${{{{{\boldsymbol{\mu }}}}}}[i,j]$ is the element in position $[i,j]$ of the mean layer weights matrix with dimension n*C. As noted in the Feature Evaluation part of Fig. 9, every column of the weights matrix is related to one single stimulus condition. We set the mean_norm $\widehat{\mu }$ and variance_norm ${\widehat{\sigma }}^{2}$ as the informative Feature Evaluation norm.

First, all features are required to be evaluated one by one. Explicitly, the actual position of the Selection Matrix ${{{{{{\boldsymbol{\rho }}}}}}}_{i}[i,j]$ is set to zero if the corresponding ${{{{{\boldsymbol{\mu }}}}}}[i,j]\le \widehat{\mu }$ and ${{{{{{\boldsymbol{\sigma }}}}}}}^{2}[i,j]\le {\widehat{\sigma }}^{2}$. Second, the Selection Matrix is averaged across columns to obtain the Selection Array ${\lambda }_{i}$. Third, the elements in the Sparsity Selection Array ${\gamma }_{i}$ are set to zero (the magenta elements shown in Fig. 9) when the corresponding elements of Selection Array ${\lambda }_{i}$ are equal to zero and one otherwise (the green elements shown in Fig. 9). In general, the features whose weights related to all conditions are intensively distributed around meaningless points are eliminated in the following computation iterations. Here, ${\gamma }_{i}$ is the Sparsity Position Array characterizing the evaluation results for the feature batch analyzed in the Time_i Model Training and Test stage, which needs to be transformed into the Backfilled Array ${\tau }_{i}$ with dimensions $[K*1]$ ($K=n+g$, where $g$ is the number of unproductive feature positions assessed in the previous iterations), signifying the feature positions among the completed data dimensionality according to the Position Array used in the Time_i Feature Selection stage. Finally, the Time_(i + 1) Sub-Position Array ${\zeta }_{i+1}$ with dimensions $[L*1]$ ($L=n+g+l$, where $l$ equals the number of unproductive feature positions assessed in the Time_i Feature Evaluation stage) is constructed from ${\tau }_{i}$ by setting the new position to 1 for the features compensated to meet max_fea. Collectively, the Time_(i + 1) Sub-Position Array ${\zeta }_{i+1}$ is composed of these parts: the positions of productive elements assessed in the Time_i Feature Evaluation stage (the green elements depicted in Fig. 9), the positions of compensated elements (the yellow elements depicted in Fig. 9), and the zero parts, including the positions of elements estimated in the Time_i Feature Evaluation stage and the elements estimated in previous iterations (the magenta elements depicted in Fig. 9).

The second adversarial strategy denoted in Fig. 10b is implied in the Feature Evaluation stage. The original distribution $N(\tilde{\mu },{\tilde{\sigma }}^{2})$ is the default value of the constructed posteriors for ${{{{{\bf{w}}}}}}$ ($Q({{{{{\bf{w}}}}}}|{{{{{{\bf{X}}}}}}}_{{{{{{\mathrm{Train}}}}}}},{{{{{{\bf{y}}}}}}}_{{{{{{\mathrm{Train}}}}}}})$ in Eq. (3)) shown in the red line of Fig. 10(b). The prior distribution $N(\overline{\mu },{\overline{\sigma }}^{2})$ ($P({{{{{\bf{w}}}}}})$ in Eq. (5)) shown in the black line of Fig. 10b, is a key part of the first adversarial strategy described in the Model Training and Test stage. The norm distribution $N(\widehat{\mu },{\widehat{\sigma }}^{2})$ is the quantified feature evaluation norm and is signified in the blue line of Fig. 10b. The values of the three distributions meet the relationship described below.

$$\begin{array}{cc}\overline{\mu } \, < \,\tilde{\mu }\, < \,\widehat{\mu } & {\widehat{\sigma }}^{2}\, < \,{\tilde{\sigma }}^{2}\, < \,{\overline{\sigma }}^{2}\end{array}$$

The first adversarial strategy equally encourages the predicted labels ${{{{{{\bf{y}}}}}}}_{{{{{{\mathrm{Train}}}}}}}$ to be similar to the true labels ${{{{{{\bf{Y}}}}}}}_{{{{{{\mathrm{Train}}}}}}}$ and constructed posterior for ${{{{{\bf{w}}}}}}$ to be closed to the prior distribution in the Model Training and Test stage. In practice, the majority of $\omega$ exhibit distributions that are closed to the prior distribution, whereas only a minority of $\omega$ exhibit distributions that are concentrated on some significant points after the Model Training and Test stage. The second adversarial strategy is designed to ultimately select the informative features concentrated at significant points, with the assistance of a contradictory relationship between the norm distribution direction and the prior distribution direction.

A complete computation iteration consists of the Feature Selection, Model Training and Test, and Feature Evaluation stages as depicted in blue in Fig. 9. At the last part of a computation iteration, the Sub-Position Array needed in the next iteration is settled. The number of iterations to achieve whole model convergence is determined with the feature compensation efficiency for the new iteration. In particular, the number of features $n$ computed simultaneously is either equal to max_fea when the number of features not computed previously is sufficient for compensation or the number of informative features assessed across the finished iterations.

### Feature identification for every condition

After the overall features are computed once, the informative features continue to be assessed by the computation iterations until the Position Array of a new iteration is the same as that of the last iteration (or the Position Array and Sparsity Selection Array of one computation iteration are same as each other) to select the least number of productive features needed to guarantee the model convergence, as denoted in Fig. 9. Furthermore, every column in the Sparsity Selection Matrix of the last computation iteration denotes the disparate selected features related to specific experimental conditions (one of the $C$ classes), which can be reshaped into the same shape as that of the input volume and overlaid on a standard template to study the spatial distribution characteristics.

The classification task is performed by two parallel, fully connected layers. The key efficacy in the VRE design is the strategy for discriminating the informative voxels from a very large number of irrelevant alternatives. Actually, the VRE allows an exact evaluation of the features one by one using the batch-feature-input trick, by which the feature number analyzed simultaneously is reduced to a proper range to substantially prevent overfitting on a comparatively minor dataset.

#### Relevance index

Here, we propose a new index, the Relevance index (RI), to evaluate the classification contribution of every feature for discriminating the correct category from others (classification specificity for the correct category compared to other alternatives).

9 $$R{I}_{c-1}^{{X}^{i}}=	 \frac{{x}^{i}\left({w}_{{\mu} {\_}{1}}^{i}-{w}_{{\mu} {\_}{c}}^{i}\right)}{\left({y}_{{\mu} {\_}{1}}-{b}_{{m}\_{1}}\right)-\left({y}_{{\mu} {\_}{c}}-{b}_{{m}\_{c}}\right)}\cdot \frac{{y}_{{\mu} {\_}{1}}-{y}_{{\mu }{\_}{c}}}{{y}_{1}^{*}-{y}_{c}}\\ 	 +\frac{{x}^{i}\left({w}_{{\sigma }^{2}{\_}1}^{i}-{w}_{{\sigma }^{2}{\_}c}^{i}\right)}{\left({y}_{{\sigma }^{2}{\_}1}^{2}-{b}_{v{\_}1}\right)-\left({y}_{{\sigma }^{2}{\_}c}^{2}-{b}_{v{\_}c}\right)}\cdot \frac{{y}_{{\sigma }^{2}{\_}1}\cdot {\theta }_{1}^{{x}^{i}}-{y}_{{\sigma }^{2}{\_}c}\cdot {\theta }_{c}^{{x}^{i}}}{{y}_{1}^{*}-{y}_{c}}$$

10 $$R{I}^{{x}^{i}}=(R{I}_{1}^{{x}^{i}}+\cdot \cdot \cdot +R{I}_{c-1}^{{x}^{i}}+\cdots R{I}_{C-1}^{{x}^{i}})/C-1$$

As Fig. 1(b) shows, we gather the predicted result of an N-dimensional volume belonging to Class_1 implemented by the two parallel layers after the computation finish. Therefore, the predicted result of this Class_1 volume is a C-dimensional vector, the first element of which is the maximum element among the overall elements. Then, the sub-relevance index is the proportion voxel_i ${x}^{i}$ comprises in the difference between the predicted result element of the correct category (the first element ${y}_{1}^{*}$ of the predicted label in Fig. 1b) and that of one of the other alternatives (another element ${y}_{c}$ of the predicted label in Fig. 1b), as shown in Eq. (9). On the right-hand side of Eq. (9), the first part signifies the ratio of the difference between ${y}_{1}^{*}$ and ${y}_{c}$ caused by feature ${x}^{i}$ in the mean layer, whereas the second part denotes the same ratio caused by feature ${x}^{i}$ in the variance layer. Here, ${y}_{\mu \_{{{{{\bf{1}}}}}}}$ is the predicted label element of category_1 from the mean layer, ${b}_{m\_1}$ is the bias element of category_1 from the mean layer, and ${w}_{\mu \_1}^{i}$ is the weights of feature ${x}^{i}$ related to category_1 in the mean layer. For the feature${x}^{i}$, the two parts on the right-hand side in Eq. (9) are the contributions from the mean layer and variance layer for correct classification, and the mean of the (C-1) sub-relevance indices is the RI of this voxel, as depicted in Eq. (10). By temporally arranging the RIs of one feature analyzed from volumes collected consecutively (related to one condition), the cognitive state dynamic of the voxel is obtained. Additionally, the RI of all voxels constitutes the cognitive state matrix of this volume, and the cognitive state dynamics regarding a certain cognitive function are composed of the state matrices analyzed from continuous volumes collected during task performance.

Collectively, we can further study the relevance index clustering positions (the sum of the RIs of the whole-brain selected voxels is equal to 1; the RI of one selected voxel cluster is the summation of the RIs of the voxels within it.) across the whole-brain to describe the category-specific distribution of a certain cognitive state by overlaying the cognitive state matrix on a standard template; and investigate the cognitive state dynamics of single voxels related to different condition in the temporal domain.

#### Leave-20%-volume-out cross-validation

The accuracy of classification performance is typically evaluated using cross-validation, which can be conducted iteratively so that every sample in the dataset can be chosen as test data or training data, yielding an overall measure of classification accuracy. For every volume, a cognitive state matrix consisting of the RIs of all voxels selected by the VRE is obtained. In the case of state imbalance due to different training/test dataset segmentations, every volume has an opportunity to be a part of the training or test dataset based on the hypothesis that every volume in a block might signify a different cognitive state. For example, eight volumes are collected during a stimulus block, yielding 28 different datasets (${C}_{8}^{2}=28$), which is also the number of VRE models that need to be constructed for a classification task if the training/test segmentation proportion equals 80%/20%. Finally, the cognitive state dynamic for a single voxel is the mean of different cognitive state dynamics analyzed by disparate models on their corresponding datasets.

### Reporting summary

Further information on research design is available in the Nature Portfolio Reporting Summary linked to this article.

## Supplementary information

Supplementary Information

Reporting Summary

## Data Availability

The datasets generated during the current study are available in the figshare repository (https://figshare.com/articles/dataset/VRE_01_Data/22203127). All other data were available from the corresponding author on reasonable request.

## Code availability

The custom codes that support the findings of this study are available from the corresponding author or first author upon reasonable request.
