# Supplementary material for: Variational relevance evaluation of individual fMRI data enables deconstruction of task-dependent neural dynamics
Source: Commun Biol. 2023 May 5;6:491. doi: 10.1038/s42003-023-04804-3 (PMC10163018; doi:10.1038/s42003-023-04804-3)
Supplement: Supplementary file 1 — Supplementary Information [file 42003_2023_4804_MOESM1_ESM.pdf]

Supplementary Information

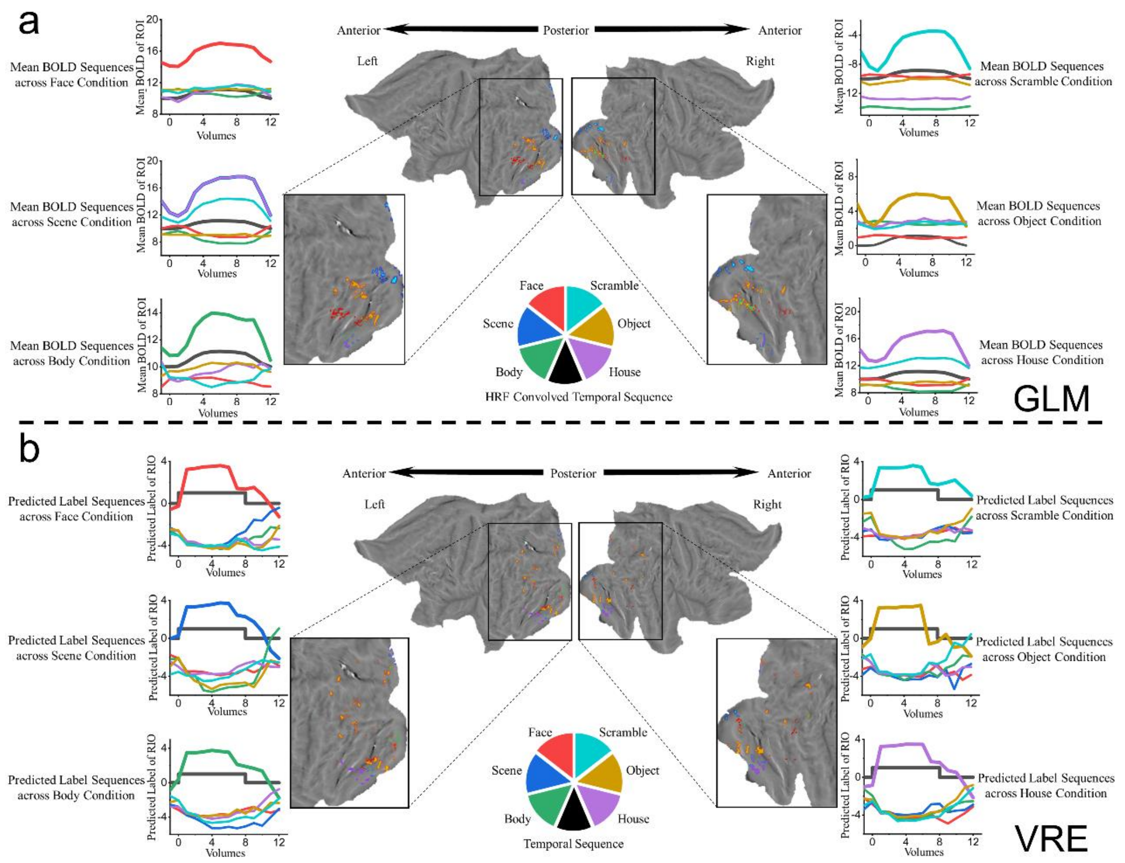

Supplementary Fig. 1 Individual object-selective results selected by the GLM and VRE for Subject_02.

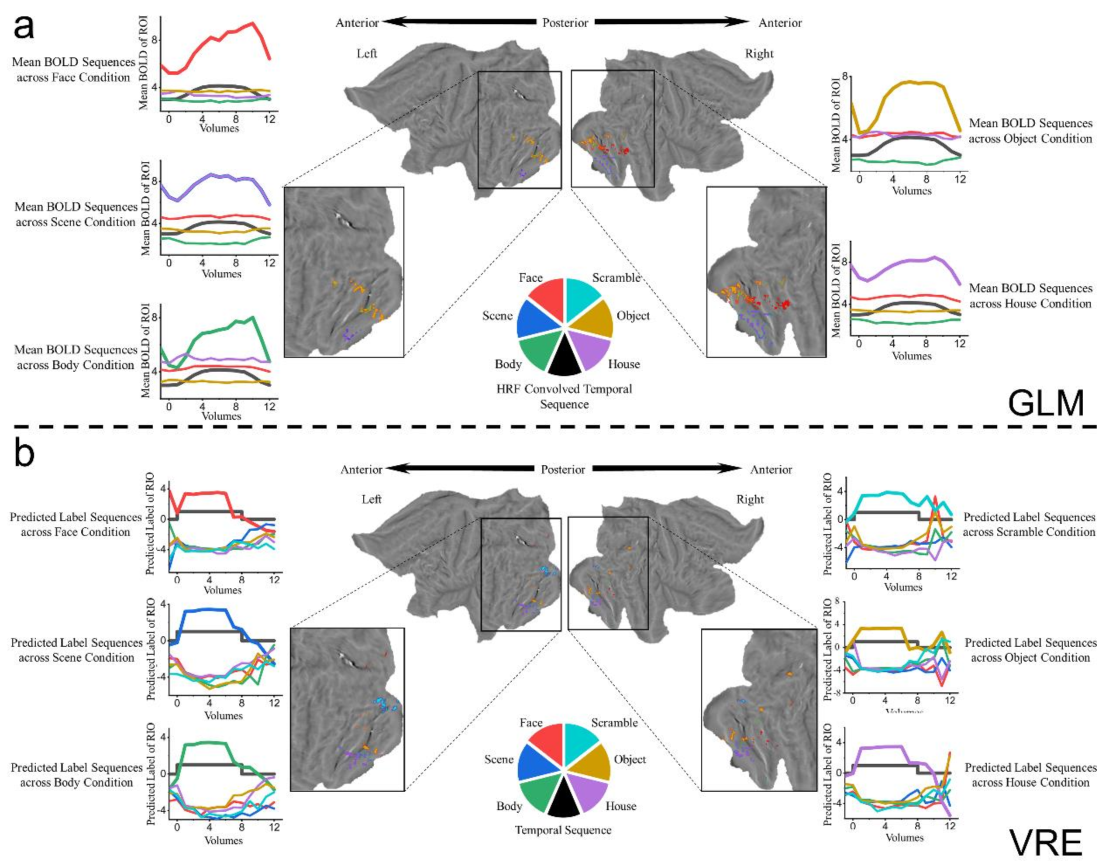

Supplementary Fig. 2 Individual object-selective results selected by the GLM and VRE for Subject_03.

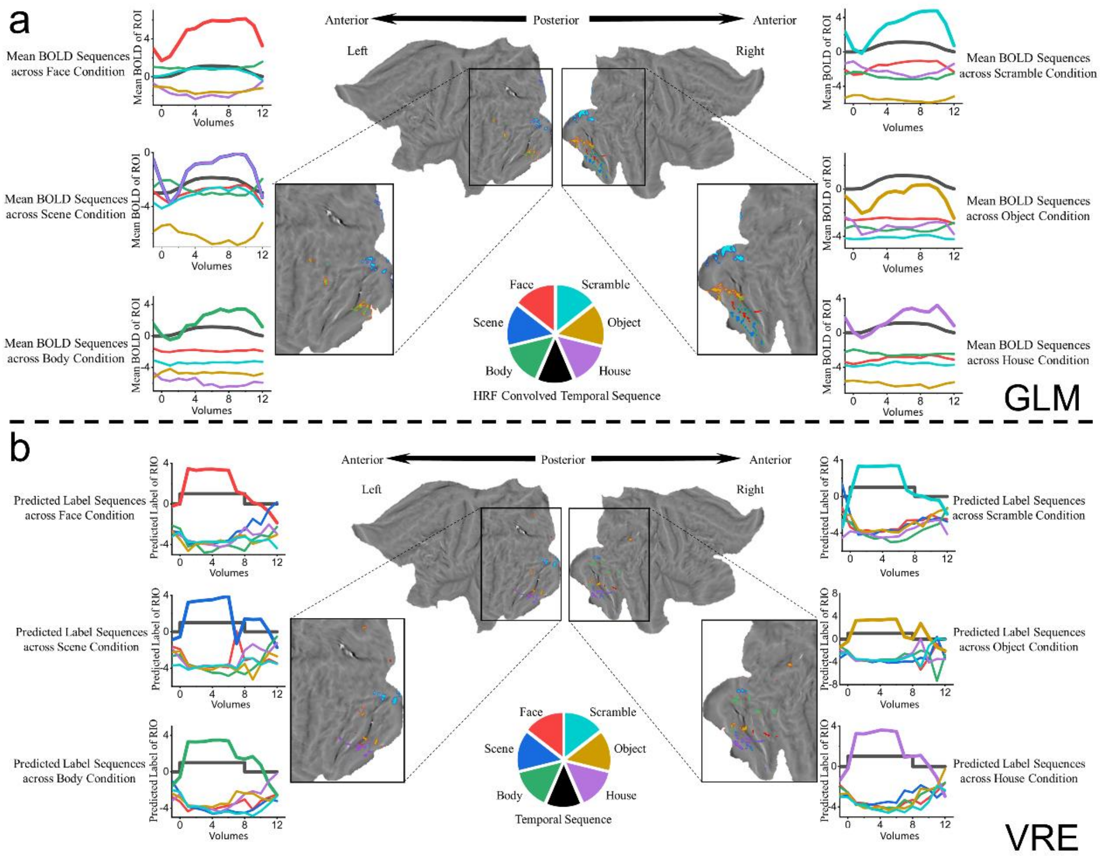

Supplementary Fig. 3 Individual object-selective results selected by the GLM and VRE for Subject_04.

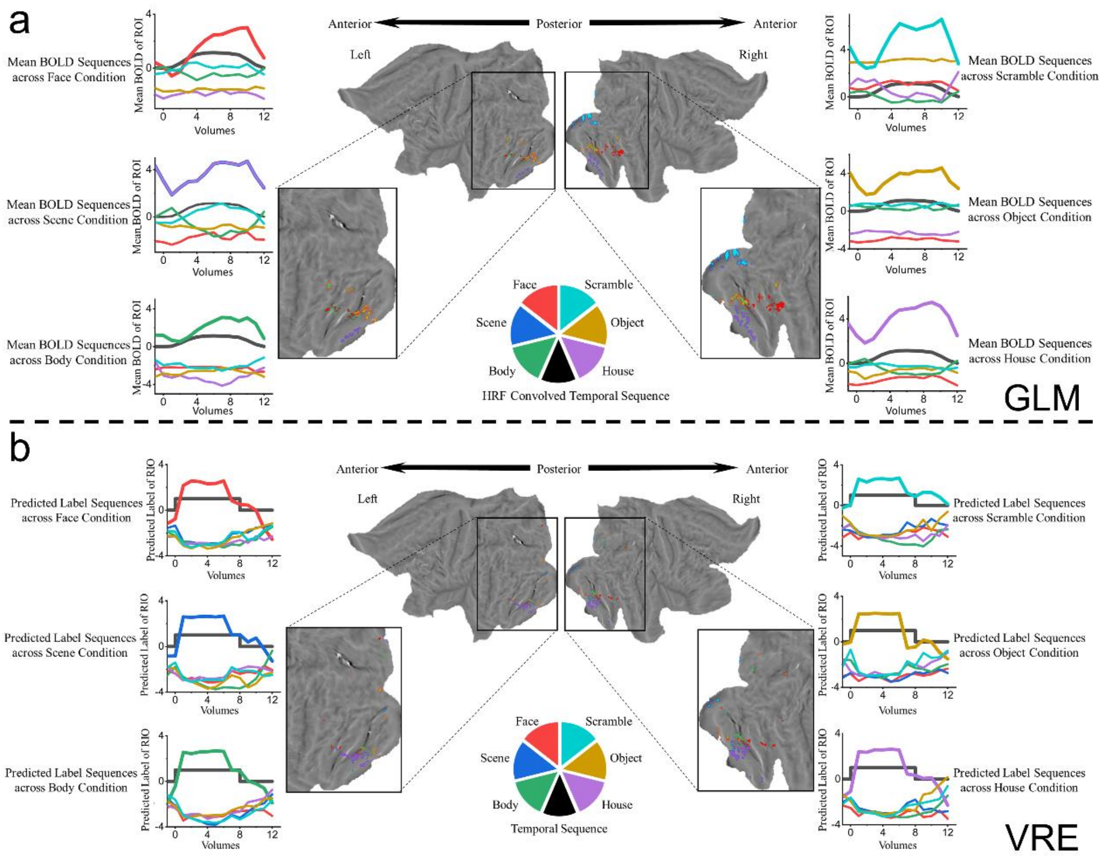

Supplementary Fig. 4 Individual object-selective results selected by the GLM and VRE for Subject_05.

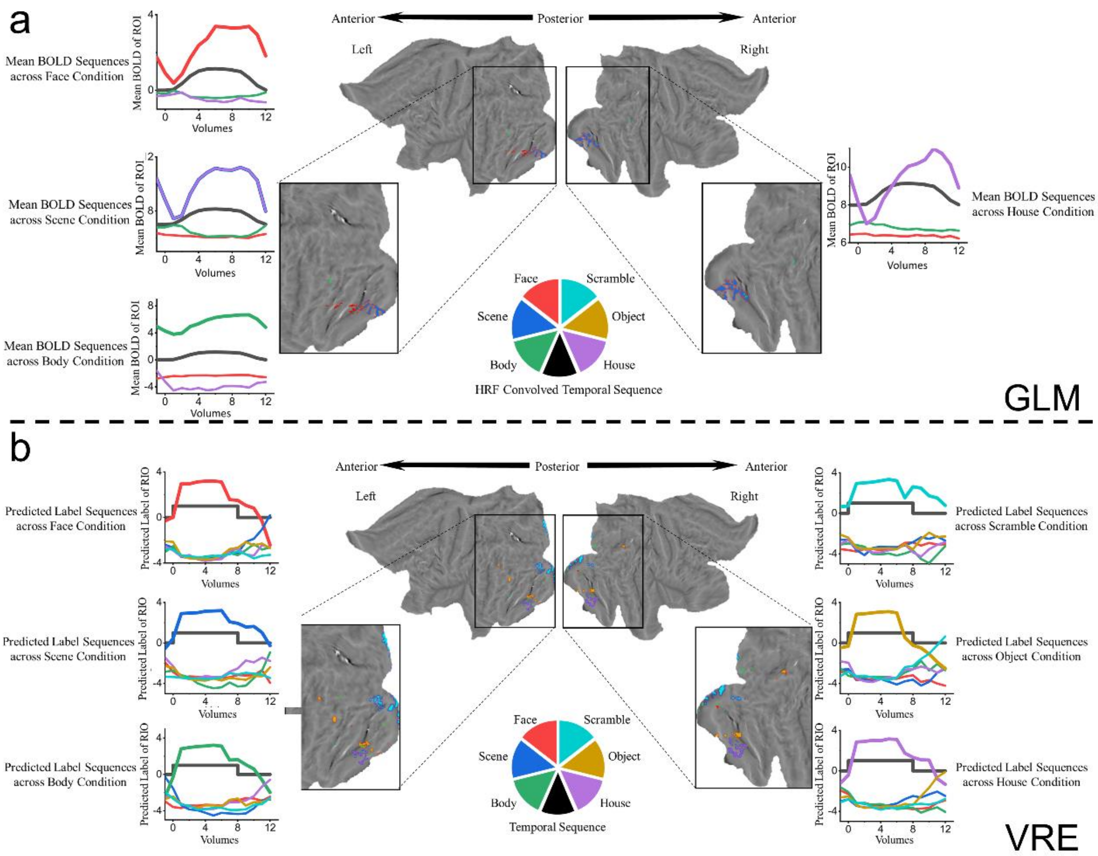

Supplementary Fig. 5 Individual object-selective results selected by the GLM and VRE for Subject_06.

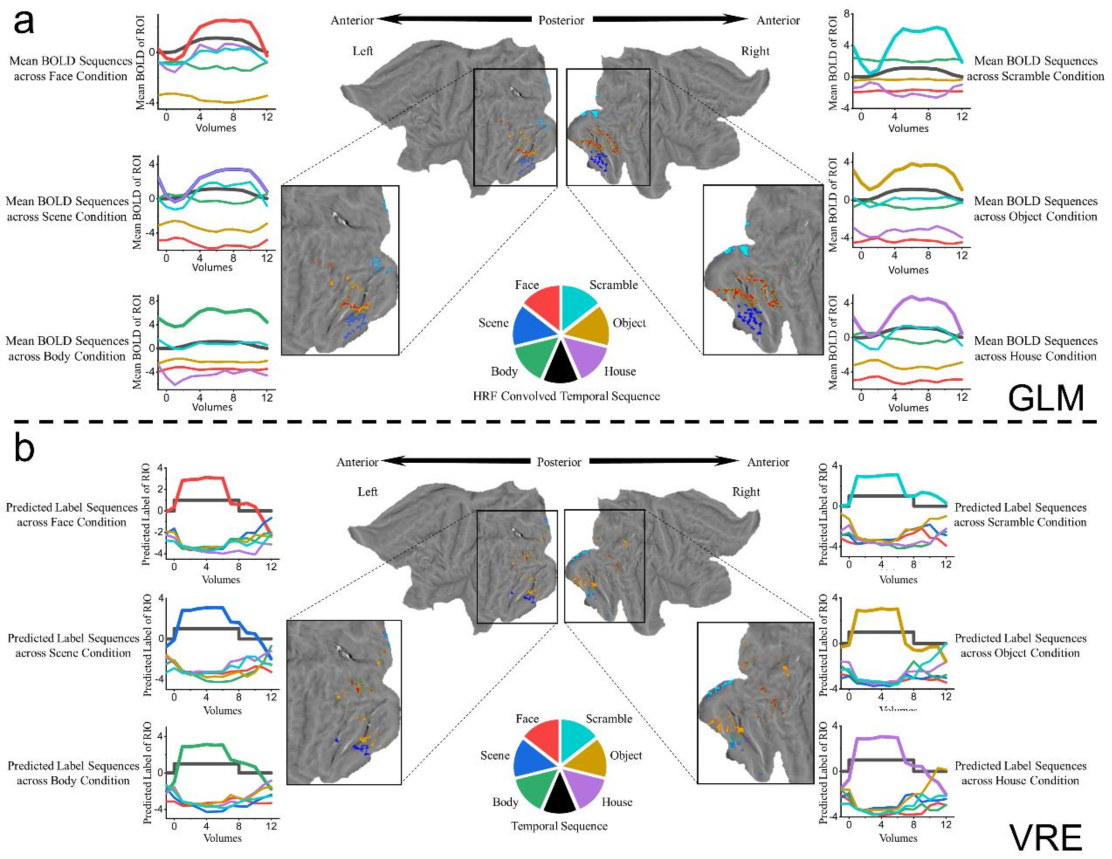

Supplementary Fig. 6 Individual object-selective results selected by the GLM and VRE for Subject_07.

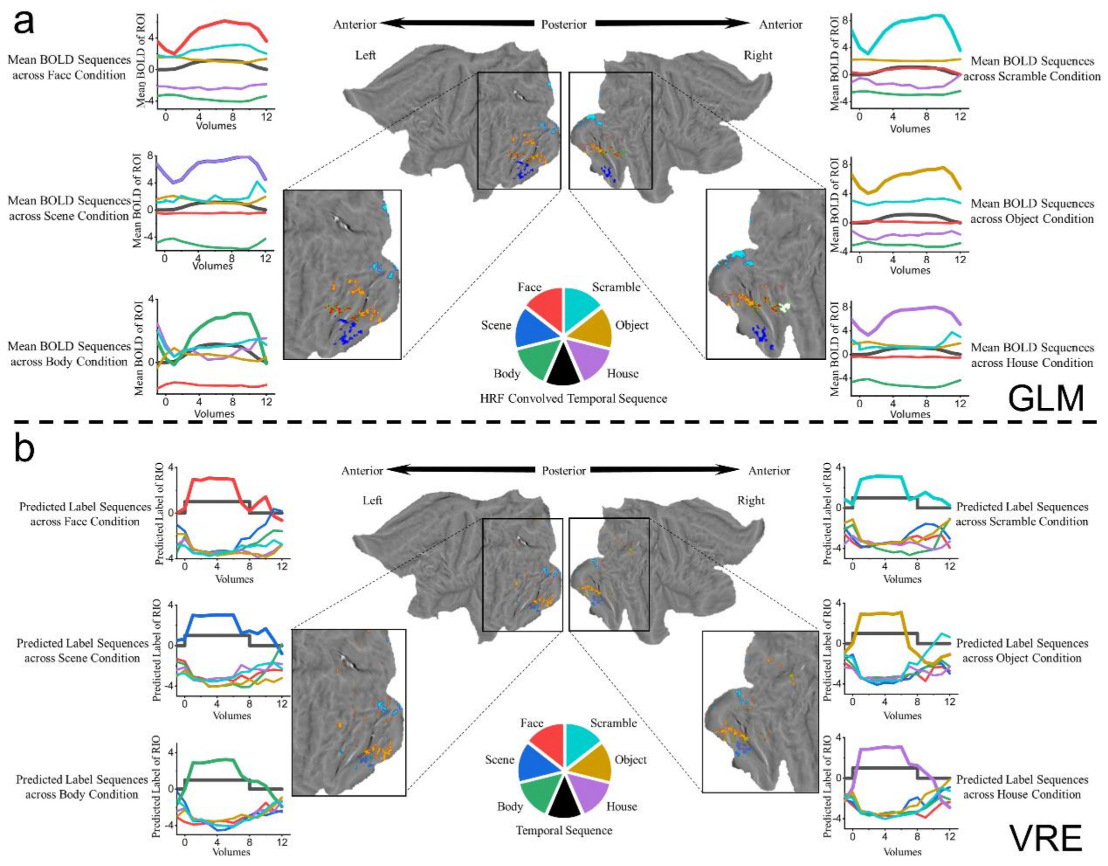

Supplementary Fig. 7 Individual object-selective results selected by the GLM and VRE for Subject_08.

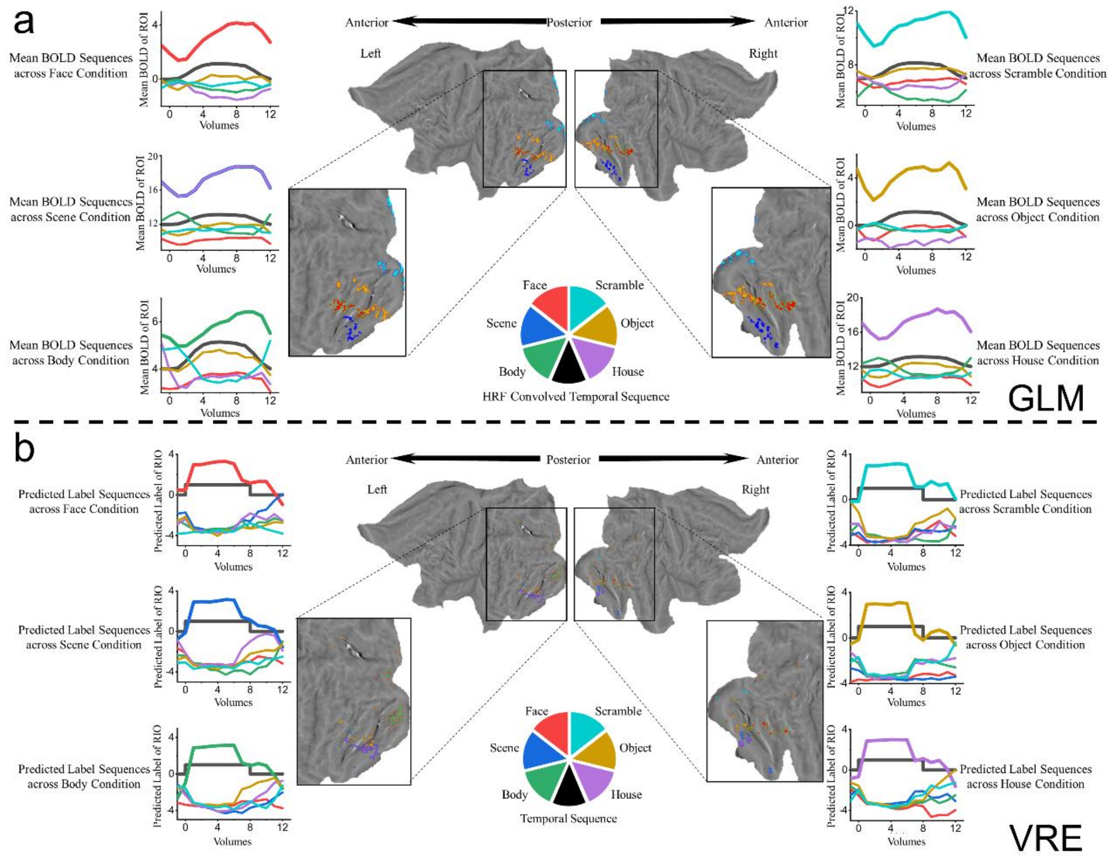

Supplementary Fig. 8 Individual object-selective results selected by the GLM and VRE for Subject_09.

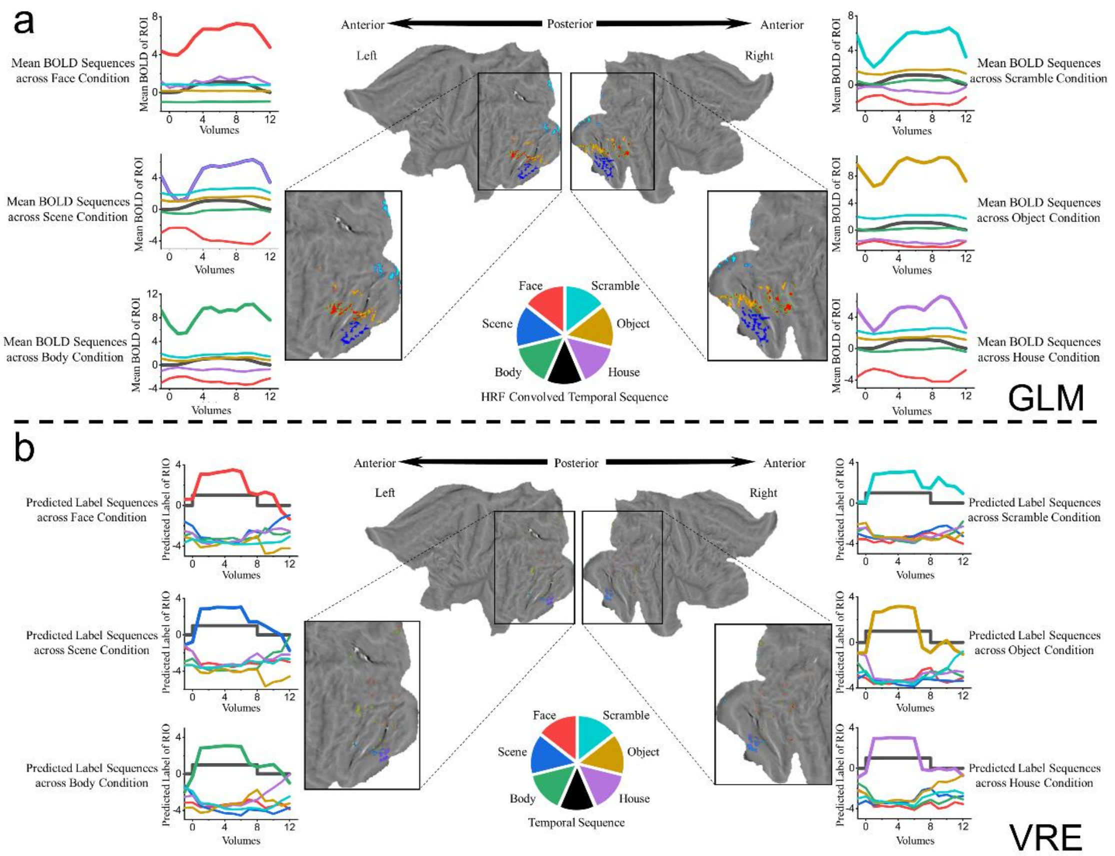

Supplementary Fig. 9 Individual object-selective results selected by the GLM and VRE for Subject_10.

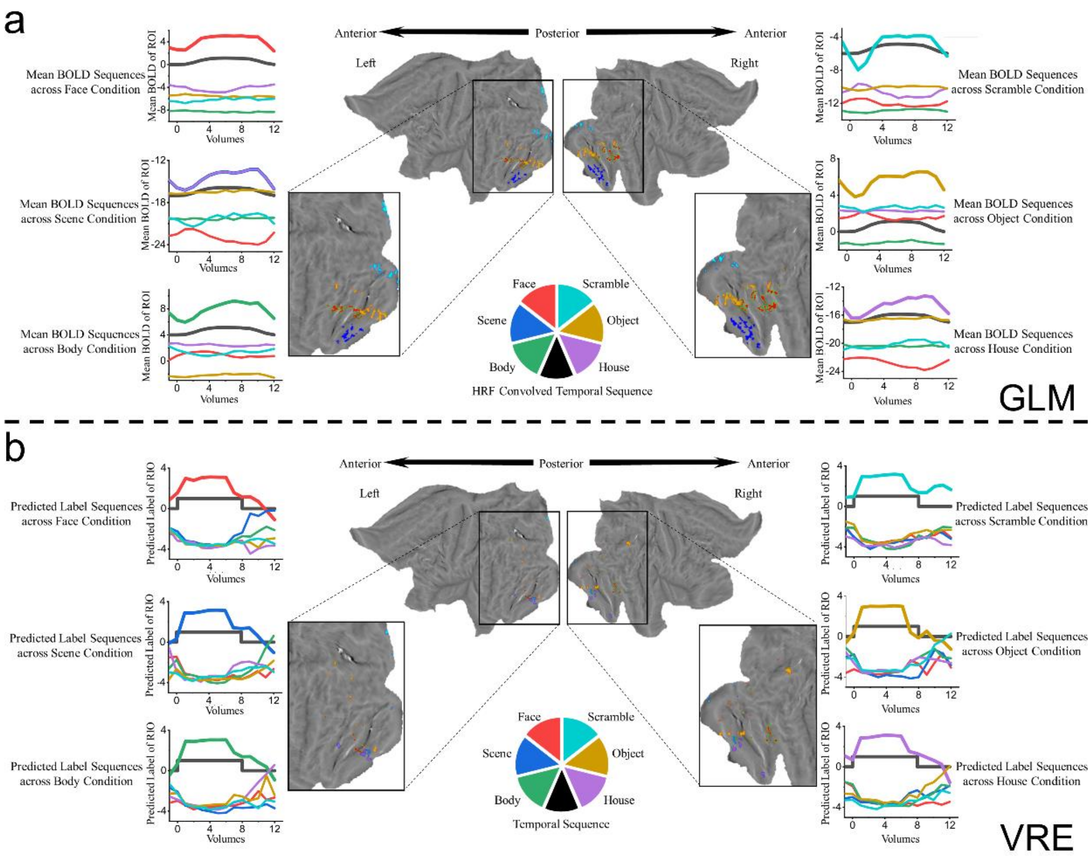

Supplementary Fig. 10 Individual object-selective results selected by the GLM and VRE for Subject_11.

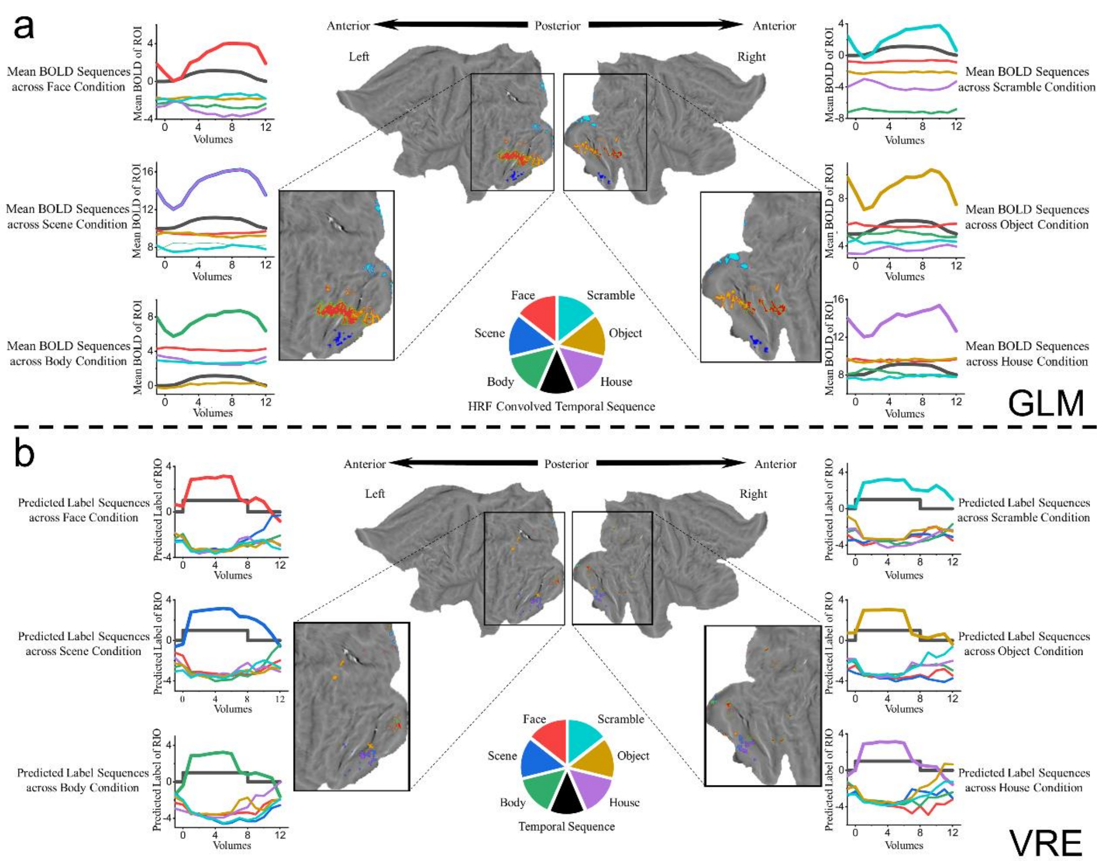

Supplementary Fig. 11 Individual object-selective results selected by the GLM and VRE for Subject_12.

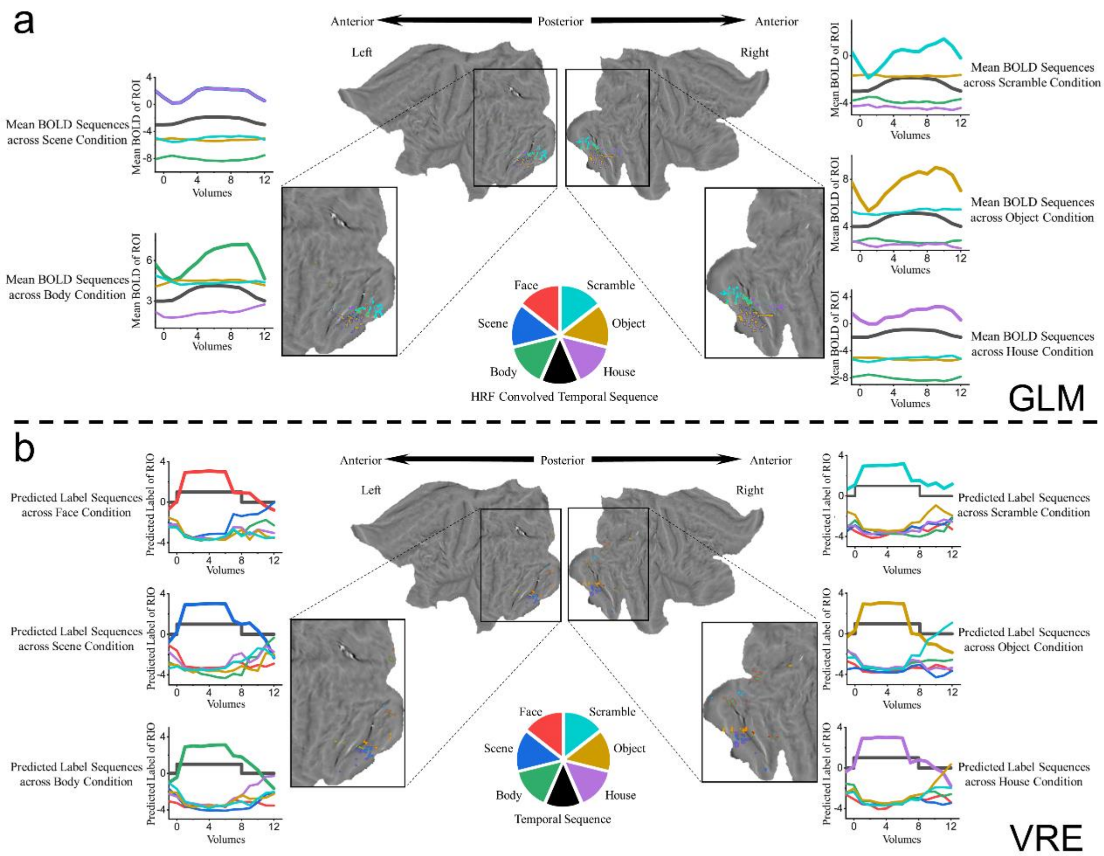

Supplementary Fig. 12 Individual object-selective results selected by the GLM and VRE for Subject_13.

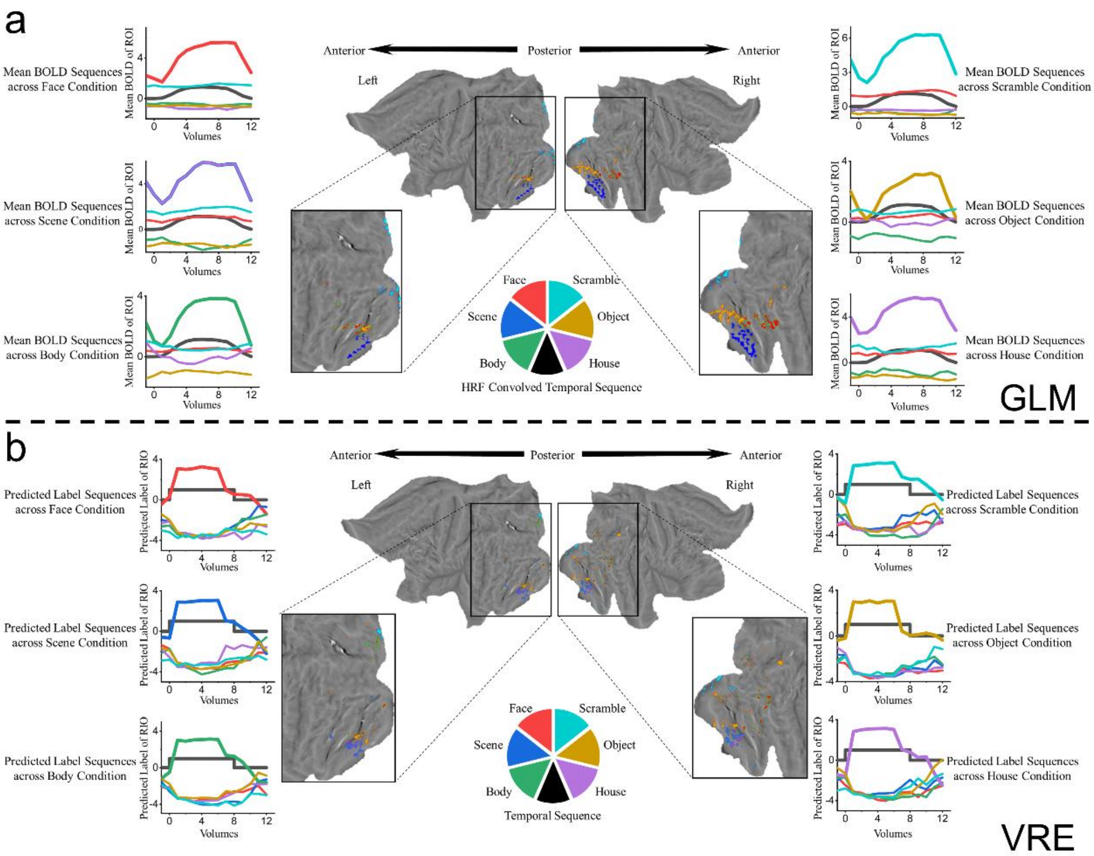

Supplementary Fig. 13 Individual object-selective results selected by the GLM and VRE for Subject_14.

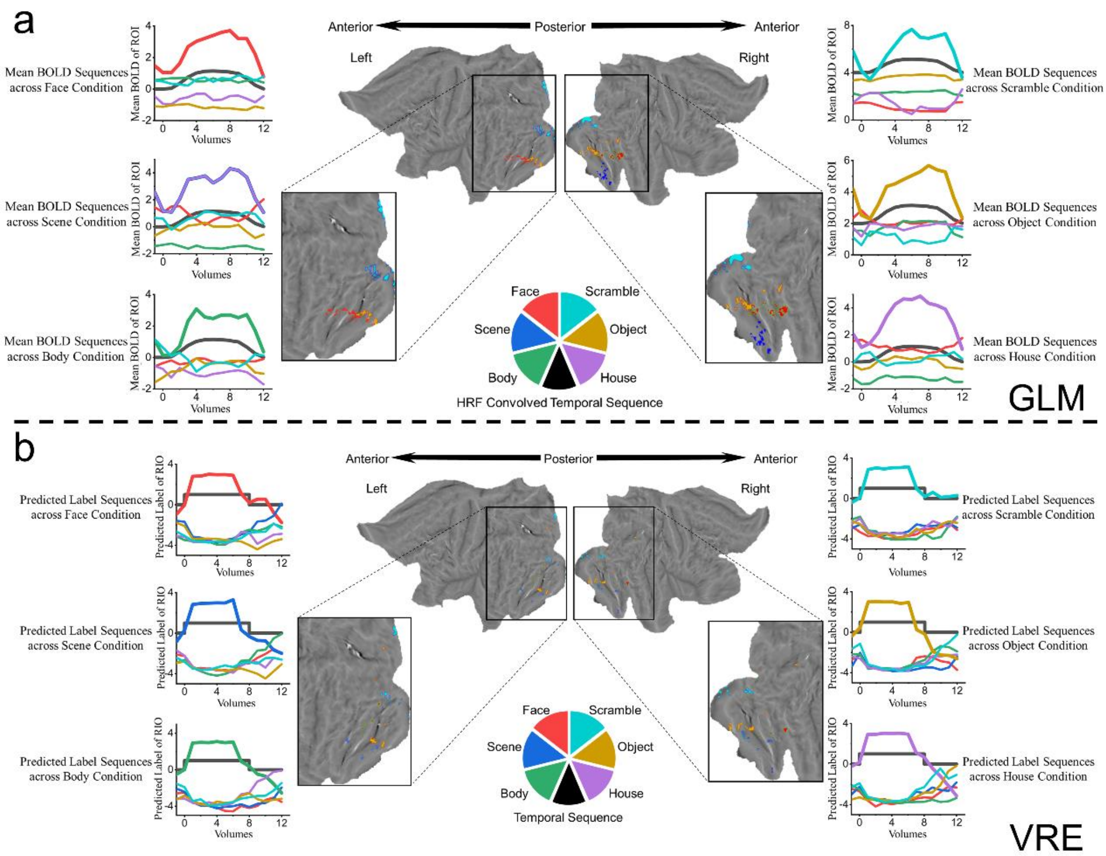

Supplementary Fig. 14 Individual object-selective results selected by the GLM and VRE for Subject_15.

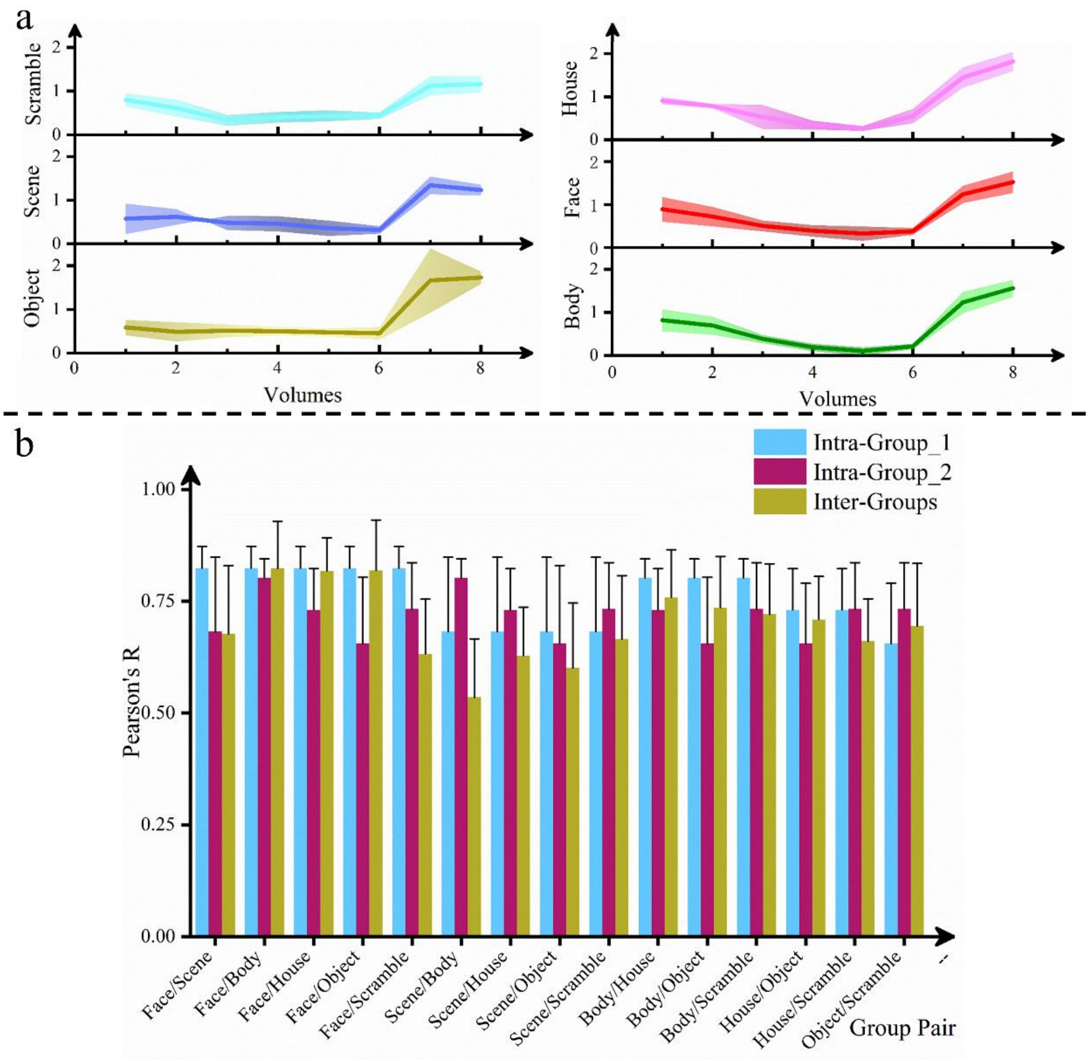

Supplementary Fig. 15 Dominating dynamics of six stimulus-responsive regions and their Pearson's correlation analysis results for Subject _02.

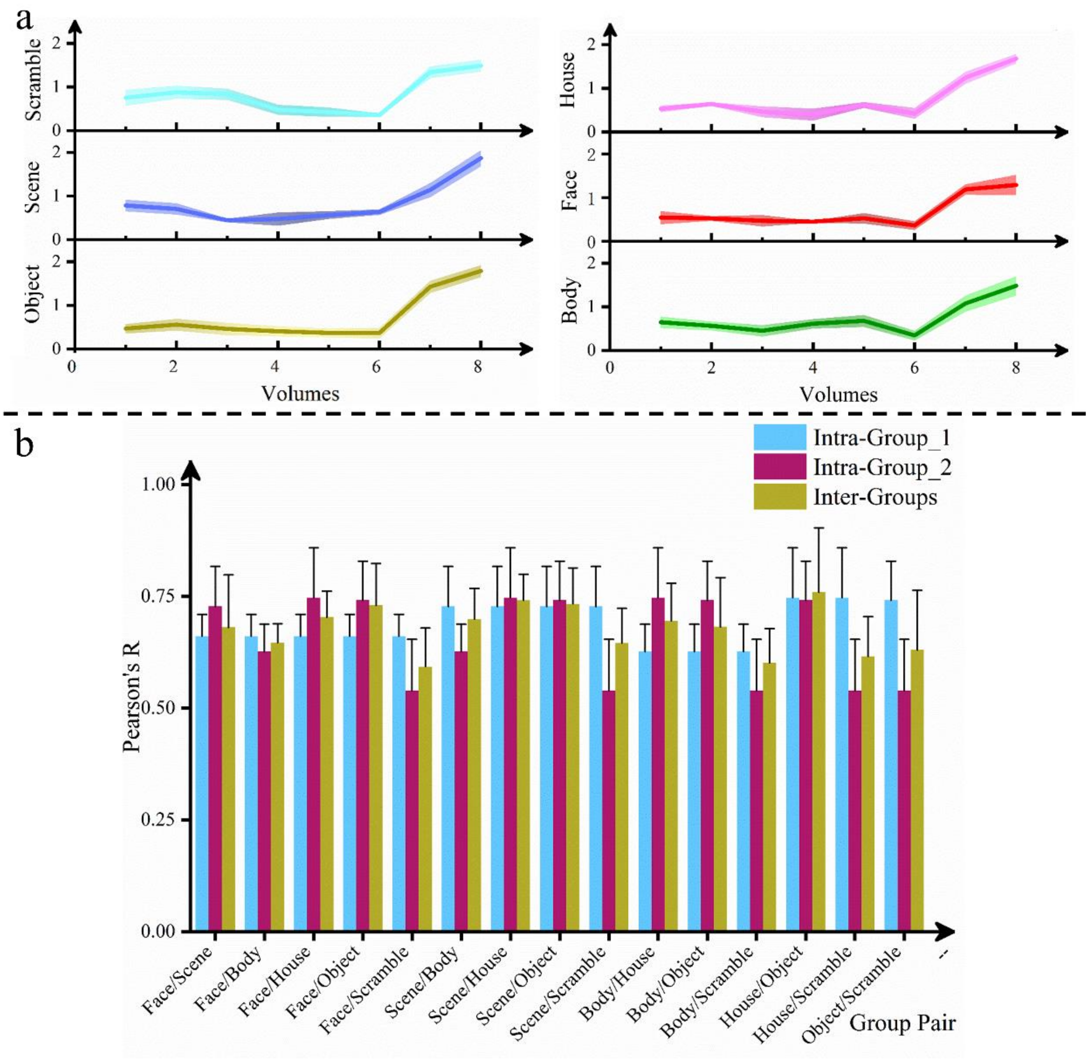

Supplementary Fig. 16 Dominating dynamics of six stimulus-responsive regions and their Pearson's correlation analysis results for Subject _03.

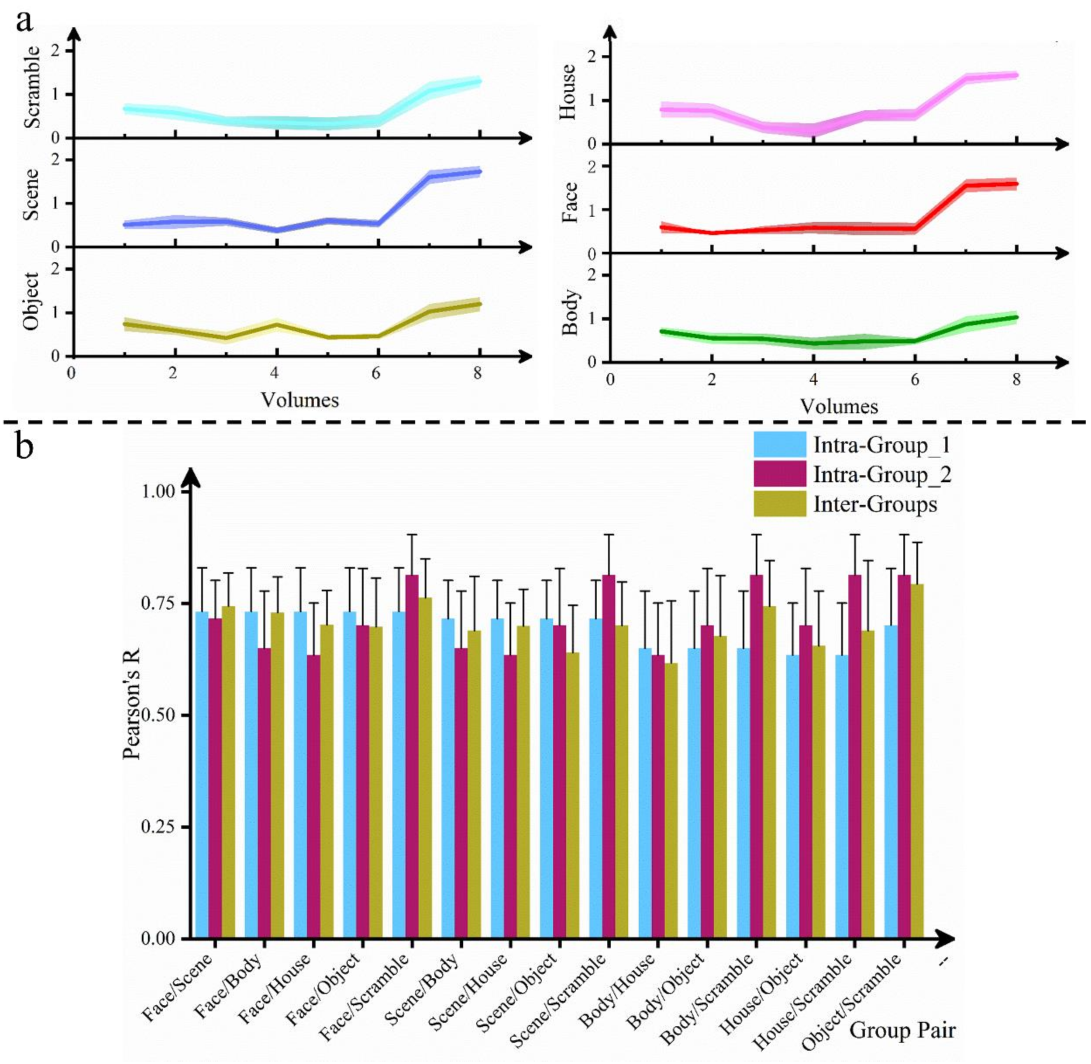

Supplementary Fig. 17 Dominating dynamics of six stimulus-responsive regions and their Pearson's correlation analysis results for Subject _04.

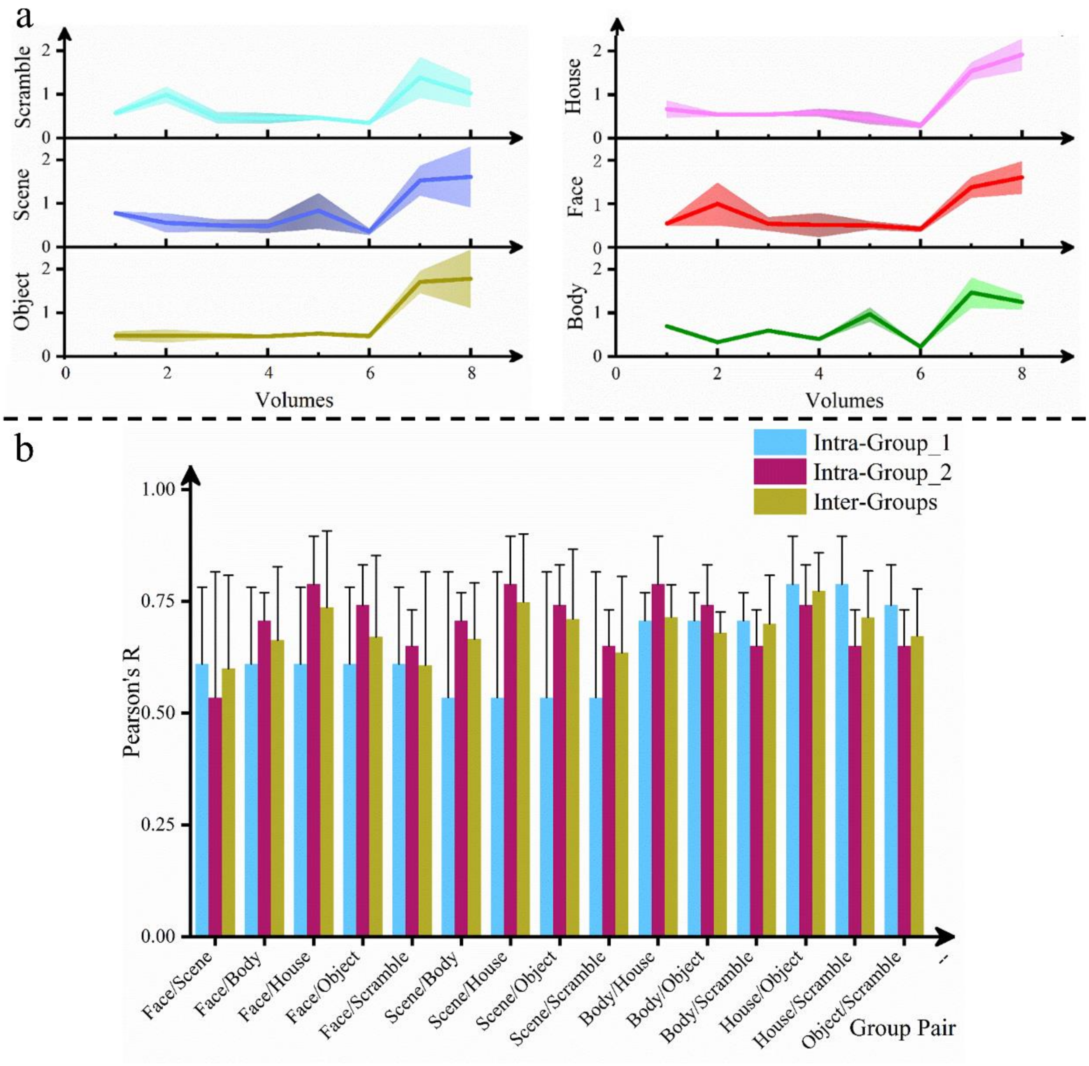

Supplementary Fig. 18 Dominating dynamics of six stimulus-responsive regions and their Pearson's correlation analysis results for Subject _05.

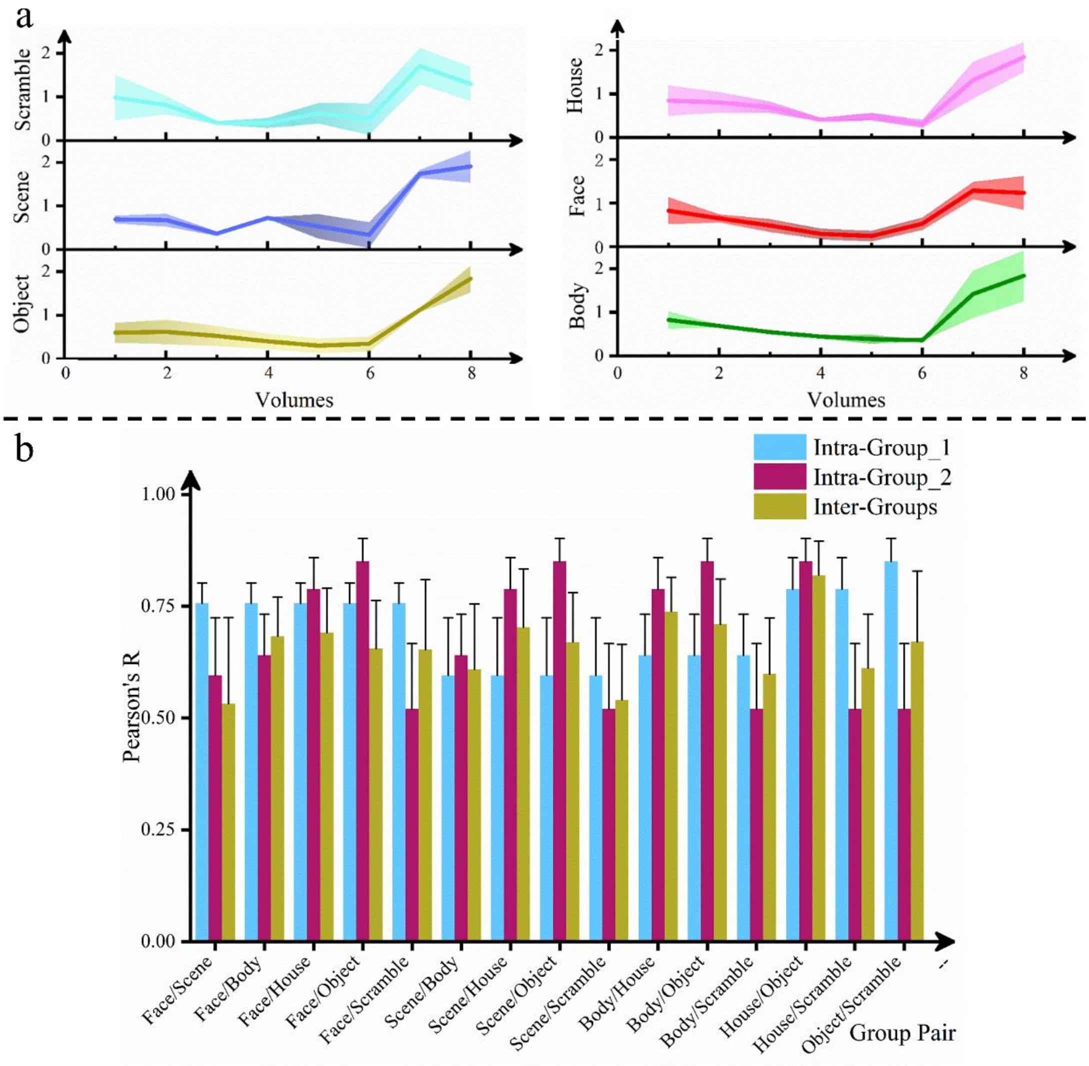

Supplementary Fig. 19 Dominating dynamics of six stimulus-responsive regions and their Pearson’s correlation analysis results for Subject _06.

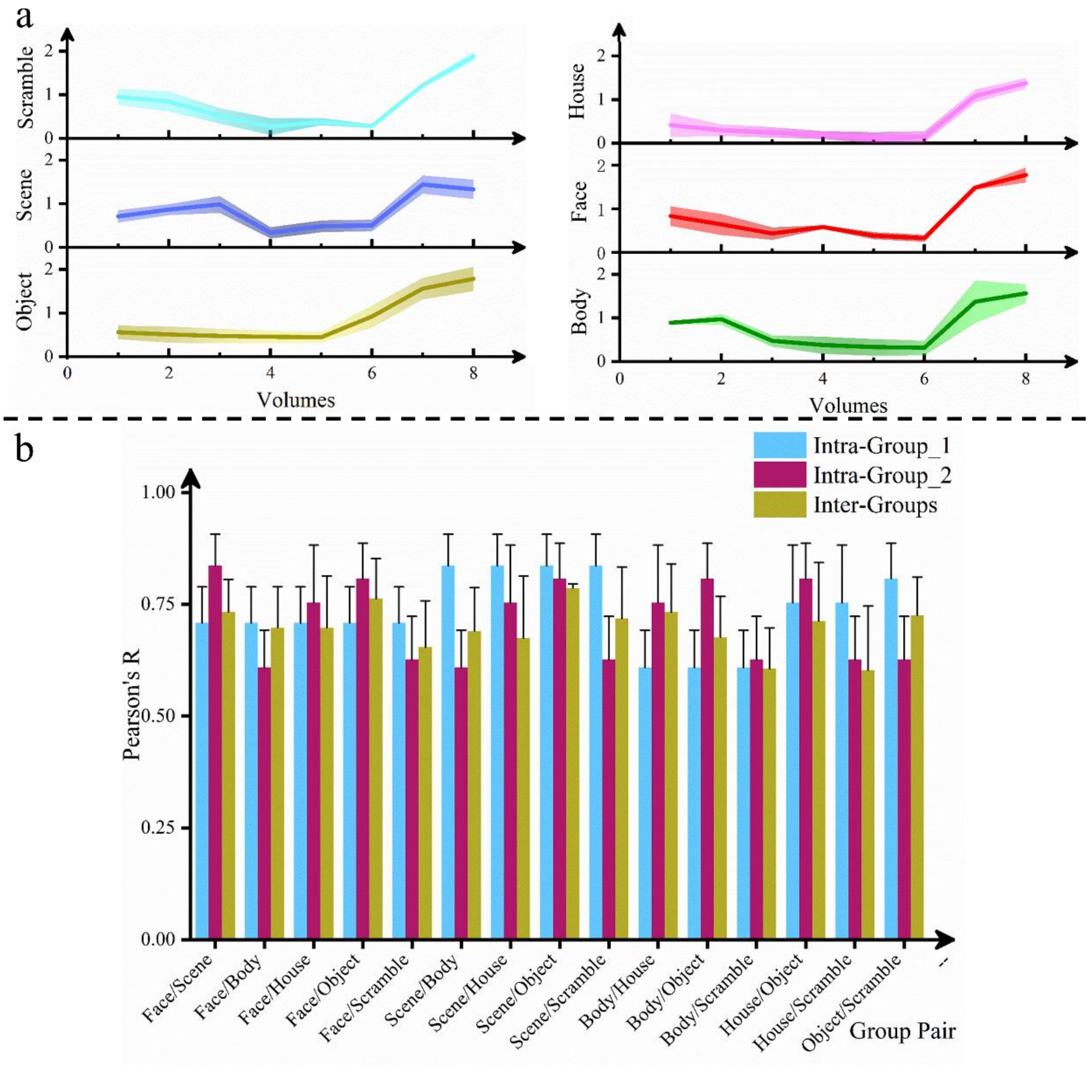

Supplementary Fig. 20 Dominating dynamics of six stimulus-responsive regions and their Pearson’s correlation analysis results for Subject _07.

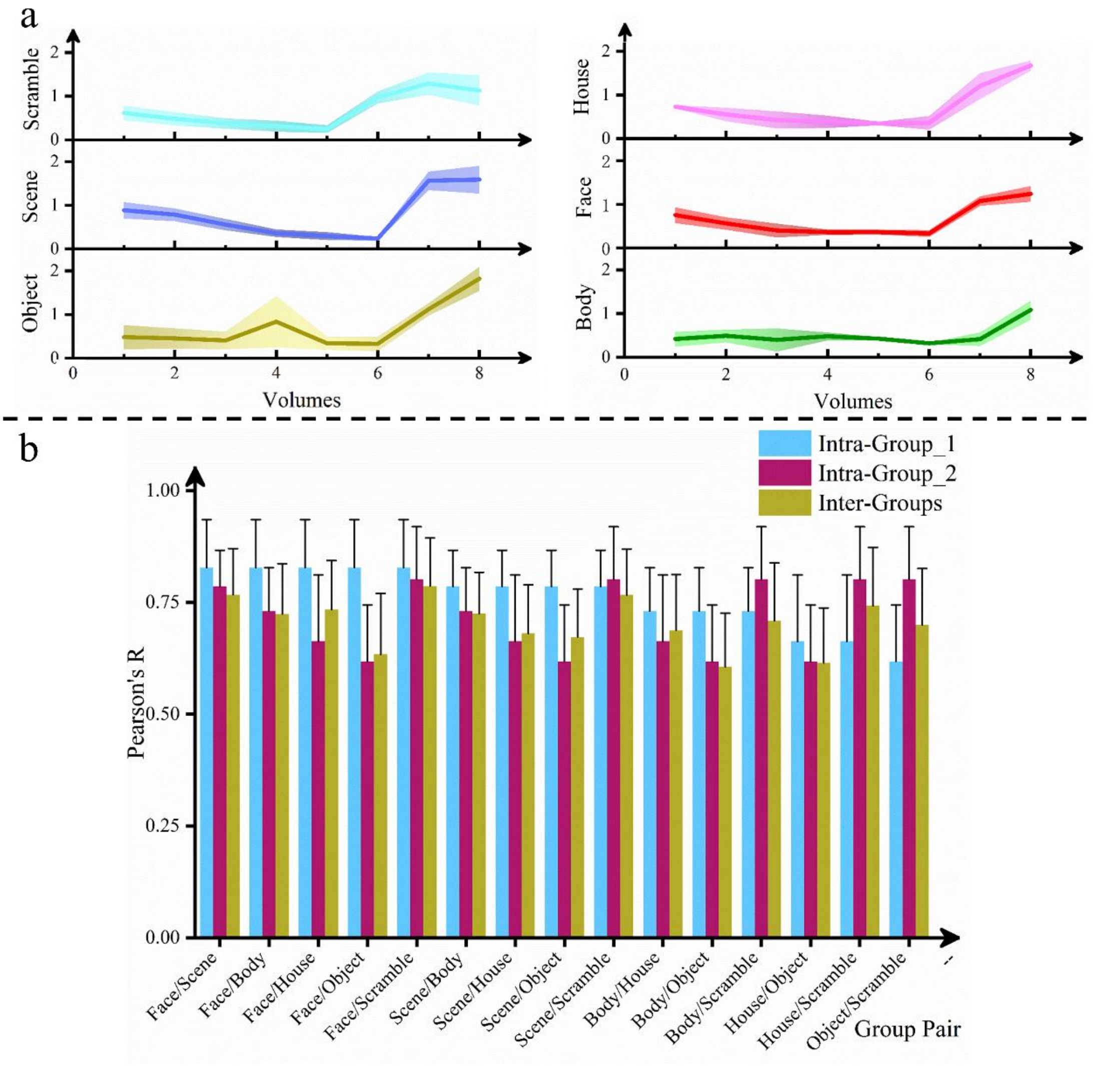

Supplementary Fig. 21 Dominating dynamics of six stimulus-responsive regions and their Pearson's correlation analysis results for Subject _08.

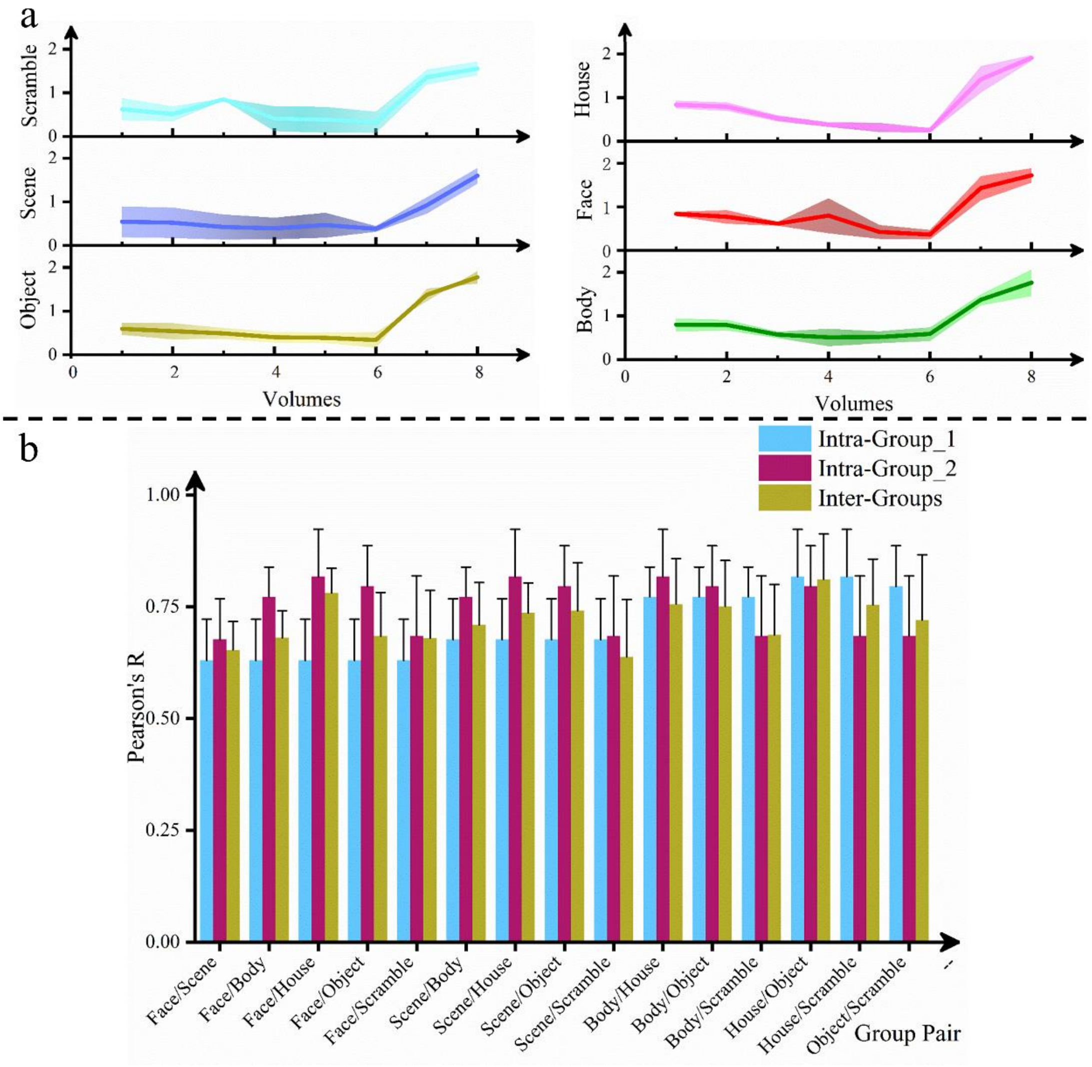

Supplementary Fig. 22 Dominating dynamics of six stimulus-responsive regions and their Pearson's correlation analysis results for Subject _09.

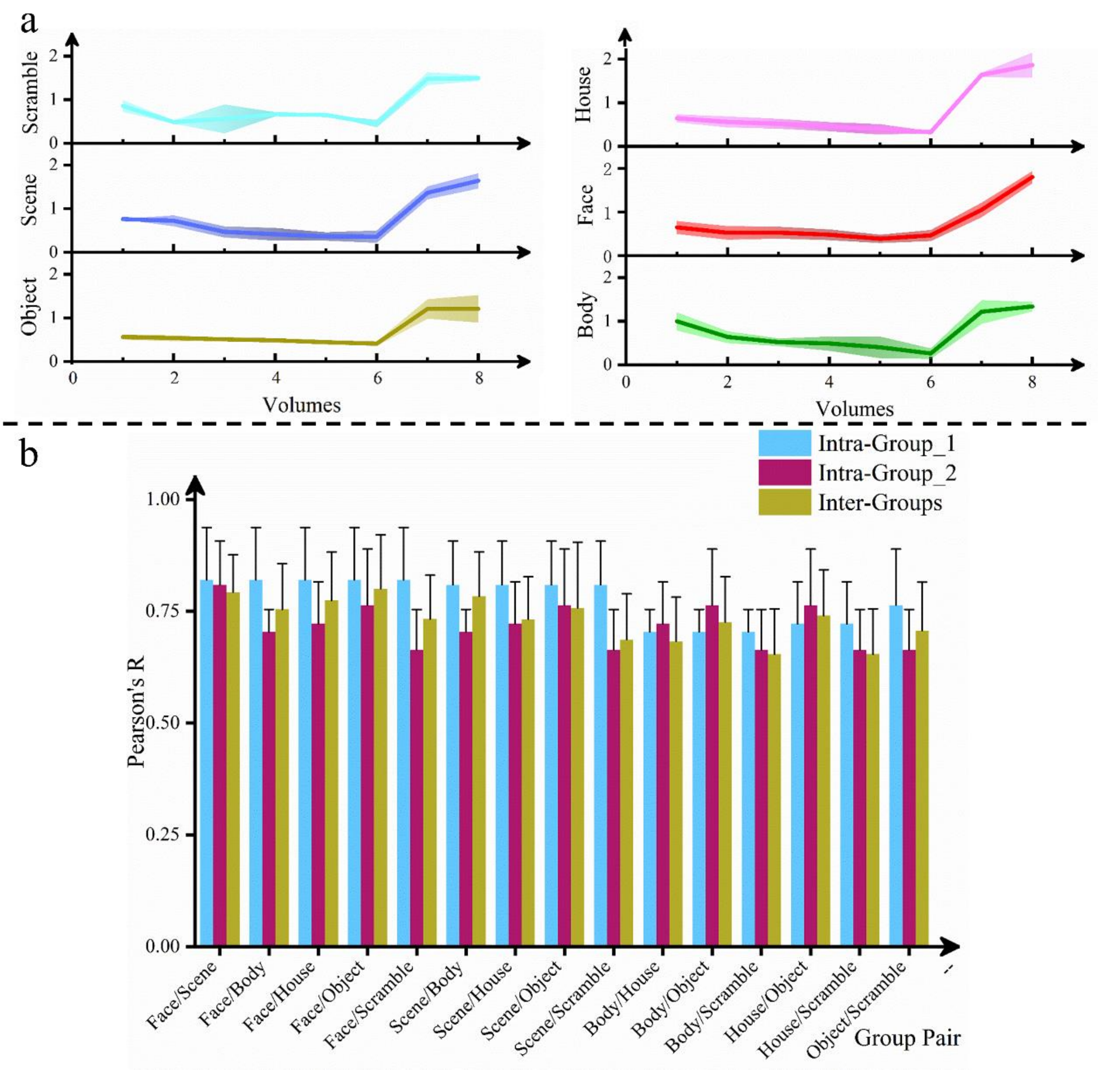

Supplementary Fig. 23 Dominating dynamics of six stimulus-responsive regions and their Pearson's correlation analysis results for Subject _10.

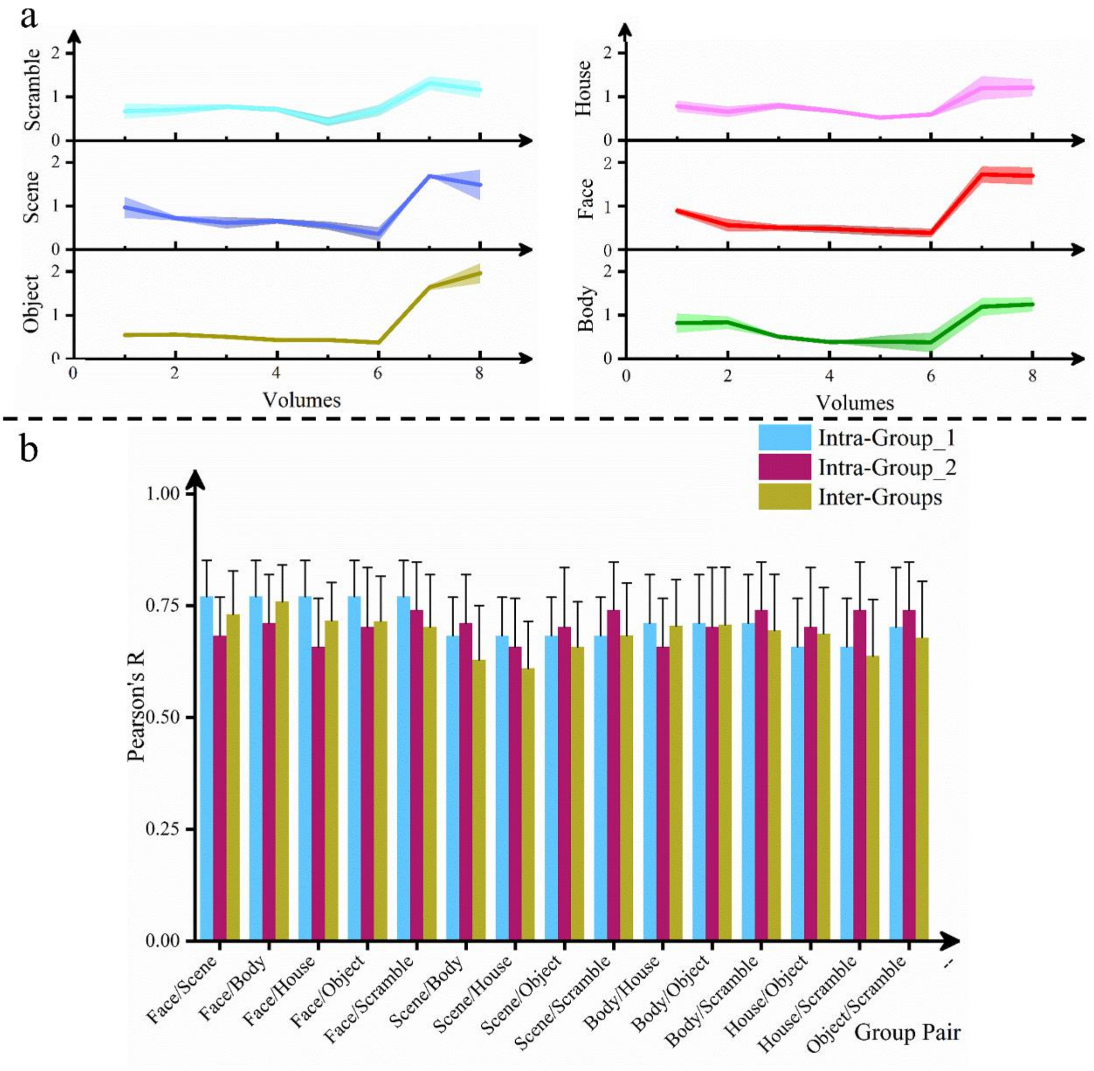

Supplementary Fig. 24 Dominating dynamics of six stimulus-responsive regions and their Pearson's correlation analysis results for Subject _11.

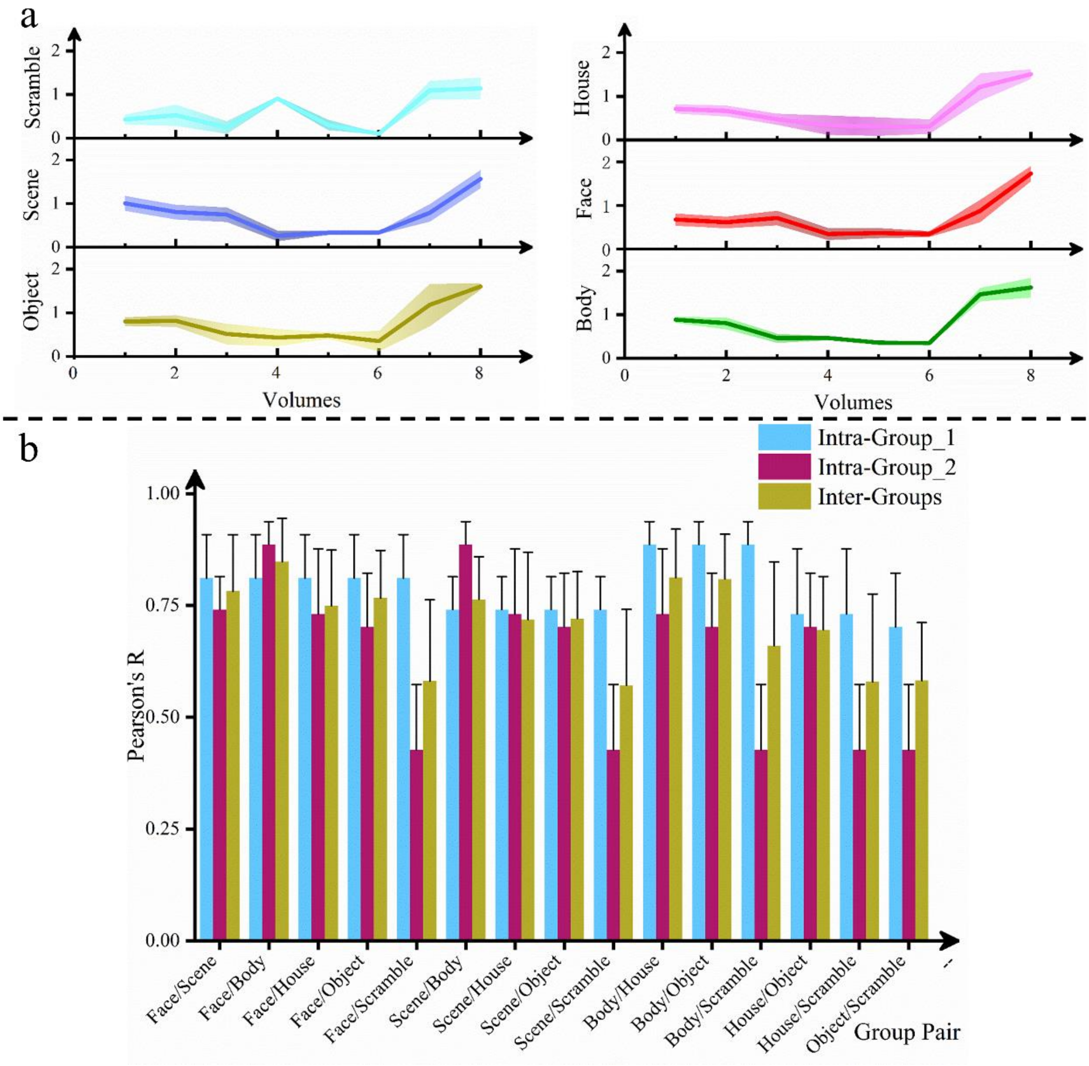

Supplementary Fig. 25 Dominating dynamics of six stimulus-responsive regions and their Pearson's correlation analysis results for Subject _12.

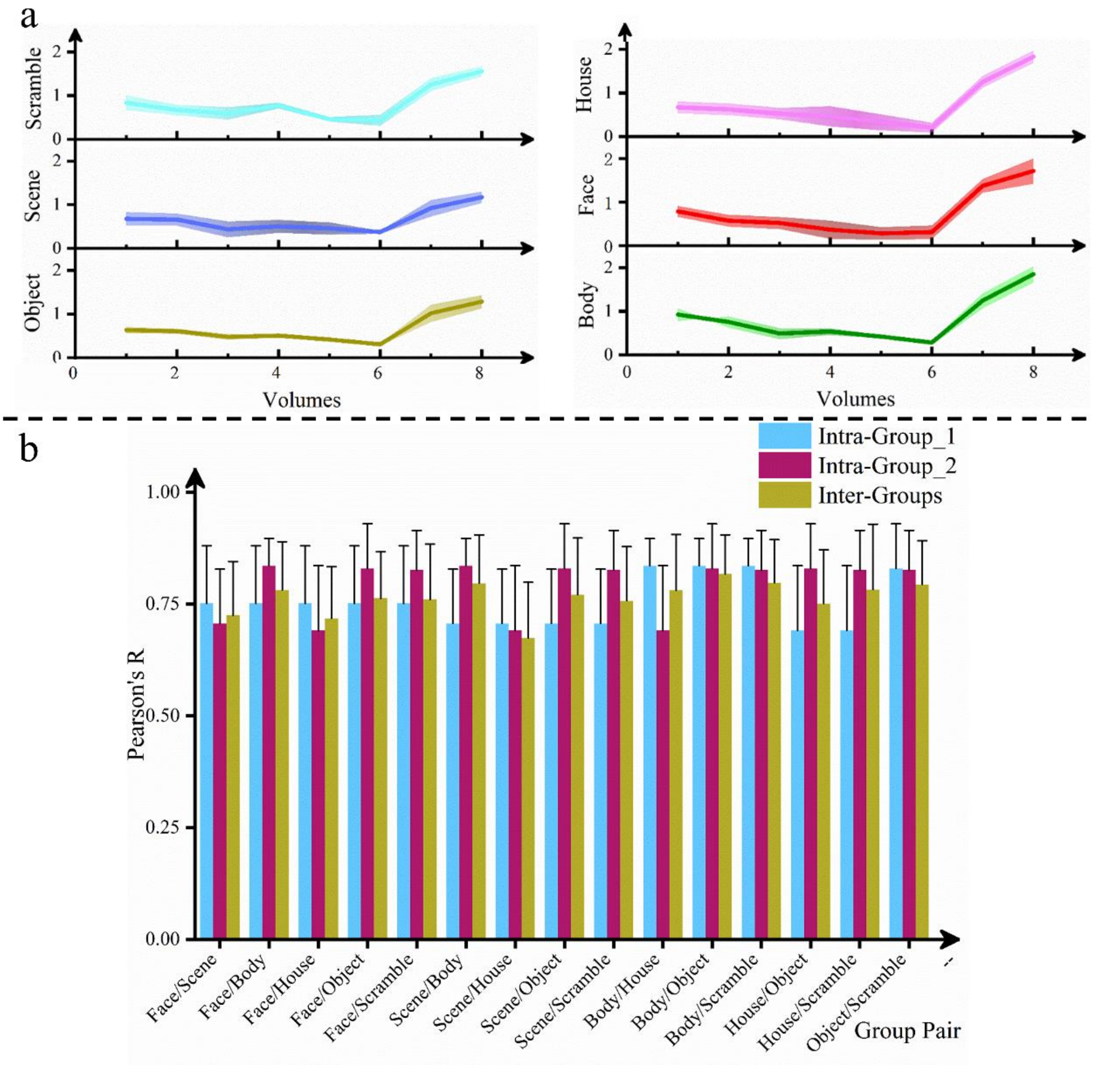

Supplementary Fig. 26 Dominating dynamics of six stimulus-responsive regions and their Pearson’s correlation analysis results for Subject _13.

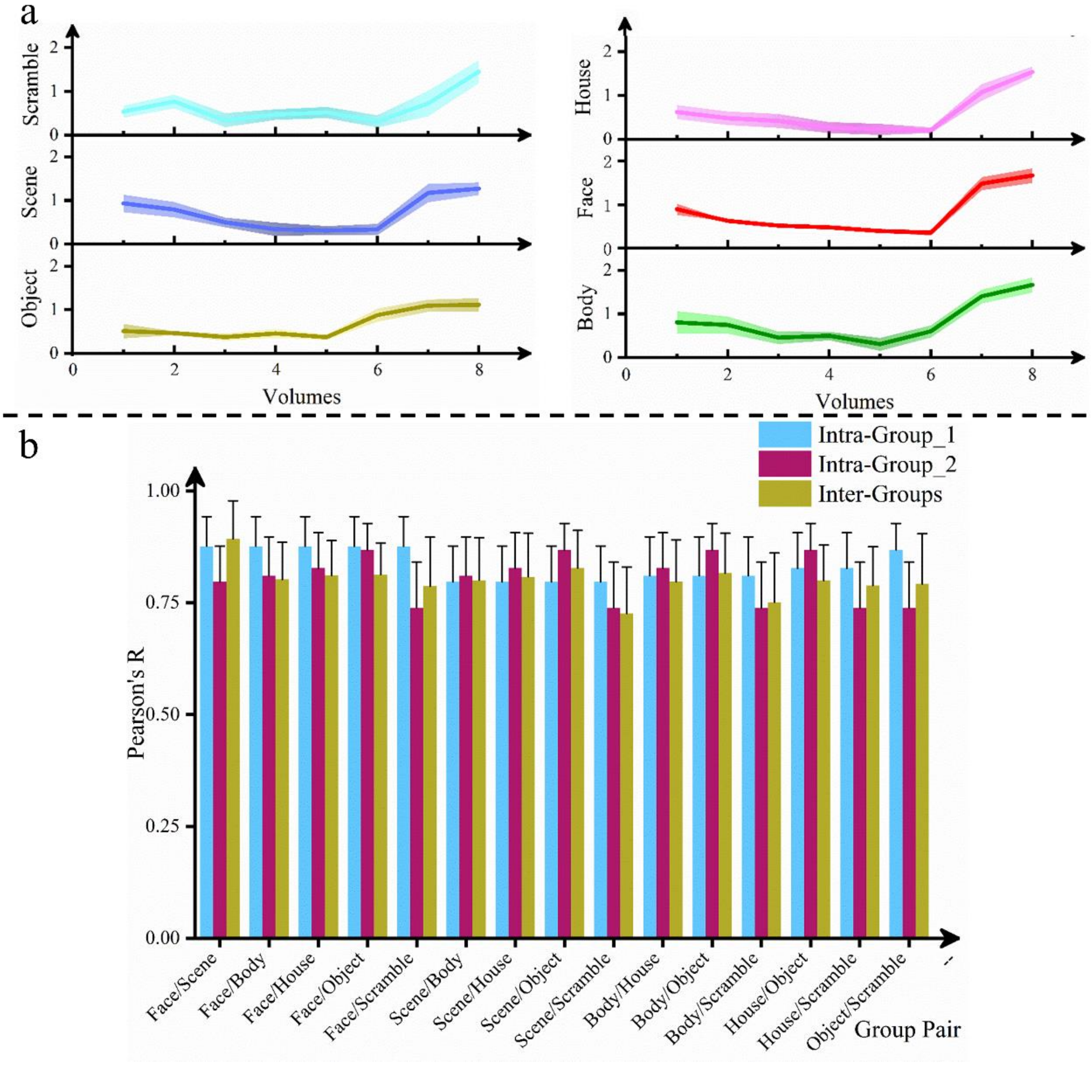

Supplementary Fig. 27 Dominating dynamics of six stimulus-responsive regions and their Pearson's correlation analysis results for Subject _14.

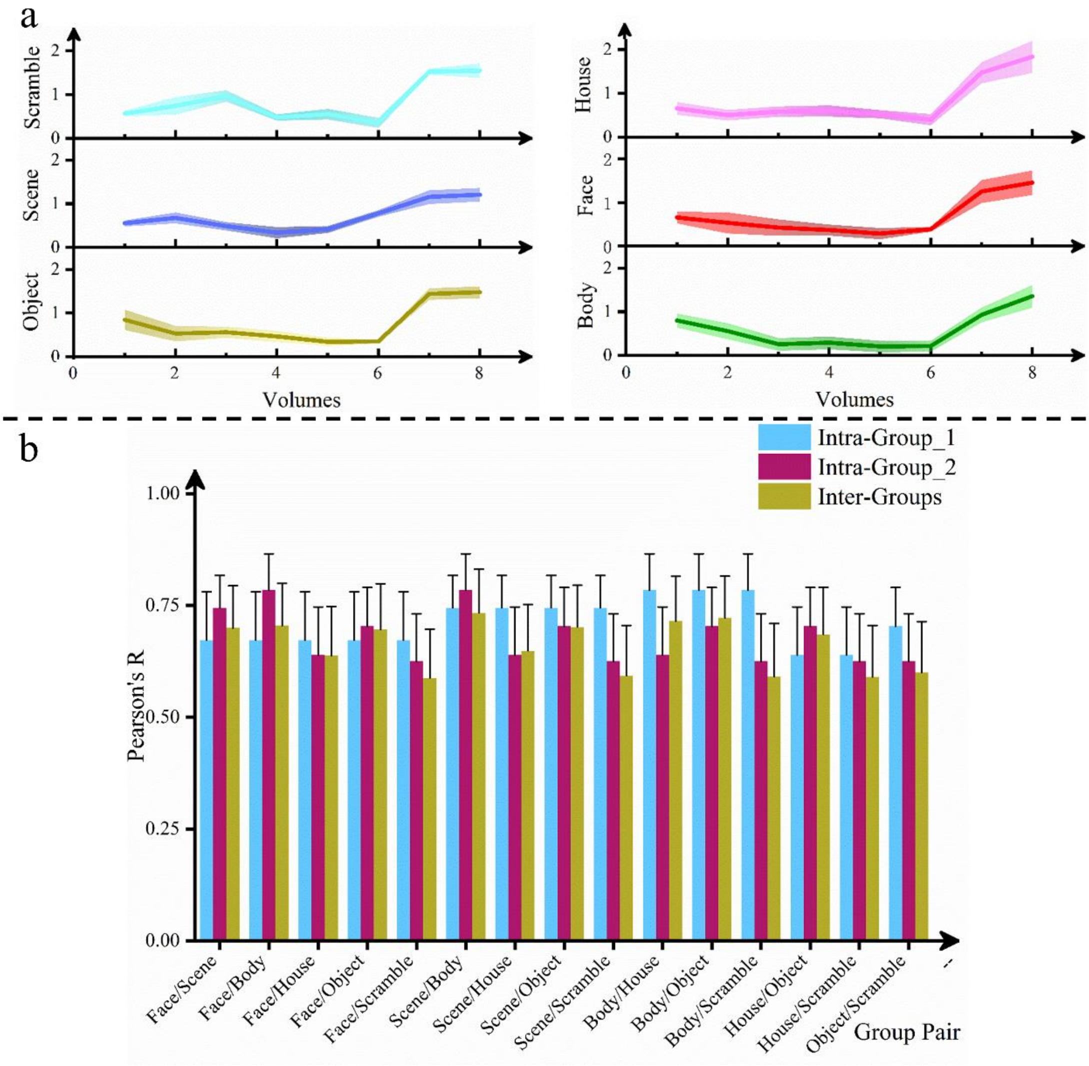

Supplementary Fig. 28 Dominating dynamics of six stimulus-responsive regions and their Pearson's correlation analysis results for Subject _15.

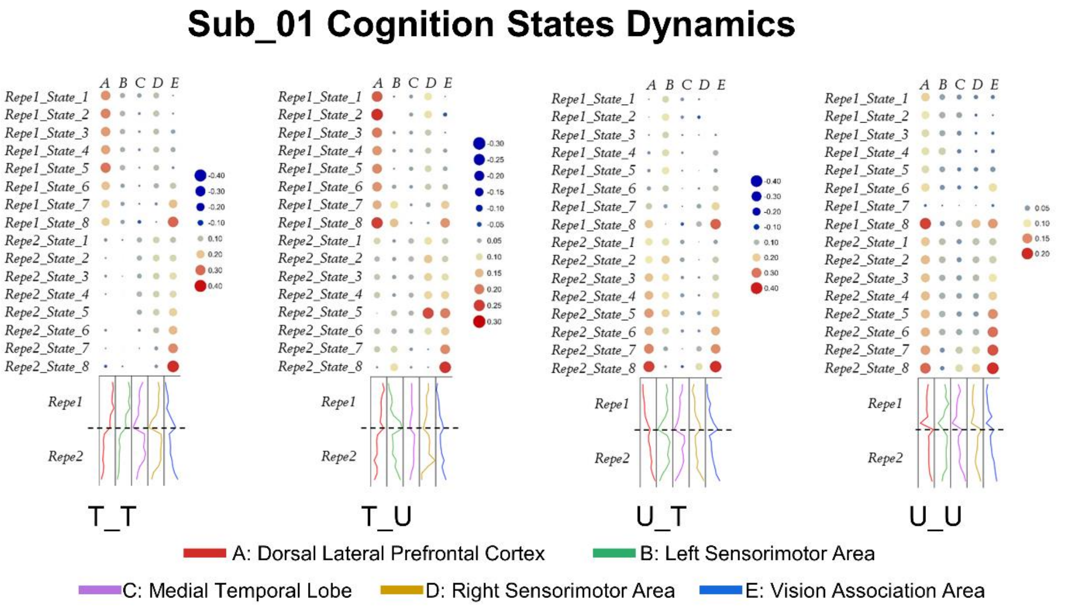

Supplementary Fig. 29 The personal cognitive state dynamics of the five clusters for Subject-01. The dashed lines denote the boundary of two continuous blocks of one run.

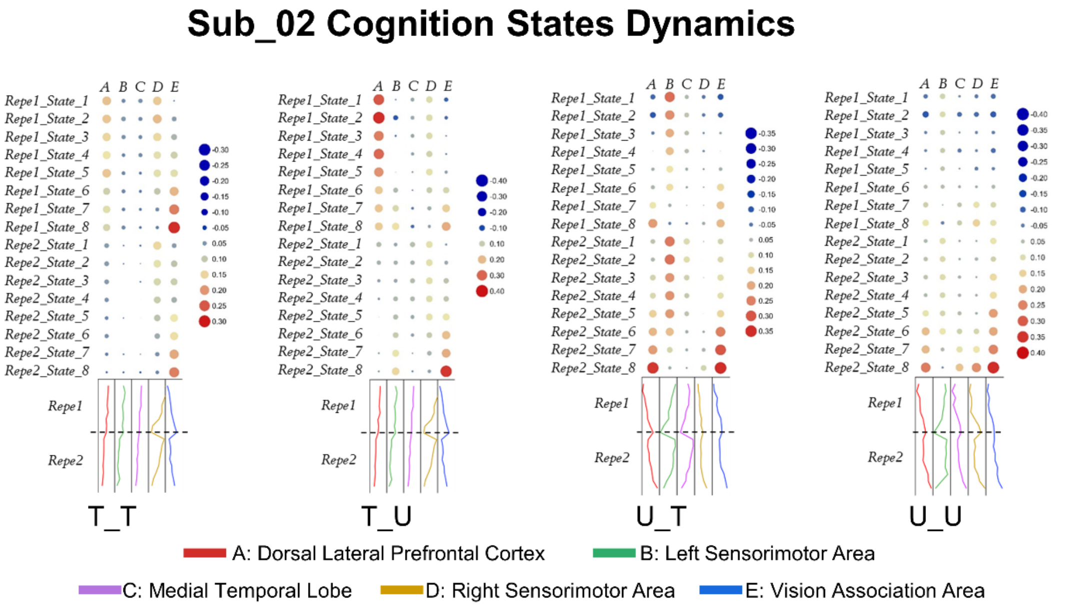

Supplementary Fig. 30 The personal cognitive state dynamics of the five clusters for Subject-02. The dashed lines denote the boundary of two continuous blocks of one run.

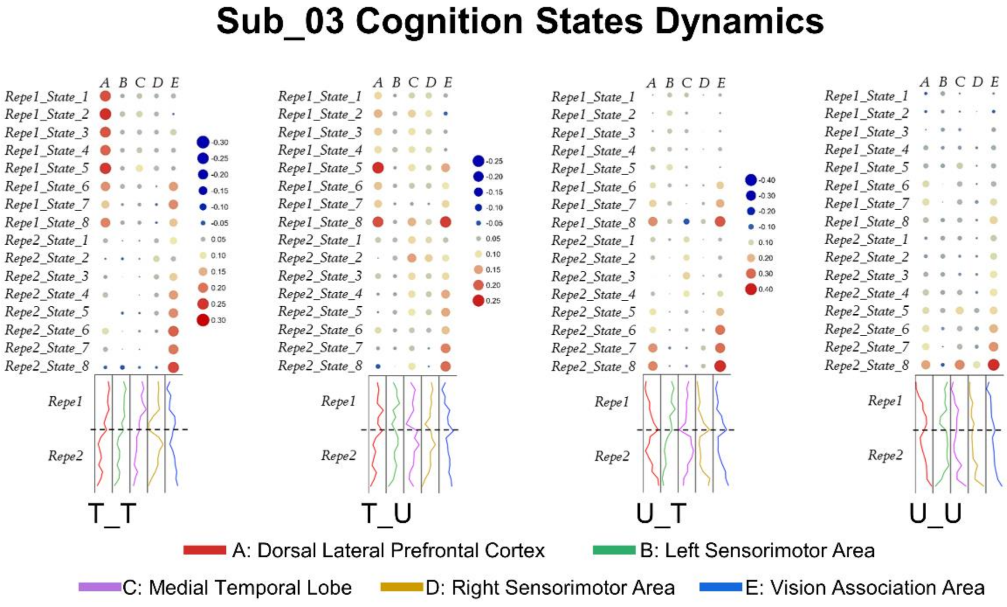

Supplementary Fig. 31 The personal cognitive state dynamics of the five clusters for Subject-03. The dashed lines denote the boundary of two continuous blocks of one run.

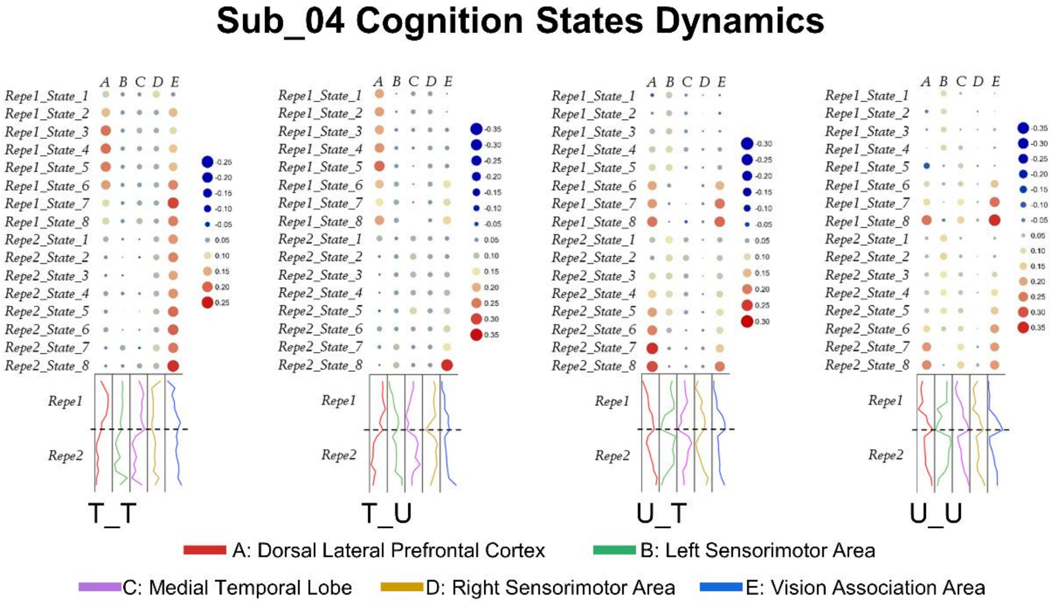

Supplementary Fig. 32 The personal cognitive state dynamics of the five clusters for Subject-04. The dashed lines denote the boundary of two continuous blocks of one run.

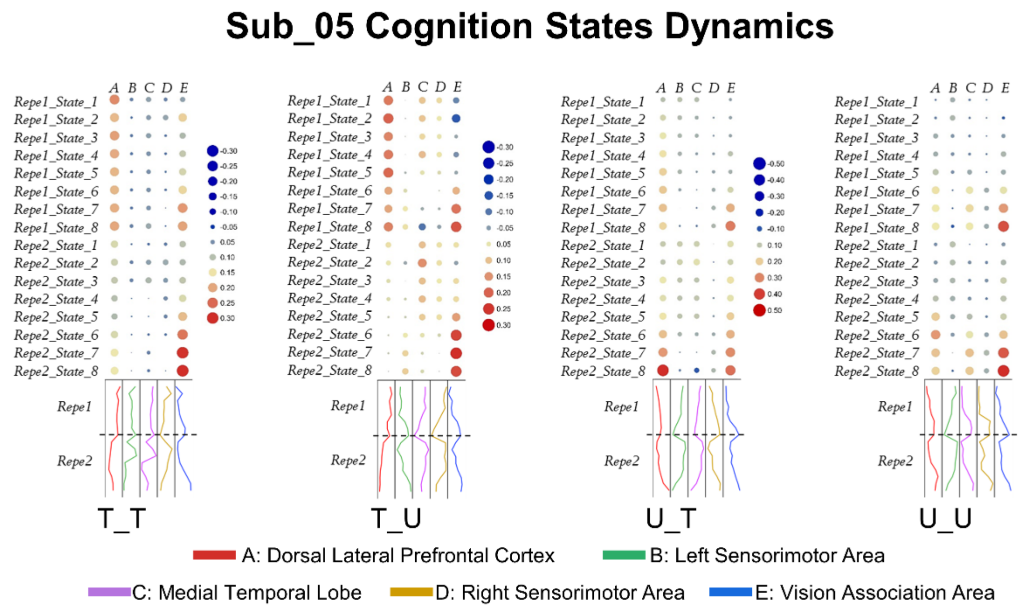

Supplementary Fig. 33 The personal cognitive state dynamics of the five clusters for Subject-05. The dashed lines denote the boundary of two continuous blocks of one run.

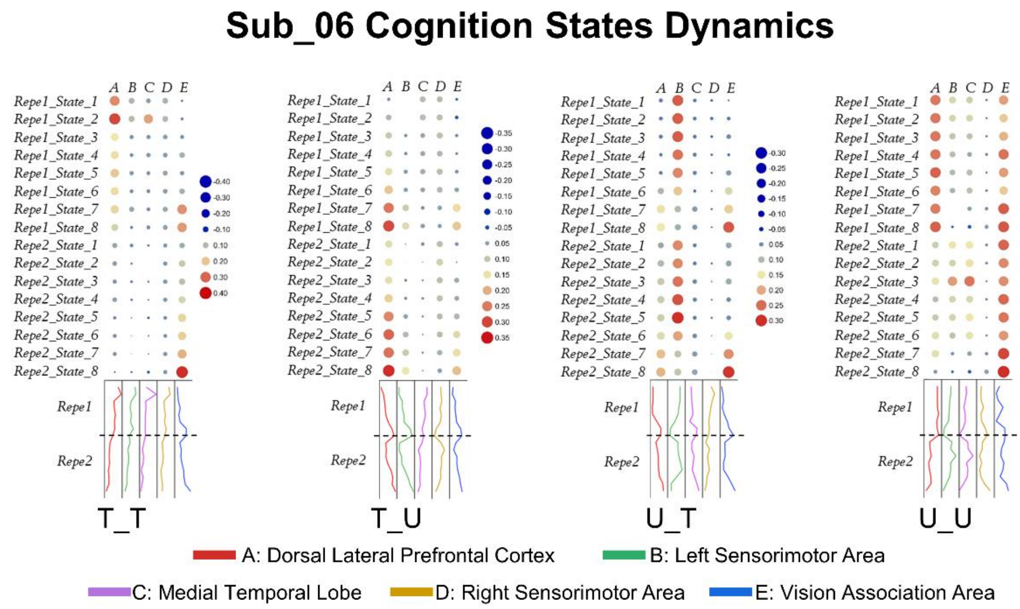

Supplementary Fig. 34 The personal cognitive state dynamics of the five clusters for Subject-06. The dashed lines denote the boundary of two continuous blocks of one run.

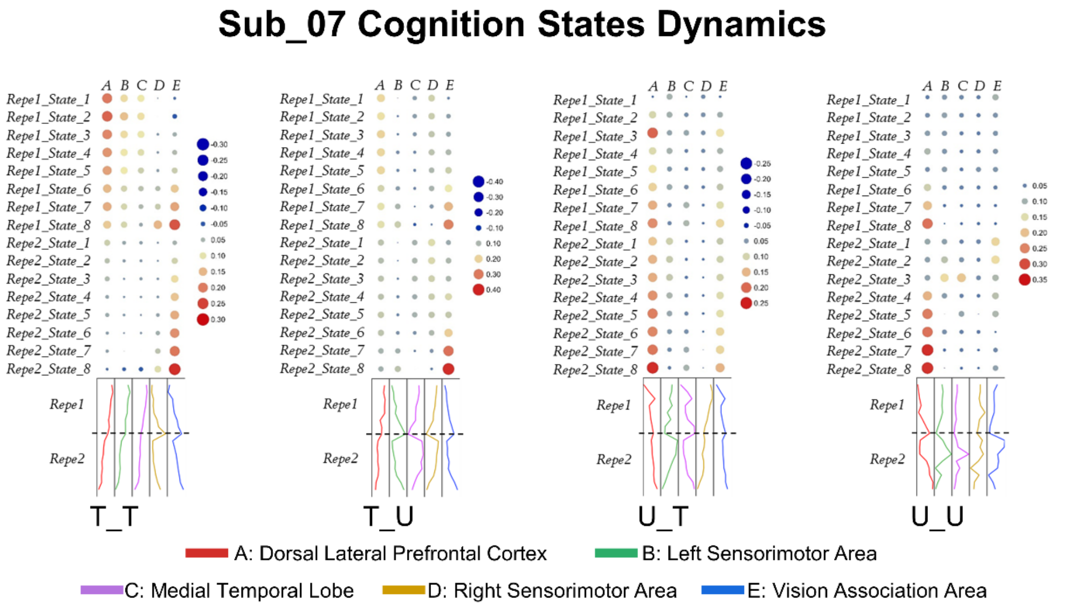

Supplementary Fig. 35 The personal cognitive state dynamics of the five clusters for Subject-07. The dashed lines denote the boundary of two continuous blocks of one run.

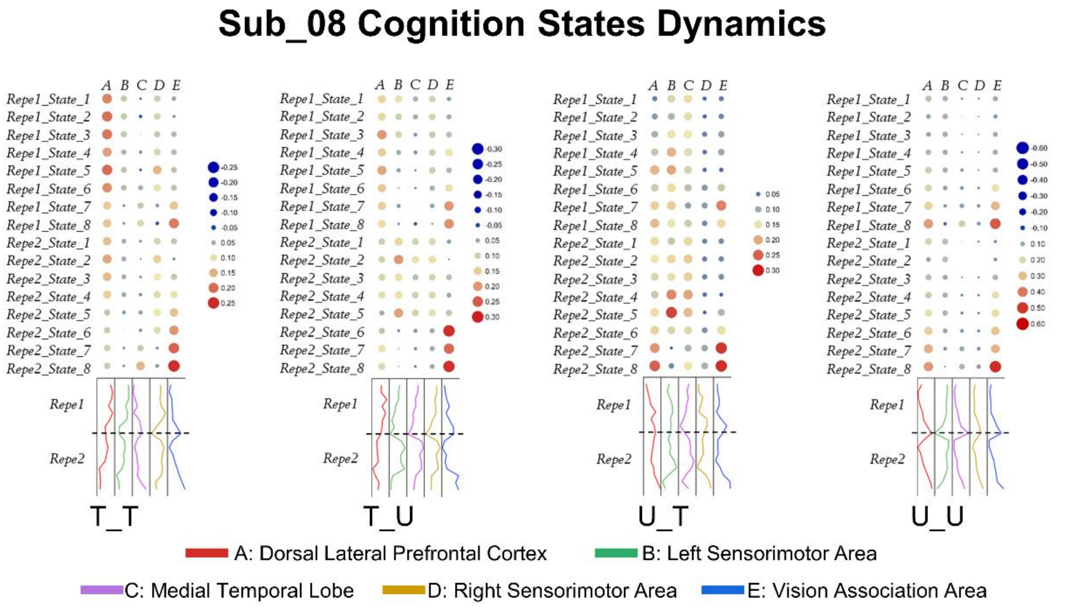

Supplementary Fig. 36 The personal cognitive state dynamics of the five clusters for Subject-08. The dashed lines denote the boundary of two continuous blocks of one run.

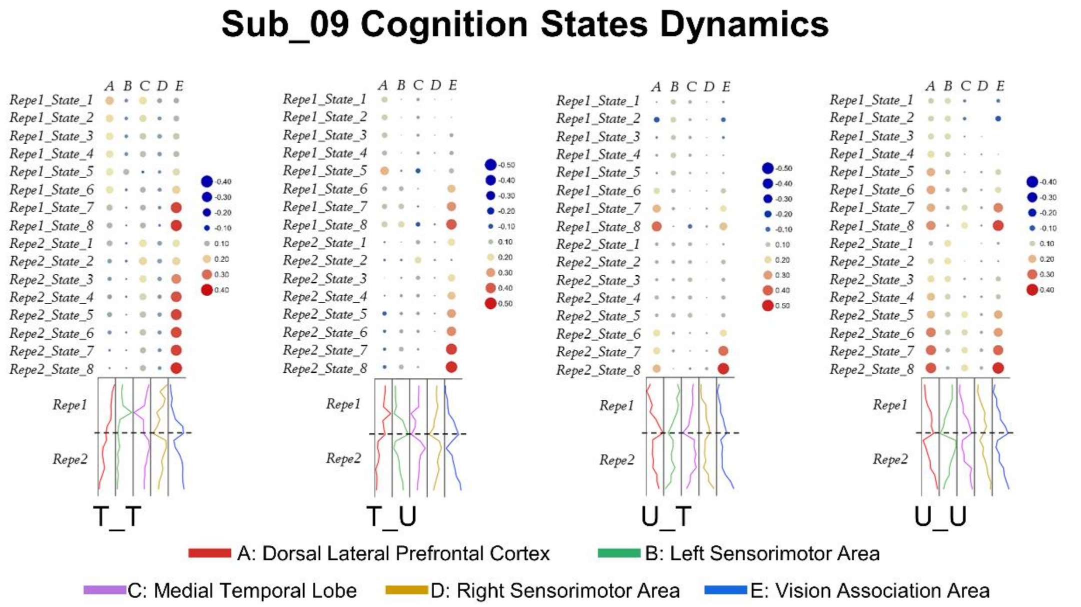

Supplementary Fig. 37 The personal cognitive state dynamics of the five clusters for Subject-09. The dashed lines denote the boundary of two continuous blocks of one run.

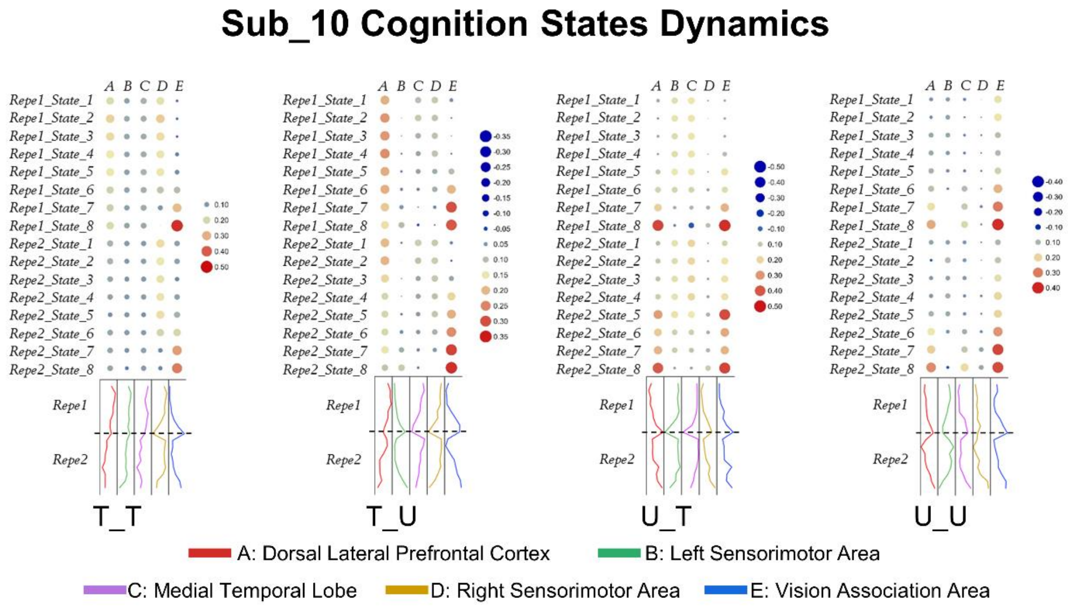

Supplementary Fig. 38 The personal cognitive state dynamics of the five clusters for Subject-10. The dashed lines denote the boundary of two continuous blocks of one run.

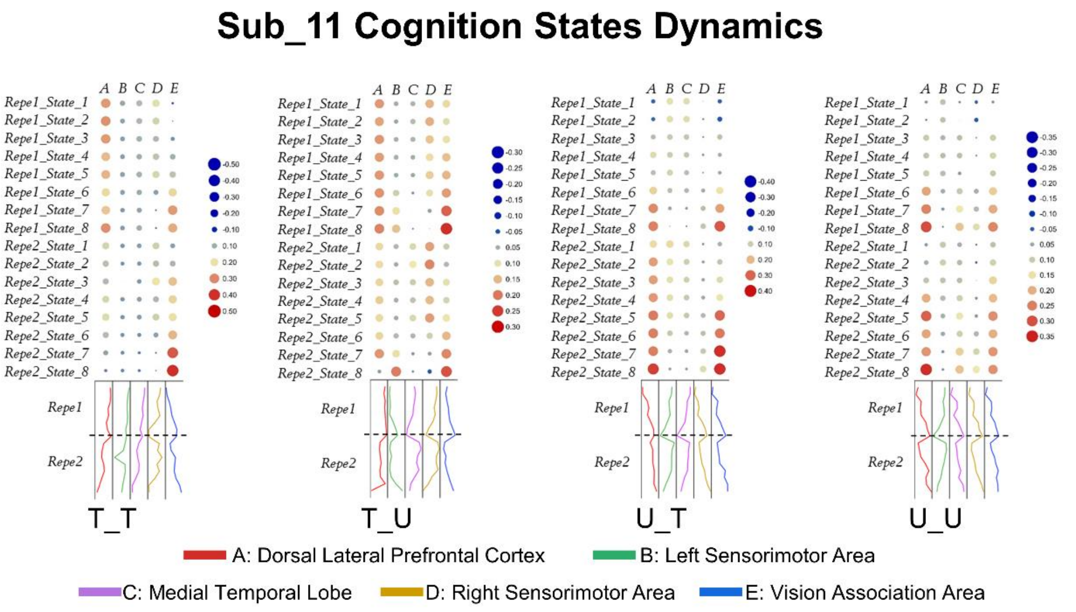

Supplementary Fig. 39 The personal cognitive state dynamics of the five clusters for Subject-11. The dashed lines denote the boundary of two continuous blocks of one run.

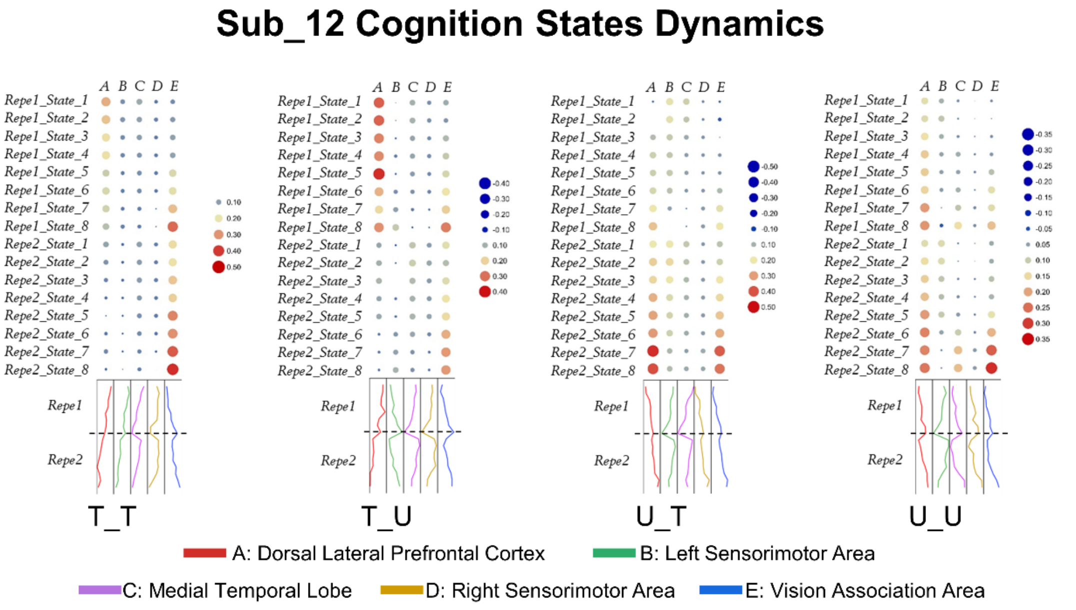

Supplementary Fig. 40 The personal cognitive state dynamics of the five clusters for Subject-12. The dashed lines denote the boundary of two continuous blocks of one run.

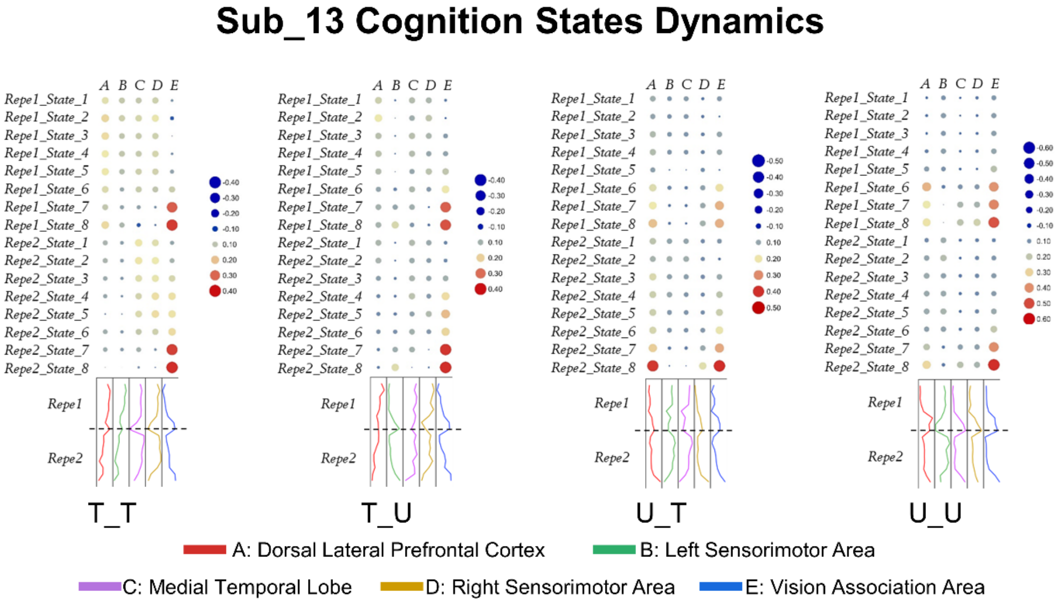

Supplementary Fig. 41 The personal cognitive state dynamics of the five clusters for Subject-13. The dashed lines denote the boundary of two continuous blocks of one run.

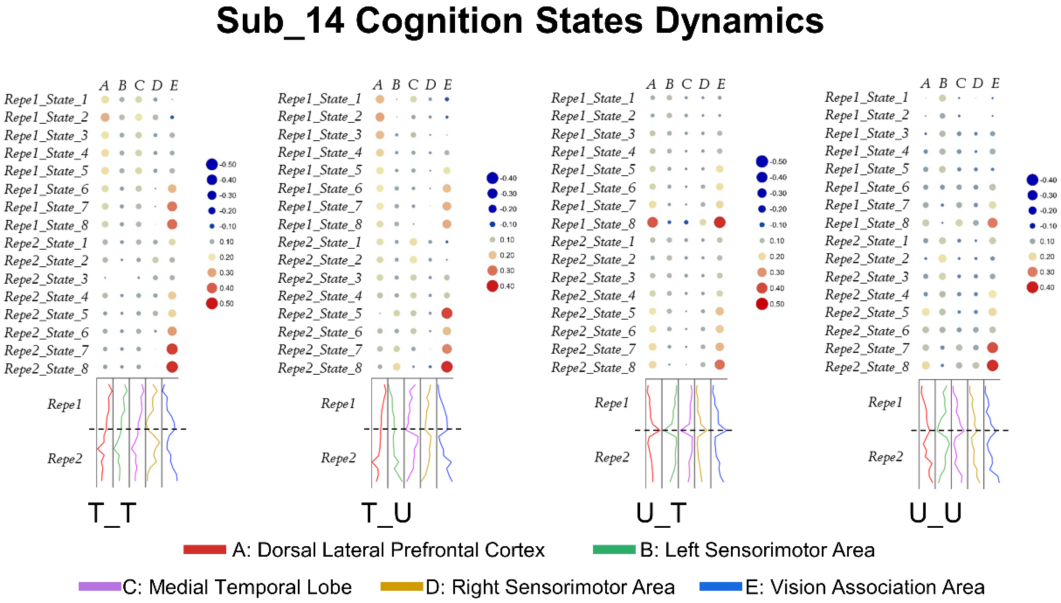

Supplementary Fig. 42 The personal cognitive state dynamics of the five clusters for Subject-14. The dashed lines denote the boundary of two continuous blocks of one run.

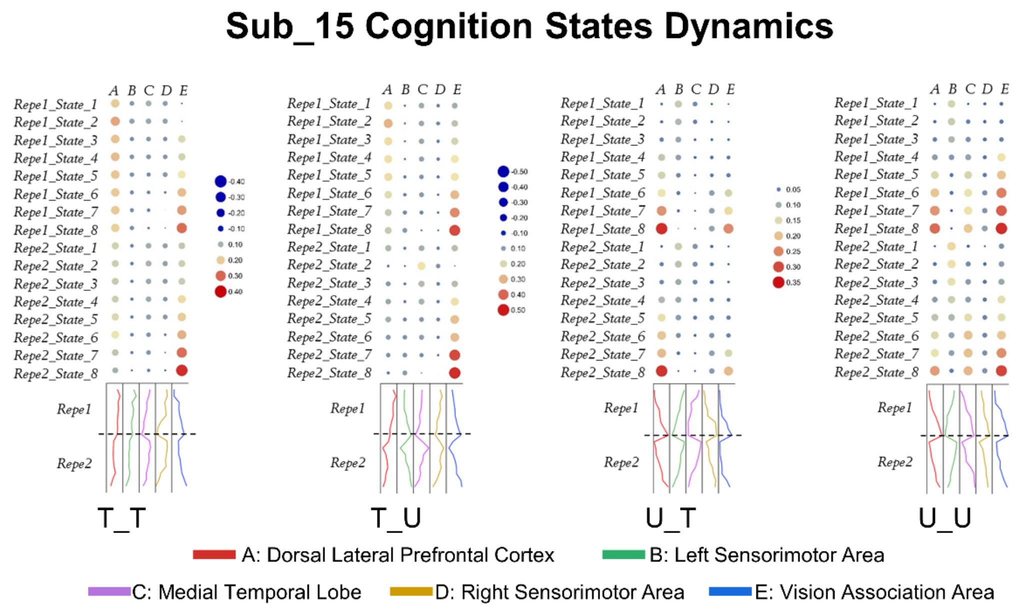

Supplementary Fig. 43 The personal cognitive state dynamics of the five clusters for Subject-15. The dashed lines denote the boundary of two continuous blocks of one run.

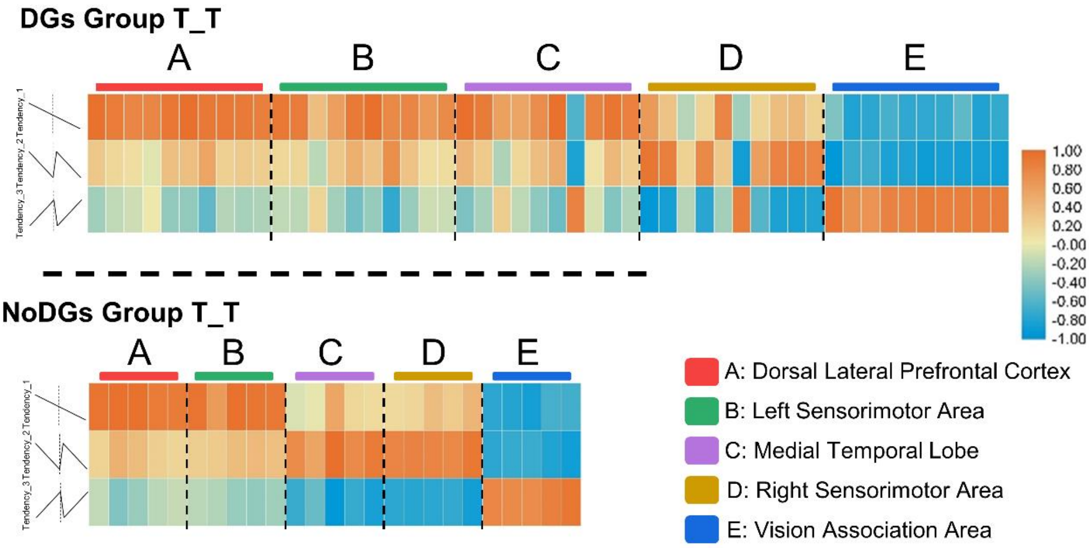

Supplementary Fig. 44 Pearson correlations analysis between the cognitive state dynamics in runs and three dominating tendencies across T_T condition for DGs Group (10 subjects, upper heatmap) and NoDGs Group (5 subjects, bottom heatmap).

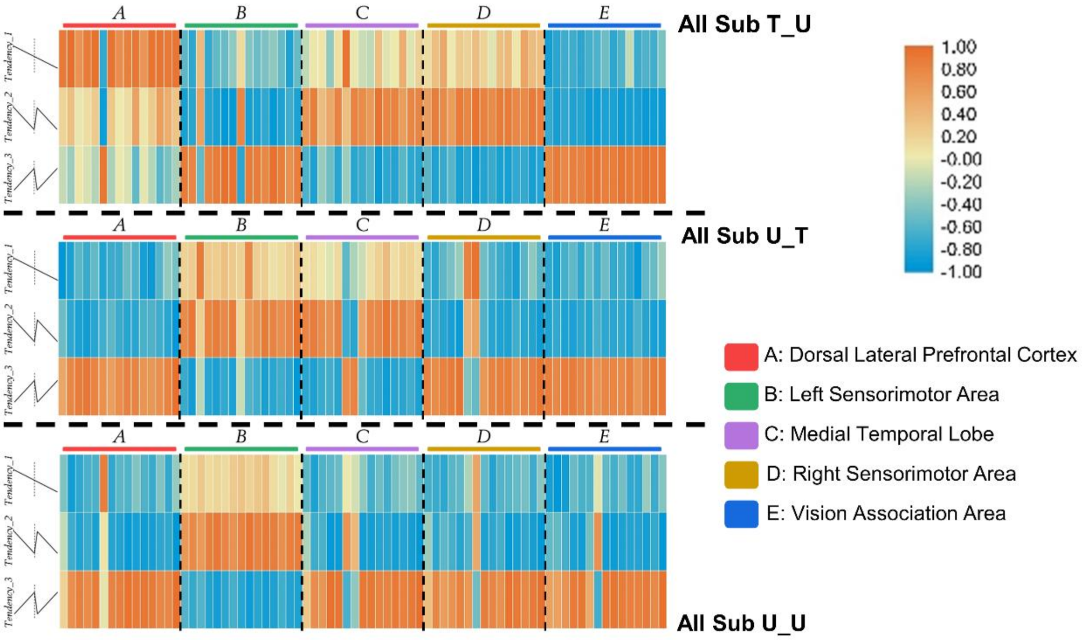

Supplementary Fig. 45 Pearson correlations analysis between the cognitive state dynamics in runs and three dominating tendencies across T_U (top heatmap), U_T (middle heatmap) and U_U (bottom heatmap) conditions for overall 15 subjects.
