# Supplementary material for: Variational relevance evaluation of individual fMRI data enables deconstruction of task-dependent neural dynamics
Source: Commun Biol. 2023 May 5;6:491. doi: 10.1038/s42003-023-04804-3 (PMC10163018; doi:10.1038/s42003-023-04804-3)
Supplement: Supplementary file 2 — Reporting Summary [file 42003_2023_4804_MOESM2_ESM.pdf]

nature portfolio

Corresponding author(s): COMMSBIO-23-0571A

Last updated by author(s): Mar 11, 2023

Reporting Summary

Nature Portfolio wishes to improve the reproducibility of the work that we publish. This form provides structure for consistency and transparency in reporting. For further information on Nature Portfolio policies, see our Editorial Policies and the Editorial Policy Checklist.

Statistics

For all statistical analyses, confirm that the following items are present in the figure legend, table legend, main text, or Methods section.

n/a	Confirmed	
☐	☒	The exact sample size (n) for each experimental group/condition, given as a discrete number and unit of measurement
☐	☒	A statement on whether measurements were taken from distinct samples or whether the same sample was measured repeatedly
☒	☐	The statistical test(s) used AND whether they are one- or two-sided<br>*Only common tests should be described solely by name; describe more complex techniques in the Methods section.*
☒	☐	A description of all covariates tested
☒	☐	A description of any assumptions or corrections, such as tests of normality and adjustment for multiple comparisons
☐	☒	A full description of the statistical parameters including central tendency (e.g. means) or other basic estimates (e.g. regression coefficient) AND variation (e.g. standard deviation) or associated estimates of uncertainty (e.g. confidence intervals)
☒	☐	For null hypothesis testing, the test statistic (e.g. F, t, r) with confidence intervals, effect sizes, degrees of freedom and P value noted<br>*Give P values as exact values whenever suitable.*
☐	☒	For Bayesian analysis, information on the choice of priors and Markov chain Monte Carlo settings
☒	☐	For hierarchical and complex designs, identification of the appropriate level for tests and full reporting of outcomes
☐	☒	Estimates of effect sizes (e.g. Cohen's d, Pearson's r), indicating how they were calculated

Our web collection on statistics for biologists contains articles on many of the points above.

Software and code

Policy information about availability of computer code

Data collection	The two task state fMRI datasets are both from published literatures. The higher visual areas localizer data were collected when the 15 participants viewed 144 pictures from 6 different classes under 3 Tesla fMRI circumstance. The procedural memory consolidation data were collected when 15 right-handed subjects performed the learned sequential tasks under 3 Tesla fMRI circumstance. Data collection was performed using the software Statistical Parametric Mapping 12 (SPM12) software package with MATLAB (R2018a) and the custom code developed in Python (3.6.4) environment.
Data analysis	Data analysis was performed using the software Statistical Parametric Mapping 12 (SPM12) software package with MATLAB (R2018a) and the custom code developed in Python (3.6.4) environment.

For manuscripts utilizing custom algorithms or software that are central to the research but not yet described in published literature, software must be made available to editors and reviewers. We strongly encourage code deposition in a community repository (e.g. GitHub). See the Nature Portfolio guidelines for submitting code & software for further information.

Data

Policy information about availability of data

All manuscripts must include a data availability statement. This statement should provide the following information, where applicable:

- Accession codes, unique identifiers, or web links for publicly available datasets
- A description of any restrictions on data availability
- For clinical datasets or third party data, please ensure that the statement adheres to our policy

The authors declare that the data supporting the findings of this study are available in the attached Supplementary Dataset.

Human research participants

Policy information about studies involving human research participants and Sex and Gender in Research.

Reporting on sex and gender	For the experiment of localizer for higher visual areas, 15 healthy subjects (mean age 29.4 years, 6 females) with proper vision were recruited according to the public data description of the published literature. For the procedural memory consolidation experiment, 15 healthy subjects (mean age ± std = 25.7±4.4 years, five females) were recruited according to the public data description of the published literature. No sex- or gender-based analyses were considered in this research, due to the focus on the individual difference instead of the sex or gender difference of the brain response to the same task performance.
Population characteristics	Please see above
Recruitment	N/A
Ethics oversight	All subjects provided written consent for each experiment which were approved by the local Ethics Committee according to the published literatures.

Note that full information on the approval of the study protocol must also be provided in the manuscript.

Field-specific reporting

Please select the one below that is the best fit for your research. If you are not sure, read the appropriate sections before making your selection.

☒ Life sciences ☐ Behavioural & social sciences ☐ Ecological, evolutionary & environmental sciences

For a reference copy of the document with all sections, see nature.com/documents/nr-reporting-summary-flat.pdf

Life sciences study design

All studies must disclose on these points even when the disclosure is negative.

Sample size	We designed an individual dynamical states detector model (VRE). To test the feasibility in classification accuracy and topology positions, we used 15 individual higher visual areas localizer fmri dataset. By exploiting the movement dataset after procedural memory consolidation for another 15 subjects, we decoupled different cognition functions into three diverse trans-regional enhanced or suppressed cognition states across five brain regions. The individual results demonstrated that the neural dynamic states revealed by VRE were highly reproducible across different subjects. These datasets are from published literatures. Sample size in the sense of classical statistical analysis is not applicable for this study.
Data exclusions	No data exclusions.
Replication	All results are produced by computer program and fully reproducible.
Randomization	N/A
Blinding	N/A

Reporting for specific materials, systems and methods

We require information from authors about some types of materials, experimental systems and methods used in many studies. Here, indicate whether each material, system or method listed is relevant to your study. If you are not sure if a list item applies to your research, read the appropriate section before selecting a response.

Materials & experimental systems

n/a	Involved in the study
☒	☐ Antibodies
☒	☐ Eukaryotic cell lines
☒	☐ Palaeontology and archaeology
☒	☐ Animals and other organisms
☒	☐ Clinical data
☒	☐ Dual use research of concern

Methods

n/a	Involved in the study
☒	☐ ChIP-seq
☒	☐ Flow cytometry
☒	☐ MRI-based neuroimaging
